# Pyridine–Quinoline and Biquinoline-Based Ruthenium *p*-Cymene Complexes as Efficient Catalysts for Transfer Hydrogenation Studies: Synthesis and Structural Characterization

**DOI:** 10.3390/molecules30142945

**Published:** 2025-07-11

**Authors:** Nikolaos Zacharopoulos, Gregor Schnakenburg, Eleni I. Panagopoulou, Nikolaos S. Thomaidis, Athanassios I. Philippopoulos

**Affiliations:** 1 Laboratory of Inorganic Chemistry, Department of Chemistry, National and Kapodistrian University of Athens, Panepistimiopolis Zografou, 15771 Athens, Greece; nzachar@chem.uoa.gr; 2 Institut für Anorganische Chemie, Rheinische Friedrich-Wilhelms-Universität Bonn, Gerhard-Domagk-Straße 1, D-53121 Bonn, Germany; gschnake@uni-bonn.de; 3 Laboratory of Analytical Chemistry, Department of Chemistry, National and Kapodistrian University of Athens, Panepistimiopolis Zografou, 15771 Athens, Greece; elenapanag@chem.uoa.gr (E.I.P.); ntho@chem.uoa.gr (N.S.T.)

**Keywords:** pyridine–quinoline ligands, *p*-cymene, ruthenium complexes, ketones, transfer hydrogenation, hydrogenation, hydride species

## Abstract

Searching for new and efficient transfer hydrogenation catalysts, a series of new organometallic ruthenium(II)-arene complexes of the formulae [Ru(η^6^-*p*-cymene)(L)Cl][PF_6_] (**1**–**8**) and [Ru(η^6^-*p*-cymene)(L)Cl][Ru(η^6^-*p*-cymene)Cl_3_] (**9**–**11**) were synthesized and fully characterized. These were prepared from the reaction of pyridine–quinoline and biquinoline-based ligands (L) with [Ru(η^6^-*p*-cymene)(μ-Cl)Cl]_2_, in 1:2 and 1:1, metal (M) to ligand (L) molar ratios. Characterization includes a combination of spectroscopic methods (FT-IR, UV-Vis, multi nuclear NMR), elemental analysis and single-crystal X-ray crystallography. The pyridine–quinoline organic entities encountered, were prepared in high yield either via the thermal decarboxylation of the carboxylic acid congeners, namely 2,2′-pyridyl-quinoline-4-carboxylic acid (**pqca**), 8-methyl-2,2′-pyridyl-quinoline-4-carboxylic acid (**8-Mepqca**), 6′-methyl-2,2′-pyridyl-quinoline-4-carboxylic acid (**6′-Mepqca**) and 8,6′-dimethyl-2,2′-pyridyl-quinoline-4-carboxylic acid (**8,6′-Me_2_pqca**), affording the desired ligands **pq**, **8-Mepq, 6′-Mepq** and **8,6′-Me_2_pq**, or by the classical Friedländer condensation, to yield 4,6′-dimethyl-2,2′-pyridyl-quinoline (**4,6′-Me_2_pq**) and 4-methyl-2,2′-pyridyl-quinoline (**4-Mepq**), respectively. The solid-state structures of complexes **1**–**4**, **6, 8** and **9** were determined showing a distorted octahedral coordination geometry. The unit cell of **3** contains two independent molecules (Ru-**3**), (Ru′-**3**) in a 1:1 ratio, due to a slight rotation of the arene ring. All complexes catalyze the transfer hydrogenation of acetophenone, using 2-propanol as a hydrogen donor in the presence of KO*i*Pr. Among them, complexes **1** and **5** bearing methyl groups at the 8 and 4 position of the quinoline moiety, convert acetophenone to 1-phenylethanol quantitatively, within approximately 10 min with final TOFs of 1600 h^−1^. The catalytic performance of complexes **1**–**11**, towards the transfer hydrogenation of *p*-substituted acetophenone derivatives and benzophenone, ranges from moderate to excellent. An inner-sphere mechanism has been suggested based on the detection of ruthenium(II) hydride species.

## 1. Introduction

Hydrogenation (HY) is a fundamental chemical process with a plethora of applications [1], which span from the food (fat hydrogenation) and petrochemical industries to the cosmetics and pharmaceutical industries, along with the production of ammonia and other chemical products [2]. Independently, transfer hydrogenation (TH) has been developed as an alternative to classic hydrogenation [3,4]. In this process, 2-propanol or formic acid can be used as sacrificial hydrogen donors, different to molecular hydrogen. Notably, this is a benign and eco-friendly method where no high pressure of molecular hydrogen is required and, in general, all hydrogen donors are readily available and cheap [5].

Early reports by Henbest and Mitchell [6], Sasson and Blum [7] and Backvall [8] are the cornerstones of this chemical process with potential applications not only in academia but in the chemical industry as well. The pioneering work of Noyori [9], in the field of asymmetric TH, has opened new avenues in homogenous catalysis with the development of new enantioselective transfer hydrogenation ruthenium(II) catalysts ([(η^6^-arene)Ru(Tsdpen)Cl]; (H)Tsdpen = N-p-tosyl-1,2-diphenylethylenediamine). Towards this goal, a plethora of metal complexes has been synthesized and evaluated as catalysts [10]. Their ability to convert multiple polarized unsaturated substrates to compounds with constantly increasing applications in industry (pharmaceutical, agrochemical, fragrance) demonstrates the high importance of transfer hydrogenation reactions [11]. In this respect, we may comment on new potential applications of the TH reactions such as biofuels production [12], along with a new approach for cancer therapy, via intracellular TH reactions [13,14].

This great diversity among proposed catalysts has led to the suggestion of different mechanistic routes. In general, these can be divided as “inner” and “outer sphere” mechanisms, including their sub-categories [15,16]. In this respect, it has been suggested that the structure of the organic ligands attached to the metallic center plays a significant role in the mechanistic route and the overall activity of the catalyst [17].

Among the plethora of metal complexes that served in the transfer hydrogenation reaction of different substrates, organometallic ruthenium(II) complexes comprising the η^6^-arene ligand, constitute an interesting class of catalyst precursors [3]. The coordination sphere around the metal center can be filled with different organic ligands like NHCs (N-heterocycle carbenes) [18], phosphines [19], thioamides [20], N/P [21], N/O [22], N,N pincer ligands along with N^N ligands (N^N = bidentate), either amino or pyridyl based [23,24,25,26,27], while a series of 2-(2-quinolyl)benzimidazole-based ruthenium(II) complexes containing 8-amino-quinoline derivatives have been used for TH reactions [28,29].

In line with what we have reported previously, a series of pyridine–quinoline based ruthenium(II) catalyst precursors bearing triphenyl phosphine ligands, of the formula cis-[RuCl_2_(PPh_3_)_2_(Lx)] (Lx = **pq** = 2,2′-pyridyl-quinoline; **4-Mepq =** 4-methyl-2,2′-pyridyl-quinoline; and **pqcame =** methyl 2,2′-pyridyl-quinoline-4-carboxylate) which were used for the transfer hydrogenation of polarized unsaturated substrates [30,31,32]. We report herein the synthesis, spectroscopic and structural characterization of a series of ruthenium(II) arene complexes of the general formula [Ru(η^6^-*p*-cymene)(L)Cl][PF_6_] (L = 8-Mepq (**1**), 6′-Mepq (**2**), 8,6′-Me_2_pq (**3**), 4,6′-Me_2_pq (**4**), 4-Mepq (**5**), biqcame (**6**), biq (**8**) and [Ru(η^6^-*p*-cymene)(L)Cl][Ru(η^6^-*p*-cymene)Cl_3_] (L = pqcame (**9**), 4-Mepq (**10**), pq (**11**)). The catalytic activities of all complexes, including those of the known complexes **Ru-pqcame** [33], **7-Cl** [34] and **8** [35] have been examined in the transfer hydrogenation reaction of various aromatic ketones, that are used as substrates. The possible effects related to the position of methyl groups around the ligand periphery, along with that of the different counter anion encountered ([PF_6_]^−^ or [Ru(η^6^-*p*-cymene)Cl_3_]^−^), on the catalytic performance of the catalyst precursors, have been also studied.

## 2. Results and Discussion

### 2.1. Synthesis, Spectroscopic and Structural Characterization of the Organic Ligands

The organic ligands 2,2′-pyridyl-quinoline (**pq**) [36], 8-methyl-2,2′-pyridyl-quinoline (**8-Mepq**), 6′-methyl-2,2′-pyridyl-quinoline (**6′-Mepq**) and 8,6′-dimethyl-2,2′-pyridyl-quinoline (**8,6′-Me_2_pq**) were synthesized from the relevant carboxylic acid congeners **pqca**, **Μepqca**, **6′-Mepqca** and **8,6′-Me_2_pqca** with slight modifications of the general route described by Denny et al. for the case of **8-Mepq** [37]. In our efforts, thermal decarboxylation occurs without the presence of metallic copper, providing the final products as off-white or light-yellow solids, in higher yields compared to the published procedures. The synthesis of **6′-Mepqca** ligand has been reported by Haginiwa et al. [38], and more recently by Fan et al. [39], where in the latter, the preparation of **8,6′-Me_2_pqca** is also included. The 2,2′-biquinoline (**biq**) ligand was also synthesized for comparison [40,41,42]. The spectroscopic data (IR and ^1^H NMR) of the organic ligands prepared are in accord with those of the published procedures.

**Scheme 1:**
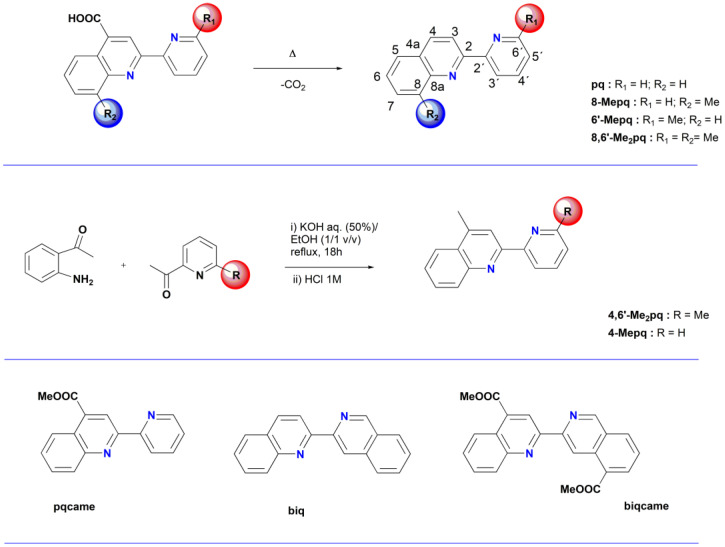
Synthesis and structures of organic ligands used in this work; hydrogen atom numbering is included for ^1^H NMR assignment.

The **4,6′-Me_2_pq** ligand [38] was reported previously, and characterization was based only on mass spectrometric data. In this study, we present a complete spectroscopic characterization of this ligand, including an X-ray structure determination. This compound was prepared according to the procedure described for the synthesis of **4-Mepq** [31], following the Friedländer condensation reaction of 2-aminoacetophenone with 2-acetyl-pyridine. The syntheses of the methyl ester analogs, methyl 2,2′-pyridyl-quinoline-4-carboxylate (**pqcame**) and dimethyl 2,2′-biquinoline-4,4′-dicarboxylate (**biqcame**) were performed according to the literature [43,44] and are presented in Scheme 1.

Selected NMR data (^1^H NMR and/or ^13^C spectra) of **8-Mepq**, **6′-Mepq**, **8,6′-Me_2_pq**, **4,6′-Mepq** and **pq**, recorded in CDCl_3_ are included in the Supplementary Materials (Figures S1–S12) and are in accord with the structures shown in Scheme 1. Assignment was based on a combination of ^1^H–^1^H COSY, ^1^H–^13^C HSQC and ^1^H–^13^C HMBC methods. Thus, for example, the ^1^H NMR spectrum of **4,6′-Me_2_pq** (Figure S7) displays eight resonance signals typical for the aromatic ring protons, while the two singlet resonance signals at *δ* 2.70 and *δ* 2.80 ppm, respectively, are attributed to the methyl group protons.

Additional proof of identity is provided by the high-resolution electrospray ionization mass spectrometry (ESI-HRMS) data, of the corresponding compounds **8-Mepq**, **6′-Mepq** and **8,6′-Me_2_pq** (Figures S13–S15). For **8**-**Mepq** and **6′**-**Mepq**, the peak at *m*/*z* 221.1095 is typical for the protonated [M + H]^+^ ion, while that at *m*/*z* 243.0888 (**8**-**Mepq**) can be attributed to the sodium adduct [M + Na]^+^. For both compounds, the low intensity molecular ion detected at *m*/*z* 463.1966 and 463.1892 can be assigned to the sodium adduct of the dimer [2M + Na]^+^. Similarly, the mass spectrum of **8,6′-Me_2_pq** displays a basic ion at *m*/*z* 235.1277 due to the protonated ligand [M + H]^+^, accompanied by peaks at *m*/*z* 257.1056 and *m*/*z* 273.0791, for the sodium [M + Na]^+^ and potassium adducts [M + K]^+^, respectively. The low intensity ion at *m*/*z* 491.2214 is attributable to the sodium adduct of the dimer [2M + Na]^+^.

**Figure 1:**
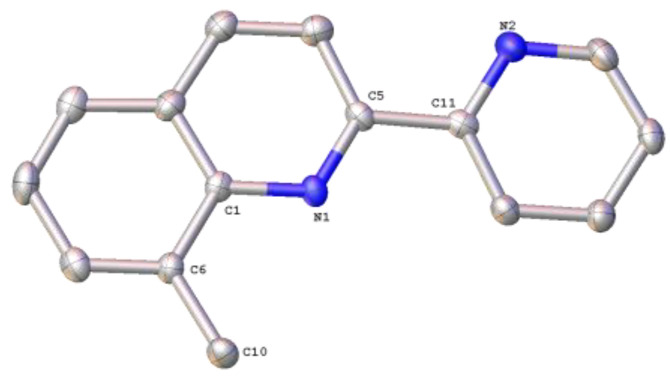
Molecular structure of **8-Mepq**; ellipsoids are plotted at the 50% probability level. H atoms are omitted. Selected bond lengths (Å) and angles (°): N(1)–C(1) 1.376(5); N(1′)–C(1′) 1.365(5); N(2)–C(11) 1.346(6); N(1)–C(5) 1.321(5); N(1′)–C(5′) 1.326(5); N(2)–C(11) 1.346(6); C(1)–C(2) 1.416(6); C(1′)–C(2′) 1.423(6); C(5)–N(1)–C(1) 118.1(3); C(11)–N(2)–C(15) 117.3(4); C(5′)– N(1′)–C(1′) 118(3).

**Figure 2:**
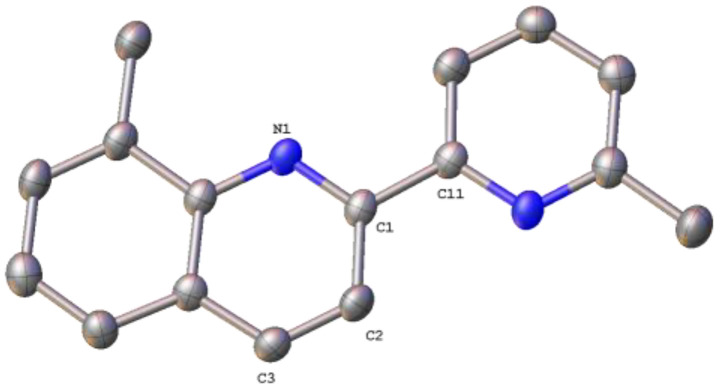
Molecular structure of **8,6′-Me_2_pq**; ellipsoids are plotted at the 50% probability level. H atoms are omitted. Selected bond lengths (Å) and angles (°): N(1)–C(1) 1.3718(15); N(1)–C(5) 1.372(5); N(2)–C(11) 1.347(5); N(2)–C(15) 1.346(5); C(1)–C(2) 1.415(5); C(5)–N(1)–C(1) 118.6(3); C(11)–N(2)–C(15) 117.7(3); N(1)–C(5)–C(4) 122.5(3).

**Figure 3:**
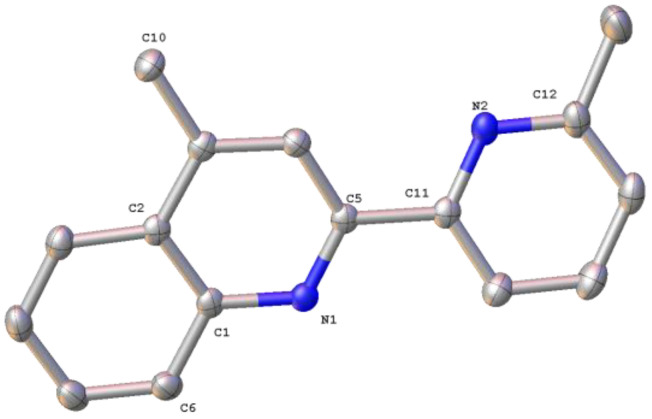
Molecular structure of **4,6′-Me_2_pq**; ellipsoids are plotted at the 50% probability level. H atoms are omitted. Selected bond lengths (Å) and angles (°): N(1)–C(1) 1.3718(15); N(1)–C(5) 1.3257(16); N(2)–C(11) 1.3476(16); N(2)–C(12) 1.3452(16); C(1)–C(2) 1.4203(17); C(5)–N(1)–C(1) 117.27(10); C(11)–N(2)–C(12) 118.25(11); N(1)–C(5)–C(4) 123.26(10).

The solid-state structures of **8-Mepq, 8,6′-Me_2_pqca** and **4,6′-Me_2_pq** have been unambiguously determined by single-crystal diffraction studies. Colorless single crystals, suitable for X-ray diffraction, were grown upon slow evaporation of a MeOH/H_2_O solution of the compounds at ambient temperature. Notably, a CSD search performed (5/2025), in the crystallographic data center, revealed that the structures of these small molecules had not been reported previously. The molecular structures of these compounds are depicted in Figure 1, Figure 2 and Figure 3.

The three molecules **8-Mepq**, **4**,**6′-Me_2_pq** and **8,6′-Me_2_pq**, crystallize in the orthorhombic crystallographic system, space groups *P*n2_1_a, *P*2_1_2_1_2_1_ and *P*bca, respectively. As expected, in the bipyridine domain, the two nitrogen atoms are in the *trans*-configuration [17,20]. In **8-Mepq**, the pyridine ring close to quinoline, deviates from planarity since the angle between the least-squares planes of the pyridyl and quinoline rings, containing atoms N2 and N1, is 13.69°. For **4**,**6′-Me_2_pq**, this angle drops to 9.11°, while for **8,6′-Me_2_pq**, that is a planar molecule, this angle is 1.74°.

In the asymmetric unit of **8-Mepq**, two crystallographically independent molecules are present, which are arranged in a head-to-tail fashion and their bonding parameters differ slightly. The packing in the crystal is such that molecules of **8-Mepq** are stabilized by classical intermolecular hydrogen bonding interactions between the nitrogen atom N1 of quinoline moiety and the hydrogen atom H13 of a second molecule (distance of C13–H13⋯N1**′** = 3.440(4) Å, bond angle = 164.15(4)°; C13**′**–H13**′**⋯N1 = 3.561(5) Å, bond angle = 161.84(5)°) as shown in Figure S16 of the Supplementary Materials. For **4,6′**-**Me_2_pq**, the preference of the *trans*-configuration can be attributed to the intramolecular hydrogen bonding interactions of N1 and N4 to the adjacent H15 and H4, respectively (distance of N1⋯H15–C15 = 2.514(5) Å, bond angle = 98.21(5)°and N2⋯H4–C4 = 2.475(5) Å, bond angle = 100.09(5)°). In the unit cell, stabilization is provided by weak intermolecular non-classical hydrogen bonding interactions (distance of (C10C–H10C)⋯centroid (N1–C1–C2) is 3.529(4) Å, Figure S17) along with typical intermolecular hydrogen bonding interactions via its N2 and N1 atoms, respectively (distance of N2⋯H6–C6 = 2.932(4) Å, bond angle of 131.83°; distance of N1⋯H14–C14 = 2.581(4) Å, bond angle of 143.94(4)°). Finally, the crystal of **8,6′**-**Me_2_pq** is stabilized by intramolecular non-classical C–H⋯π contacts, (distance of (C13–H13)⋯centroid (C4–C9) = 3.612(6) Å; distance of (C10C–H10C)⋯centroid (N2–C11–C15) = 3.604(6) Å) and by non-classical intermolecular interactions (C3–H3**⋯**N2 = 2.954(6) Å; bond angle = 145.19(6)°, Figure S18).

### 2.2. Synthesis and Characterization of the Ruthenium(II) Complexes ***1***–***8***

**Scheme 2:**
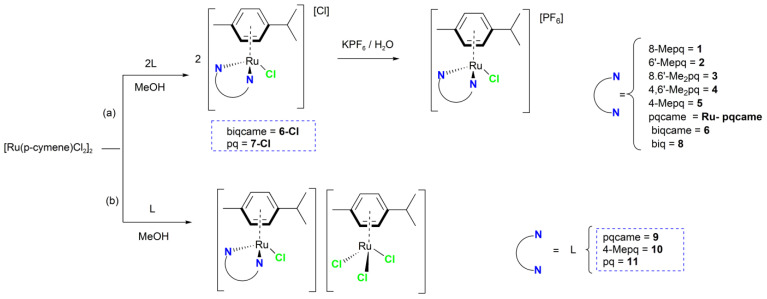
General reaction scheme for the synthesis of the ruthenium(II) organometallic complexes **1**–**8** (**a**) and **9**–**11** (**b**), described in this work.

All the organic ligands reported herein were synthesized aiming to prepare new and efficient ruthenium(II)-based transfer hydrogenation catalysts. Interestingly, a search of the literature revealed that the coordination chemistry of **8-Mepq**, **6′-Mepq, 8,6′-Me_2_pq** and **4,6′-Me_2_pq** is currently unknown, while for **biqcame**, only a silver(I) complex has been reported [44]. These findings have prompted us to synthesize new organometallic ruthenium(II) complexes with the aforementioned ligands. The reaction of the ruthenium(II) dinuclear complex [Ru(η^6^-*p*-cymene)(μ-Cl)Cl]_2_, with two molar equivalents (slight excess) of the appropriate ligand L was performed in dry methanol under an argon atmosphere. Subsequent treatment of the non-isolated chlorido intermediates, with a saturated aqueous KPF_6_ solution, afforded the η^6^-arene ruthenium(II) complexes of the general type [Ru(η^6^-p-cymene)(L)Cl][PF_6_] (L = 8-Mepq (**1**), 6′-Mepq (**2**), 8,6′-Me_2_pq (**3**), 4,6′-Me_2_pq (**4**), 4-Mepq (**5**), biqcame (**6**), biq (**8**)) as bright yellow (orange for **6**) colored solids in high yield. As representative chlorido analogs of the series, complexes **6**-**Cl** and **7-Cl**, incorporating the biqcame and pq ligands**,** were isolated as hygroscopic solids and spectroscopically characterized. In addition, the synthesis of **8** was performed with a slight modification of the published procedure [35], using KPF_6_ instead of NH_4_PF_6_, leading in a higher synthetic yield (85% instead of 71%). All complexes are air stable solids that dissolve in methanol, ethanol, acetone and dimethylsulfoxide, while remain insoluble in dichloromethane, chloroform and in water. The synthetic procedure and reaction conditions are depicted in Scheme 2a.

The new complexes were characterized by FT-IR, elemental analysis, multinuclear NMR spectroscopy and UV-Vis spectroscopy. The infrared spectra (Figures S19–S25) recorded in the region of 4000-400 cm^−1^ are very similar, as expected for compounds with similar molecular structures. The spectra, exception is for **6**-**Cl** and **7-Cl**, are dominated by the very strong typical bands at ∼840 cm^−1^ and 557 cm^−1^ that can be assigned to the *ν*_3_(P–F) and *ν*_4_(P–F) vibration modes of the [PF_6_]^−^ anion [45]. Elemental analyses data of all complexes corroborate with the calculated composition of the proposed structures. These were performed with the powder like solids obtained during their synthesis.

The ^1^H and ^13^C NMR spectra of all complexes in Me_2_CO-d_6_ (in CDCl_3_ for **6-Cl**) were assigned by 2D techniques (Figures S26–S47) and are in accord with the proposed structures depicted in Scheme 2a. The ^1^H NMR spectra of **1** and **5** are quite similar, displaying the expected resonance signals attributed to the aromatic ring protons of **8-Mepq** and **4-Mepq** ligands. These are shifted upfield compared to the resonance signals of the free ligands, indicating further coordination to the ruthenium(II) center. The *p*-cymene ligand displays four separate resonance signals (doublets) in between *δ* 6.16 and 5.49 ppm, which are shifted upfield compared to the dinuclear ruthenium(II) precursor. In addition, the two doublets of doublets at *δ* 1.02, 0.90 (**1**) and *δ* 0.95, 0.91 ppm (**5**) can be assigned to the methyl protons of the isopropyl group. The resonance signals reported above are typical for non-symmetric ruthenium *p*-cymene complexes [46]. In the aliphatic region, characteristics are the high field signals at *δ* 3.31 (**8-Mepq**) and *δ* 3.00 ppm (**4-Mepq**), for the methyl groups of the quinoline moieties, respectively. For **6**-**Cl** (CDCl_3_) and **8** (Me_2_CO-d_6_) however, two doublet resonance signals are observed for the *p*-cymene ligand (*δ* 5.98/5.81 (**6**-**Cl)** and *δ* 5.98/5.87 (**8**)), and one doublet resonance for the methyl protons of the isopropyl groups (*δ* 1.11 (**6**-**Cl**) and 0.89 ppm (**8**)), due to the highly symmetric **biqcame** and **biq** ligands. The ^1^H NMR data of **8** agree to those reported previously for the analogous PF_6_^−^ salt [35].

The electronic absorption spectra of Me_2_CO solutions of the ruthenium(II) complexes **1**-**8** exhibit typical high energy absorption bands in the region of ∼260–350 nm that can be attributed to ligand-centered π–π* transitions and are included in Figures S48–S55. All complexes display a main and broad MLCT (metal to ligand charge transfer) absorption band centered between 476 and 412 nm, with ε values ranging from 1300 to 3800 dm^3^ mol^−1^ cm^−1^.

### 2.3. Structural Characterization of Complexes ***1***–***4*** and ***6***

**Figure 4:**
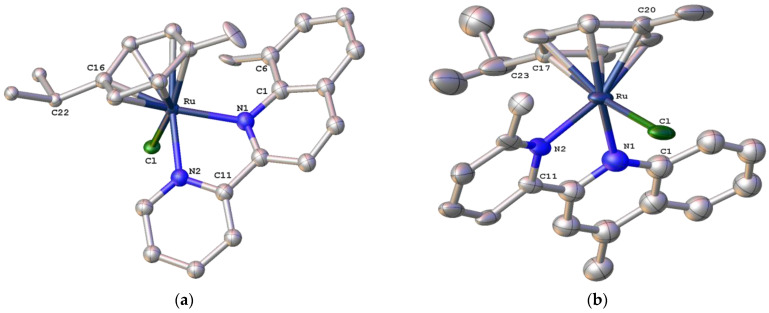
(**a**) Molecular structure of the complex cation of **1**; (**b**) Molecular structure of the complex cation of **4**. Hydrogen atoms and the PF_6_^−^ anions are omitted for clarity. The ellipsoids were plotted at the 50% probability level.

**Figure 5:**
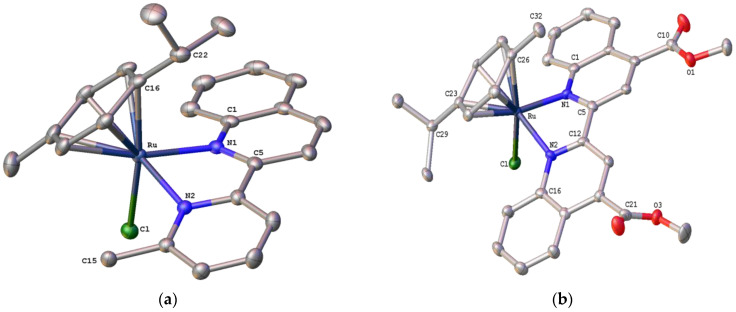
(**a**) Molecular structure of the complex cation of **2**; (**b**) Molecular structure of the complex cation of **6**. Hydrogen atoms and the PF_6_^−^ anions are omitted for clarity. The ellipsoids were plotted at the 50% probability level.

The molecular structures of complexes **1**–**4**, **6** and **9** in the solid-state have been unambiguously determined by single-crystal X-ray diffraction, while suitable single crystals were obtained by the slow diffusion of dry pentane into an acetone solution of the compound. All complexes adopt a pseudo-octahedral geometry also known as three-legged piano stool. The ruthenium(II) center is surrounded by a chlorine atom, two N atoms of the appropriate chelating ligand, while the remaining basal sites are occupied by the η^6^-*p*-cymene ligand. Notably, a Cambridge Crystallographic Database search (5/2025) revealed that there are no reports about metal complexes comprising the bidentate ligands reported herein. The solid-state structures of the complex cations of **1** and **4** are depicted in Figure 4 and Figure 5, providing evidence about the nature of the molecules in the crystal.

Complexes **1** and **4** crystallize as clear orange block and plank crystals in orthorhombic and monoclinic *P*bca and *C*2/c space groups. For both complexes, the Ru–Cl (~2.4 Å) and Ru–N (N1 and N2) bond lengths (2.149(15) Å, 2.063(14) Å (**1**) and 2.102(12) Å, 2.121 (11) Å (**4**)) are very close to those of similar complexes reported in the literature [26,47,48].

The crystal packing of **1** involves typical intramolecular π⋯π stacking interactions between ring centroids (C1–N1–C5) and (C16–C21) of adjacent molecules at a separation of 3.742(5) Å. The crystal is stabilized further by non-classical hydrogen bonding interactions (C3–H3⋯Cl = 2.834(5) Å, bond angle = 177.74(5)°) and C24S–H24D⋯Cl = 2.751(5) Å, bond angle = 140.80(5)°, Figure S56). Coordinated ligands **8-Mepq** and **4,6′-Me_2_pq** in **1** and **4**, deviate from planarity as the angle between the planes of the pyridine ring and that of quinoline ring is 9.07(5)° (**1**) and 7.81(5)° (**4**), respectively. In the unit cell of **4**, the structure is reinforced by non-classical intermolecular hydrogen bonding interactions (C26A–H26A⋯Cl = 2.886(4) Å; bond angle = 115.14(4)°) and intramolecular hydrogen bonding interactions C12–H12⋯Cl = 3.074(5) Å, bond angle = 152.70(5)°; C13–H13⋯Cl = 3.556(5) Å, bond angle = 89.44(5)°, Figure S57).

The mononuclear cationic complex **2** (Figure 4) with a methyl group at the 6′ position, crystallizes in the monoclinic space group *P*2_1/c_. In this chlorido analog, the Ru–Cl bond length of approximately 2.4 Å and the Ru–N1 and Ru–N2 bond lengths of 2.1129(14) Å and 2.1054(15) Å, respectively, are comparable to those of other similar ruthenium(II) complexes [48]. In addition, the N1–Ru–Cl and N2–Ru–Cl bond angles are 86.55(4)° and 85.87(4)°, respectively. The bond distance between ruthenium atom and *p*-cymene ring centroid (C16–C21) is 1.694 Å, while the planes defined by the pyridine (N1–C1–C5) and quinoline rings (C1–C2–C6–C9), deviate from planarity by 13.62(14)°. The crystal network is reinforced by characteristic non-classical C–H⋯π intramolecular interactions (distance of C24B–H24B⋯centroid (C18–C21 of cymene) = 3.433(4) Å) and by typical intramolecular contacts between the hydrogen atom H24A of the isopropyl group and the quinoline ring centroid from an adjacent molecule (distance of C24–H24A⋯centroid (C1–C5–N1) = 2.784(4) Å). Moreover, stability is provided by intermolecular hydrogen bonding interactions between molecules of **2** in the crystal (C25–H25A⋯Cl = 2.769(3) Å, bond angle = 109.68(3)°) along with intramolecular contacts including coordinated Cl atom and H15C for methyl group in pyridine (C15–H15C⋯Cl = 2.878(4) Å, bond angle = 138.73(4)°). Within the crystal of **2**, the hydrogen atom H6 from the quinoline ring, participates in an intermolecular hydrogen bonding interaction with the Cl atom coordinated to ruthenium (C6–H6⋯Cl = 3.129(4) Å, bond angle = 119.33(4)°, Figure S58).

Clear red plates of **6** were crystallized in a monoclinic crystal system and P2_1/n_ space group. The structural features of complex **6** are quite similar to those of **1**, **2** and **4**. Within the cell, the crystal is stabilized by intramolecular non-classical hydrogen bonding interactions between O4 and H32 from the isopropyl group of an adjacent molecule (O4(A)…H32(B)-C32(B) = 3.125(4) Å, bond angle 133.30(4)°). Also, stability is provided by non-classical C–H⋯π intramolecular interactions (distance of C11C–H11C⋯centroid (C1–C9) = 3.531(4) Å, Figure S59)

**Figure 6:**
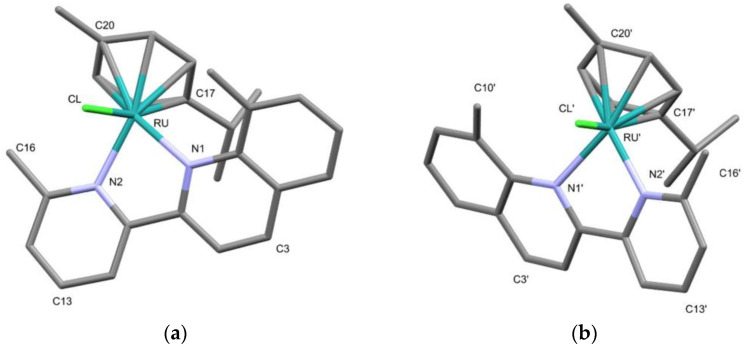
(**a**) Molecular structure of the complex cation [Ru(η^6^-*p*-cymene)(8,6′-Me_2_pq)Cl]^+^ (Ru-**3**); (**b**) Molecular structure of the complex cation [Ru′(η^6^-*p*-cymene)(8,6′-Me_2_pq)Cl]^+^ (Ru′-**3**). Hydrogen atoms and the PF_6_^−^ anions are omitted for clarity. The ellipsoids were plotted at the 50% probability level.

Upon crystallization of complex **3**, dark red single crystals were afforded which contained two independent molecules of [Ru(η^6^-*p*-cymene)(8,6′-Me_2_pq)Cl][PF_6_] (Ru-**3**) and [Ru′(η^6^-*p*-cymene)(8,6′-Me_2_pq)Cl][PF_6_] (Ru′-**3**) in the unit cell, in the ratio of 1:1 (Figure 6).

**Figure 7:**
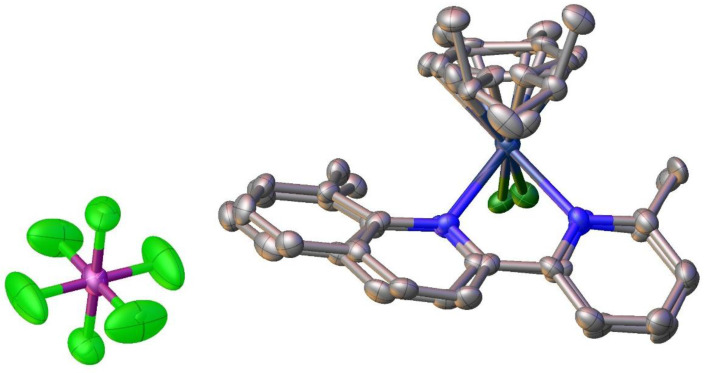
A superposition of both independent molecules of **3** in the unit cell. Hydrogen atoms are omitted for clarity. The ellipsoids were plotted at the 50% probability level.

This was the result of a slight rotation of the arene ring, which renders both molecules different. For clarification, a superposition of both independent molecules is shown in Figure 7.

In both molecules, the ruthenium metal center adopts a typical pseudo-octahedral coordination geometry, as shown by the N(1)–Ru–N(2), N(1)′–Ru′–N(2)′ bond angles of 78.2(3)° and 77.60(2)°, and by the N(1)–Ru–Cl, N(1)′–Ru–Cl′ bond angles of 93.46(2)° and 94.36(2)°, respectively. These structural characteristics are common for η^6^-arene ruthenium complexes [48]. The bonding parameters of both independent molecules differ slightly. The ruthenium–centroid (C17–C22) and Ru–N1 bond lengths of Ru-**3** are slightly elongated compared to those of Ru′-**3**, while the Ru–N2 bond length slightly decreased, probably reflecting the effect of the slight rotation of the arene ring. Moreover, the relevant N1–Ru–Cl (93.46(2)°) and N2–Ru–Cl (82.90(2)°) bond angles of Ru-**3**, differ slightly from those of Ru′*-***3** ((94.36(2)° and (83.37(2)°). The Ru–Cl bond remains practically unaltered at 2.390 Å. In both molecules, the aromatic rings comprising the quinoline moieties deviate from planarity by 8.40° and 6.41°, respectively. In the unit cell, pairs of molecules of **3** are stabilized by intermolecular π–π stacking interactions including the ring centroids (N2–C11–C15, pyridine) and (C6–C9 quinoline) and the ring centroids (N1–C1–C5, quinoline) and (C11–C15, pyridine) of adjacent molecules, at a separation of 3.980 (4) Å and 3.698(4)Å (Figure S60).

Improved crystallographic data of the known crystal structure of complex **8**, are included in the Supplementary Materials (Figure S61 and Table S1) [35]. The solid-state structure of **8** reveals a typical pseudo-octahedral geometry considering that the η^6^-*p*-cymene ligand occupies three facial coordinated positions. The structural features of **8** reported herein are slightly improved, as shown, for example, from the bond angles N1–Ru–N2 = 76.49(7)° (76.6(4)° published), N1–Ru–Cl = 86.52(5)° (87.8(3)° published) and N2–Ru–Cl = 87.59(5)° (86.4(3)° published), respectively. In the unit cell, the crystal is stabilized by intramolecular non-classical C–H⋯π intramolecular interactions (distance of C27A–H27A⋯centroid (C1–C9) = 3.961(7) Å; distance of C27C–H27C⋯centroid (C1–C9) = 4.676 (7) Å, Figure S62).

### 2.4. Synthesis and Characterization of the Ruthenium(II) Complexes ***9***–***11***

The cationic ruthenium(II) complexes **9**–**11** (Scheme 2b) were prepared according to the procedure followed for complexes **1**–**8**, using one mol equivalent of the relevant ligand. As a result, the [Ru(η^6^-*p*-cymene)(L)Cl][Ru(η^6^-*p*-cymene)Cl_3_] (L = pqcame (**9**), 4-Mepq (**10**), pq (**11**)) analogs, comprising the [Ru(η^6^-*p*-cymene)Cl_3_]^−^ anion, were isolated as orange solids, in excellent yields (> 90%). They are air stable water-soluble solids, that dissolve in common organic solvents, while they are not soluble in acetone, diethyl ether and pentane. Although there are recent reports of ruthenium(II) complexes consisting of this counter ion [49,50], none of them refer to substituted pyridine–quinoline-based ligands.

The FT-IR spectra of **9**–**11** (Figures S63–S65) are very similar. The spectra are dominated by the intense ν(C–H) aromatic and aliphatic stretching vibration bands along with those of ν(C–C) stretching vibration modes. The intensity of these vibration modes in **9**–**11** is significantly higher compared to the relevant congener complexes **3**, **5** and **8,** which do not contain the [Ru(η^6^-*p*-cymene)Cl_3_]^−^ counter anion.

The ^1^H and ^13^C NMR spectra of **9**–**11** in CDCl_3_, were assigned by two-dimensional routine techniques (Figures S66–S74). Interestingly, the -CH resonance signal from the -C*H*(CH_3_)_2_ group, corresponding to the [Ru(η^6^-*p*-cymene)(pqcame)Cl]^+^ cation of **9**, is split in two resonance signals (septets) at δ 3.20 (CH_A_) and 2.94 (CH_B_) ppm, in a ratio of 60:40. Accordingly, the two doublet resonances at *δ* 1.38 (CH_3A_) and 1.30 (CH_3B_) ppm are attributed to the methyl group protons of -CH(C*H*_3_)_2_. Moreover, the two singlets at δ 2.31 (CH_3A_-*p*-cym) and 2.18 (CH_3B_-*p*-cym) ppm (integration for 3H), were assigned to the methyl group hydrogens of *p*-cymene from the complex cation of **9**. By analogy, the septet at δ 2.40 ppm is typical for the -CH’ proton of -C*H*’(CH_3_′)_2_ group corresponding to the [Ru(η^6^-*p*-cymene)Cl_3_]^−^ counter anion. The doublet resonances at δ 0.96 and 0.91 ppm can be attributed to the methyl group protons of -CH’(C*H*_3_′)_2_. Finally, the broad singlet at δ 2.18 ppm overlapping with the resonance signal of CH_3B_-p-cym, can be assigned to the C*H*_3_′-*p*-cym protons. Analogous behavior has been reported for complexes **10** and **11,** with a relevant ratio of approximately 55:45, corresponding to the -CH resonance signals from the -C*H*(CH_3_)_2_ group, which are split. The absorption spectra of **9**–**11** recorded in CHCl_3_ display a relatively broad absorption band in between 415 and 450 nm assigned to metal-to-ligand charge transfer (MLCT) transitions (Figures S75, S77 and S79). In water, less intense MLCT transitions were observed, but within the same range (Figures S76 and S78).

### 2.5. Structural Characterization of Complex [Ru(η^6^-p-cymene)(pqcame)Cl][Ru(p-cymene)Cl_3_] CH_2_Cl_2_ (***9***)

**Figure 8:**
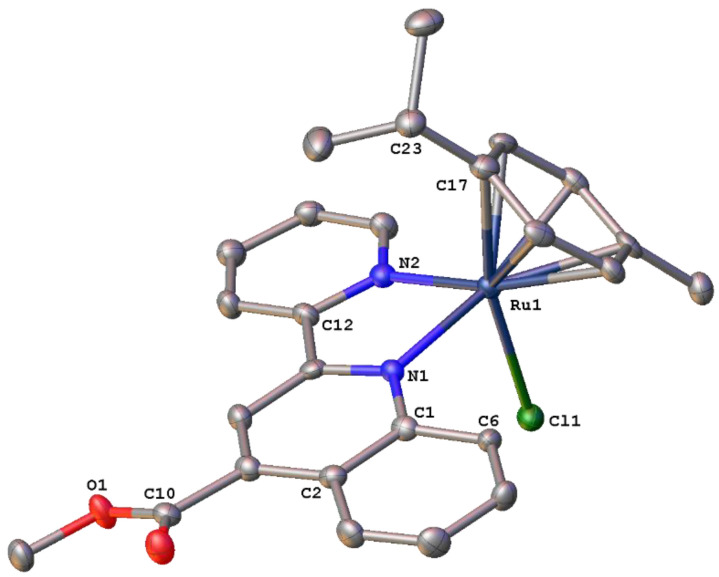
Molecular structure of the complex cation of **9**. Hydrogen atoms, solvent molecules and the [Ru(p-cymene)Cl_3_]^−^ complex anion are omitted for clarity. The ellipsoids were plotted at the 50% probability level.

Additional information about the structure of **9** is provided by single-crystal X-ray crystallography performed on clear red plank crystals, which crystallized in the monoclinic space group *P*2_1/n_. Suitable crystals of **9** CH_2_Cl_2_ were obtained upon the slow diffusion of pentane into a CH_2_Cl_2_ solution of the complex (Figure 8). In the unit cell, each [Ru(η^6^-*p*-cymene)(pqcame)Cl]^+^ complex cation is surrounded by a [Ru(η^6^-*p*-cymene)Cl_3_]^−^ complex ion, both displaying a three-legged piano stool geometry, including also a dichloromethane molecule. The bond distance between the ruthenium(II) center and the *p*-cymene ring centroid (C17–C22) of [Ru(η^6^-*p*-cymene)(pqcame)Cl]^+^ is 1.689(4) Å. All other structural features, (Ru–N, Ru–Cl bond distances and relevant bond angles) comply well with those reported for the analogous [Ru(η^6^-*p*-cymene)(pqcame)Cl](PF_6_) complex [33], and to those of complexes **1**–**4** and **6**, reported herein. Within the [Ru(η^6^-*p*-cymene)Cl_3_]^−^ counter ion, the Ru–Cl bond distances are in the range of 2.429–2.436 Å. Moreover, the bond angles Cl(2)–Ru(2)–Cl(3) = 86.95(4)°, Cl(2)–Ru(2)–Cl(4) = 88.10(5)° and Cl(3)–Ru(2)–Cl(4) = 87.91(4)°, respectively, are in agreement with analogous complexes incorporating this complex ion [49]. In the crystal, stabilization is provided by intramolecular non-classical hydrogen bonds (C25A–H25A⋯Cl2 = 3.817(4) Å, bond angle = 97.87(4)°). Additional short contacts include typical intermolecular C–H⋯π interactions between the methyl group of the isopropyl moiety and the pyridine and quinoline rings, respectively (C24A–H24A…centroid (C12–C16–N2) = 3.263(4) Å and C24C–H24C…centroid (C1–C5–N1) = 3.351(4) Å, Figure S80).

### 2.6. Catalytic Transfer Hydrogenation Studies

**Scheme 3:**
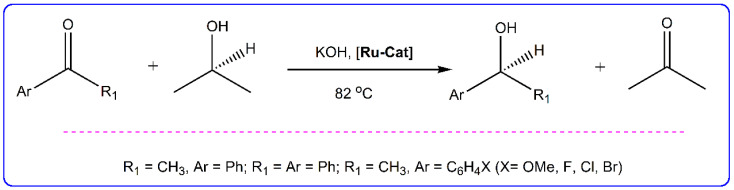
Catalytic transfer hydrogenation of benzophenone and acetophenone derivatives by catalysts **1**–**11**, [Ru-Cat].

Complexes **1**–**11** have been prepared aiming to evaluate their catalytic performance in the transfer hydrogenation reactions of various aromatic ketones (Scheme 3).

All experiments were performed at 82 °C using 2-propanol as a hydrogen donor, in the presence of KOH as a base (KO*i*Pr), according to well-defined protocols [30,51]. From the initial experiments conducted, the optimum conditions were verified by using 0.25 mol% of the ruthenium catalyst and 10 mol% of KO*i*Pr, leading to a substrate/catalyst/base molar ratio of approximately 400:1:40. The percent conversion of the substrates examined, to the corresponding alcohols, was monitored over time by ^1^H NMR spectroscopy. For acetophenone and their substituted derivatives, the characteristic singlet resonance due to the methyl group hydrogens was used as a probe to follow the reaction. For benzophenone reduction, the -CH resonance signal of the desired alcohol [Ph_2_CH(OH)] was detected. Finally, the formation of the desired alcohol, 1-(phenyl)-ethanol, benzhydrol, etc., as the final product, was identified by comparison with the ^1^H NMR data of authentic samples. The catalytic experiments were performed twice to ensure reproducibility of the results. Acetophenone was used as a model substrate and the results of the preliminary screening are summarized in Table 1.

All complexes significantly catalyze the transfer hydrogenation of acetophenone in the presence of a base. At ambient temperature, conversions < 10% were observed while the presence of the base proved crucial for the catalytic experiment. Parameters such as (***i***) the presence of electron donating groups (methyl) either in the pyridine or quinoline ring and/or in both rings (complexes **1**–**5**, **11**), (***ii***) the presence of electron withdrawing groups (COOMe, **6** and **Ru-pqcame**) and (***iii***) the effect of the different counter ion used ([PF_6_]^−^, **1**–**8** vs. [Ru(η^6^-*p*-cymene)Cl_3_]^−^, **9**–**11**), were examined.

**Figure 9:**
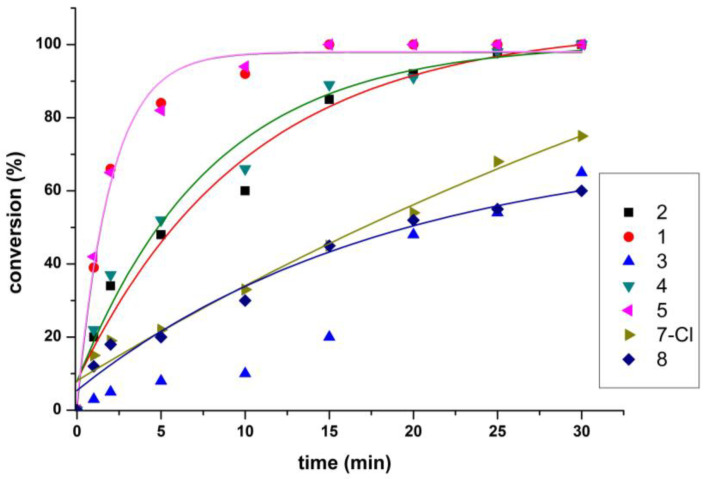
Time dependence of transfer hydrogenation of acetophenone by catalysts **1**–**5**, **7-Cl** and **8**.

From Table 1, it becomes evident that the presence of electron donating groups (in certain positions) significantly improves the catalytic properties of complexes **1**–**5**. An exception stands for **3**, probably due to steric reasons, as the two methyl groups of the **8,6′-Me_2_pq** ligand are in close proximity to the ruthenium(II) center of the catalyst (entry 5). It seems that an incubation period of ~15 min is required to activate this catalyst. Within the series, catalysts **1** and **5** bearing methyl groups at positions 8 and 4 of the quinoline moiety exhibit the best activity. The reduction in acetophenone proceeded smoothly to afford quantitatively 1-(phenyl)-ethanol within ~15 min (entries 1 and 8). The time dependence of transfer hydrogenation of acetophenone by catalysts **1**–**5**, **7-Cl** and **8** is shown in Figure 9.

From a closer view of Figure 9, it is clearly seen that **1** and **5** reduce >80% of acetophenone within the first 5 min, while conversion reaches almost 92%, practically quantitative, after 10 min of reaction. This is in contrast with the reduced catalytic activity of an analogous complex bearing a 2-(5,6-dimethyl-1H-benzimidazol-2-yl)quinoline ligand with methyl groups at the 5,6-positions [28]. For complexes **2** and **4**, with methyl groups at positions 6′ and 4,6′, respectively, a two-fold decrease in activity was observed, since both complexes require approximately 30 min to reduce acetophenone (entries 3 and 7). Catalysts **1**, **2**, **4** and **5** proved more efficient compared to **7-Cl** and **8**. The latter containing the non-substituted pq (**7-Cl**) and biq (**8**) ligands need almost 60 min to exhibit the highest activity (entries 12 and 13). Notably, for complex **3** comprising the rather bulky 8,6′-Me_2_pq ligand with two methyl groups close to the ruthenium center, an induction period of ~15 min is required (entry 4). However, within the next 5 min of reaction, the conversion rate increases dramatically from ~20% to almost 50%, reaching a quantitative yield, within the limits of instrumental detection, after approximately 55 min. On the other hand, the catalytic potency of the methyl ester analogs **Ru-pqcame** and **6** (electron withdrawing groups) is significantly reduced, since the time required for the hydrogenation of acetophenone is ~180 min (entries 10 and 11). For **Ru-pqcame**, within the first 60 min, hydrogenation reaches a 38% conversion. This result comes to an agreement with that reported for the neutral ruthenium(II) triphenylphosphine complex cis-[RuCl_2_(PPh_3_)_2_(pqcame)] [31,32].

The higher catalytic performance of **1** and **5** is reflected also by the final TOFs of 1600 h^−1^ achieved. All other complexes display lower TOF values ranging from 360 to 1440 h^−1^. To this end, we must report that the catalytic activity of complexes **1**–**5** is comparable to that of other half-sandwich ruthenium(II) complexes bearing 2,2′-bipyridine and 4,4′-dimethy-2,2′-bipyridine, 2-amino-pyridine and 2,2′-quinoline-benzimidazole ligands [20,24]. Interestingly, their activities are higher than those of the relevant ruthenium(II) arene complexes with N-substituted 3,4-dihydroquinazoline ligands, that need 60 min for conversion of acetophenone [52]. In addition, complexes **1** and **5** exhibit higher conversions in comparison with the [RuCl_2_(benzene)(PAr_3_)] complexes (Ar = *p*-methoxyphenyl, triphenyl, *p*-trifluoromethylphenyl) [53], and the cationic complex [Ru(ImH)_2_(*p*-cym)Cl]Cl; ImH = 1H-Imidazole (33% for acetophenone conversion) [27]. Notably the potency of both catalysts is significantly higher than that reported for the arene ruthenium(II) derivatives using different oximes (11*H*-indeno [1,2-*b*]quinoxaline-11-one oximes and tryptanthrin-6-oxime) [26]. On the other hand, catalysts **1** and **5** are less efficient compared to ruthenium(II) complexes that contain bidentate ligands with P,N atoms [17,20].

For complexes **9**–**11**, bearing the same ligands as complexes **Ru-pqcame**, **5** and **7-Cl**, respectively, interesting transfer hydrogenation results were obtained. Within the series, complex **11** is the most potent, converting acetophenone by 90% in 15 min (entry 16). In contrast, the structurally similar complex **5** exhibits quantitative conversion, within the same period. Presumably, the presence of the less constrained [PF_6_]^−^ ion, as opposed to the sterically crowded [Ru(η^6^-*p*-cymene)Cl_3_]^−^ analog of **11**, may be responsible for the improved catalytic activity of **5**. In line with that, (entry 17) complex **7-Cl** is more active (95%) than **11** (90%). This also may be the case for complex **Ru-pqcame** when compared to complex **9** (entries 15 and 10), possibly highlighting the role of the sterically crowded [Ru(η^6^-*p*-cymene)Cl_3_]^−^ complex anion in **9**. For both catalyst precursors, conversion of acetophenone transfer hydrogenation versus reaction time was compared during the first hour, and the results can be seen in the graph of Figure S81. For the catalyst with the [PF_6_]^−^ anion, conversion of the substrate is higher than its [Ru(η^6^-*p*-cymene)Cl_3_]^−^ analog. The TOFs reported for **Ru-pqcame** and **9** are 120 h^−1^, somehow lower compared to those of **7-Cl** (380 h^−1^) and **11** (360 h^−1^) and significantly lower than those of **10** (1440 h^−1^) and **5** (1600 h^−1^), respectively.

After an initial screening with acetophenone, the encouraging results obtained by catalysts **1** and **5** prompted us to study transfer hydrogenation reactions with *p*-substituted derivatives of acetophenone along with benzophenone (Table 2). All complexes showed very good to excellent catalytic potencies. In general, the tendency observed is similar to that reported for the conversion of acetophenone. Notably, 4-fluoro-acetophenone and 4-chloro-acetophenone substrates, with electron withdrawing groups at the *p*-position, were quantitatively converted by **1** and **5** within ~15 min. For complexes **3**, **6**, **Ru-pqcame** and **11,** however, the conversion rate is slightly decreased by approximately 5 to 10%. Interestingly, the rate of 4-bromo-acetophenone has decreased (conversion range of 55–73%) compared to the relevant analogs substituted by chlorine and fluorine in the *para* position. For complex **6**, however, an 88% conversion was achieved over 180 min. This may be due to the higher mesomeric effect of –Br group that has a result on the reduced electron density of the carbonyl bond of the substrate [31].

In comparison, several catalysts like [RuCp(PN)Br] (PN = N,N-dimethyl-2-diphenylphosphine-ethylamine) [54], [RuCl_2_(dcype)(bipy)] (dcype = 1,2-di-(dicyclehexaphospine)ethane, bipy = 2,2′-bipyridine) [55] and [Ru(η^6^-*p*-cymene)(L)Cl]^+^ (L = 4-((Ε)-(4-ethylphenyl)diazenyl)-2((E)-(phenylimino)methyl)phenol) [56], display analogous behavior. Substitution in the *para* position by a methoxy group decreases the rate of reduction, ranging approximately in between 70 and 96%, which can be attributed to the electron donating properties of the *p*-substituted methoxy group.

Finally, we carried out transfer hydrogenation of benzophenone using these catalysts. As seen from Table 2 (entries 49–53), complexes **1**–**5** exhibited considerable efficiencies ranging from 80 to 99%, though the reduction of benzophenone to benzhydrol is about 2 to 4 times less efficient compared to acetophenone. It should be noted that the cationic ruthenium(II) catalyst **5**, is less efficient (60 min) in the transfer hydrogenation of benzophenone, compared to the neutral ruthenium(II) complex cis-[RuCl_2_(PPh_3_)_2_(4-Mepq)]. The latter reached a 96% conversion within 30 min [31]. Under the same conditions, catalysts **Ru-pqcame** and **6** were less effective over a period of 180 min (entries 54, 55). However, catalyst **7-Cl** bearing the non-substituted pq ligand has shown better activity than that of cis-[RuCl_2_(PPh_3_)_2_(pq)], exhibiting a quantitative yield, within the limits of instrumental detection, after 150 min. All other complexes hold the same trend followed for the reduction in acetophenone. As an exception, complex **11** reached a 99% conversion which is higher when compared to that of acetophenone (90%).

To this end, we performed additional experiments in order to propose key intermediates (active catalysts) that are involved in the TH process reported herein. Thus, we examined the reaction of complex **4** as the catalyst precursor (Cat) with KO*i*Pr (base) in 2-propanol, followed by subsequent heating to reflux for ~120 min. The catalyst (Cat) to base molar ratios ranged from 1:3 to 1:10. A characteristic pine green solid was isolated, after evaporation of the solvent, when the Cat to base molar ratio was 1:10. The ^1^H NMR spectrum of the crude product (in CH_3_OD), in the hydridic region, revealed the presence of a broad singlet resonance at δ—0.91 ppm (approximately 12% formation, based on integration, Figure S82), which can be attributed to the formation of Ru-H species [57]. Based on these findings, Ru-hydrides can be considered as the active species, and an inner-sphere mechanism may be proposed. Apparently, upon the addition of the base, a ruthenium(II) alkoxide is potentially formed, which then undergoes β-H elimination, leading to the formation of the ruthenium-H species that can be considered as the catalytically active species [58,59].

## 3. Materials and Methods

The synthesis of the ruthenium(II) complexes was carried out under an argon atmosphere using standard Schlenk techniques, while aerobic conditions were used for the synthesis of the organic ligands. Analytical grade solvents were used and when required were distilled and dried before use, according to standard methods. The distilled solvents were stored over molecular sieves under an argon atmosphere. RuCl_3_∙H_2_O was purchased from Riedel de Haën and α-terpinene was purchased from Sigma-Aldrich (Kyoto, Japan). Ligand precursors 2,2′-pyridyl-quinoline-4-carboxylic acid (**pqca**), 8-methyl-2,2′-pyridyl-quinoline-4-carboxylic acid (**8-Mepqca**), 6′-methyl-2,2′-pyridyl-quinoline-4-carboxylic acid (**6′-Mepqca**), 8,6′-dimethyl-2,2′-pyridyl-quinoline-4-carboxylic acid (**8,6′-Me_2_pqca**), 2,2′-pyridyl-quinoline (**pq**) [36,37,38] and 2,2′-biquinoline (**biq**) were prepared according to the literature reports [40,41,42]. Ligands 4-methyl-2,2′-pyridyl-quinoline (**4-Mepq**), methyl 2,2′-pyridyl-quinoline-4-carboxylate (**pqcame**) and dimethyl 2,2′-biquinoline-4,4′-dicarboxylate (**biqcame**) [43,44], as well as the ruthenium(II) starting material [Ru(η^6^-*p*-cymene)(μ-Cl)Cl]_2_ [60], were made according to the literature procedures. The [Ru(η^6^-*p*-cymene)(pqcame)Cl][PF_6_] (**Ru-pqcame**) complex was synthesized according to the literature [57]. Infrared spectra (FT-IR) were recorded on IR Affinity-1 SHIMADZU as potassium bromide pellets in the spectral range 4000–400 cm^−1^. Elemental analyses were obtained from the Microanalysis Center of Institut für Anorganische Chemie Universität Bonn. ^1^H (16 scans) and ^13^C NMR (1024 scans) spectra were recorded on Varian 200 MHz and on Bruker Avance Neo 400 MHz. The probe used was a Z163739_0063 (PI HR-400-S1-BBF/H/D-5.0-Z SP) type. NMR spectra of metal complexes were assigned using the ^1^H–^1^H COSY (32 scans), ^1^H–^13^C HSQC (64 scans) and ^1^H–^13^C HMBC (128 scans) methods. *J* values are given in Hz. Absorption spectra were recorded with a CARY 3E UV-vis spectrometer. For the X-ray diffractions, Bruker D8-Venture, Bruker APEX-II CCD and Bruker X8-Kappa Apex II were used.

### 3.1. Synthesis and Characterization

#### 3.1.1. Synthesis of the Organic Ligands **8-Mepq, 6′-Mepq, 8,6′-Me_2_pq** and **pq**

In a Pyrex glass tube, 1 mmol of the carboxylic acid precursor (**pqca**, **8-Mepqca**, **6′-Mepqca** or **8,6′-Me_2_pqca)** was heated rapidly to reach the melting point temperature. The resulting black solid was left to cool at room temperature, treated with hot petroleum ether (25 mL) and, subsequently, active carbon was added. After filtration, the clear pale-yellow filtrates were evaporated to dryness affording the required ligands.

**8-methyl-2,2′-pyridyl-quinoline (8-Mepq).** Off-white solid. Yield: (0.140 g, 67%). IR (KBr, *ν*_max_/cm^−1^: 3038 (w, *ν*(C–H)_arom_), 2919 (w, *ν*_as_(C–H)_aliph_), 1597 (m, *ν*(C = C)), 1446 (m, *ν*(C = N)). ^1^H NMR (CDCl_3_, 200 MHz, 298 K) *δ*_H_/ppm 8.74 (2H, m, H5/H6′), 8.58 (1H, d, *J* = 6.0, H4), 8.25 (1H, d, *J* = 8.0, H3), 7.86 (1H, t, *J* = 8.0, H6), 7.69 (1H, d, *J* = 8.0, H3′), 7.58 (1H, d, *J* = 6.0, H5′), 7.45 (1H, d, *J* = 6.0, H4’), 7.35 (1H, m, H7), 2.92 (3H, s, C*H*_3_-quin). ^13^C{^1^H} NMR (CDCl_3_, 50 MHz, 298 K) *δ*_C_/ppm 156.89 (C8), 154.82 (C2), 149.16 (C8a), 146.96 (C3), 137.83 (C6), 137.13 (C6′), 136.98 (C4′), 129.72 (C2′), 128.36 (C3′), 126.67 (C4a), 125.70 (C4), 124.02 (C5), 121.93 (C5′), 118.53 (C7), 18.00 (*C*H_3_-quin). ESI-HRMS (MeOH, positive mode): *m*/*z* 221.1090 [C_15_H_12_N_2_ + H]^+^ (calc. 221.1073), *m*/*z* 243.0888 [C_15_H_12_N_2_ + Na]^+^ (calc. 243.0893), *m*/*z* 441.2020 [C_30_H_24_N_4_ + H]^+^ (calc. 441.2074), *m*/*z* 463.1966 [C_30_H_24_N_4_ + Na]^+^ (calc. 463.1893).

**6′-methyl-2,2′-pyridyl-quinoline (6′-Mepq**). Off-white solid. Yield: (0.135 g, 65%). IR (KBr, *ν*_max_/cm^−1^): 3057 (w, *ν*(C–H)_arom_), 2921 (w, *ν*_as_(C–H)_aliph_), 1616 (w, *ν*(C = C)), 1592 (s, *ν*(C = C)), 1453 (s, *ν*(C = N)). ^1^H NMR (CDCl_3_, 200 MHz) *δ*_H_/ppm 8.60 (1H, d, *J* = 8.0, H5), 8.45 (1H, d, *J* = 8.0, H4), 8.26 (1H, d, *J* = 8.0, H3), 8.18 (1H, d, *J* = 8.0, H8), 7.84 (1H, d, *J* = 6.0, H5′), 7.75 (2H, m, H6/H4′), 7.54 (1H, t, *J* = 8.0, H7), 7.21 (1H, d, *J* = 6.0, H3′), 2.68 (3H, s, C*H*_3_-py). ^13^C{^1^H} (CDCl_3_, 50 MHz) *δ*_C_/ppm 158.07 (C2), 156.69 (C2′), 155.89 (C6′), 148.09 (C4′), 137.26 (C5′), 136.82 (C8a), 129.94 (C6), 129.59 (C7), 128.36 (C5), 127.74 (C8), 126.74 (C3), 123.72 (C4), 119.32 (C3′), 119.01 (C4a), 24.81 (*C*H_3_-py). ESI-HRMS (MeOH, positive mode): *m*/*z* 221.1095 [C_15_H_12_N_2_ + H]^+^ (calc. 221.1073), 243.0895 [C_15_H_12_N_2_ + Na]^+^ (calc. 243.0892), 259.0630 [C_15_H_12_N_2_ + K]^+^ (calc. 259.0632), 463.1892 [C_30_H_24_N_4_ + Na]^+^ (calc. 463.1893).

**8,6′-dimethyl-2,2′-pyridyl-quinoline (8,6′-Me_2_pq)**. Off-white solid. Yield: (0.152 g, 72%). IR (KBr, *ν*_max_/cm^−1^): 3054 (w, *ν*(C–H)_arom_), 2918 (m, *ν*_as_(C–H)_aliph_), 1614 (w, *ν*(C = C)), 1598 (vs, *ν*(C = C)), 1453 (s, *ν*(C = N)). ^1^H NMR (CDCl_3_, 400 MHz) *δ*_H_**/**ppm 8.64 (1H, d, *J* = 8.0, H3), 8.56 (1H, d, *J* = 8.0, H3′), 8.23 (1H, d, *J* = 8.0, H4), 7.74 (1H, t, *J* = 6.0, H4′), 7.68 (1H, d, *J* = 8.0, H5), 7.57 (1H, d, *J* = 8.0, H7), 7.43 (1H, t, *J* = 8.0, H6), 7.19 (1H, d, *J* = 8.0, H5′), 2.93 (3H, s, C*H*_3_-py), 2.69 (3H, s, C*H*_3_-quin). ^13^C{^1^H} (CDCl_3_, 50 MHz) *δ*_C_**/**ppm 157.79 (C8), 156.23 (C6′), 155.09 (C2), 146.94 (C8a), 137.07 (C4′), 136.91 (C4), 129.57 (C8), 128.26 (C7), 126.45 (C6), 125.64 (C5), 123.48 (C5′), 118.88 (C3′), 118.65 (C3), 27.74 (*C*H_3_-py), 17.97 (*C*H_3_-quin**)**. ESI-HRMS (MeOH, positive mode): 235.1277 [C_16_H_14_N_2_ + H]^+^ (calc. 235.1229), 257.1056 [C_16_H_14_N_2_ + Na]^+^ (calc. 257.1049), 273.0791 [C_16_H_14_N_2_ + K]^+^ (calc. 273.0788), 491.2214 [C_32_H_28_N_4_ + Na]^+^ (calc. 491.2206).

**2,2′-pyridyl-quinoline (pq).** Light yellow solid. Yield: (0.15 g, 60%). IR (KBr, *ν*_max_/cm^−1^): 3056 (w, *ν*(C–H)_arom_), 2925 (w, *ν*_as_(C–H)_aliph_), 1596 (vs, *ν*(C = C)), 1450 (m, *ν*(C = N)), 778(s) and 713 (m) [δ(C-H) out of plane]. ^1^H NMR (CDCl_3_, 400 MHz) *δ*_H_**/**ppm 8.75 (1H, td, *J* = 4.0 and 8.0, H6′), 8.67 (1H, dt, *J* = 8.0, H8), 8.57 (1H, d, *J* = 8.0, H3), 8.30 (1H, d, *J* = 8.0, H4), 8.19 (1H, d, *J* = 8.0, H5), 7.90 (2H, dd, *J* = 4.0 and 8.0, H3′/H4′), 7.75 (1H, ddd, *J* = 4.0 and 8.0, H7), 7.57 (1H, ddd, *J* = 4.0 and 8.0, H6), 7.38 (1H, ddd, td, *J* = 4.0 and 8.0, H5′).

#### 3.1.2. Synthesis of the Organic Ligand **4,6′-Me_2_pq**

In a 50 mL round-bottomed flask and under an argon atmosphere, 2-aminoacetophenone (0.203 g, 1.5 mmol) and 6-methyl-2-acetylpyridine (0.182 g, 1.5 mmol) were dissolved in dry ethanol (5 mL) and, subsequently, 3 drops of a 66% (w/w) aqueous KOH solution were added. The resulting mixture was refluxed for approximately 18 h and then cooled at room temperature. The pH of the obtained solution was neutralized, using 1 M HCl_(aq)_.and the organic solvent was rotary evaporated. The obtained aqueous phase was extracted with CH_2_Cl_2_ (3 × 10 mL) and the organic layer was treated with anhydrous MgSO_4_. After filtration, the filtrate was evaporated to dryness to yield a yellow oily residue. The residue was treated with a methanol/water mixture (1:9, v/v) to afford an off-white solid, that was dried in a vacuum desiccator under P_2_O_5_. Yield: (0.210 g, 60%). IR (KBr, *ν*_max_/cm^−1^): 3054 (w, *ν*(C–H)_arom_), 2922 (m, *ν*_as_(C–H)_aliph_), 1614 (w, *ν*(C = C)), 1597 (s, *ν*(C = C)), 1453 (s, *ν*(C = N)). ^1^H NMR (CDCl_3_, 400 MHz) *δ*_H_**/**ppm 8.43 (2H, m, H3/H5′), 8.18 (1H, d, *J* = 8.0, H8), 8.01 (1H, d, *J* = 8.0, H5), 7.73 (2H, m, H4′/H6), 7.56 (1H, t, *J* = 12.0, H7), 7.20 (1H, d, *J* = 8.0, H3′), 2.80 (3H, s, C*H*_3_-quin), 2.70 (3H, s, C*H*_3_-py). ^13^C{^1^H} NMR (CDCl_3_, 100 MHz) *δ*_C_**/**ppm 157.96 (C4), 156.22 (C2), 156.12 (C2′), 147.99 (C8α), 144.98 (C6′), 137.19 (C6), 130.52 (C8), 129.24 (C4′), 128.36 (C4α), 126.46 (C7), 123.88 (C3′), 123.80 (C5), 119.74 (C5′), 118.98 (C3), 24.83 (6′-CH_3_), 19.05 (4-CH_3_).

#### 3.1.3. General Synthetic Procedure of Complexes **1**–**8**

In a Schleck tube and under an argon atmosphere, 0.095 g (0.15 mmol) of [Ru(η^6^-*p*-cymene)(μ-Cl)Cl]_2_ and 2.3 mol equivalents (0.36 mmol) of the corresponding organic ligand (L) were added. Upon addition of dry methanol (10 mL), the solution obtained an orange–yellow color, and the mixture was stirred for 18 h at room temperature. The solvent was rotary evaporated, and the oily residue obtained was treated with diethyl ether (2 × 3 mL). The resulting solid was dissolved in water (5 mL) and, subsequently, a few drops of a saturated aqueous solution of KPF_6_ were added. Upon addition, a yellow precipitate formed, and the mixture was stirred for 30 min more, to ensure completion of the reaction. After filtration, the yellow solid was washed first with water (5 mL) then with chloroform (3 mL) and finally with diethyl ether (2 × 5 mL) and was dried in vacuo at 50 °C.

#### 3.1.4. Data for Complexes **1**–**8**

[Ru(η^6^-*p*-cymene)(**8-Mepq**)Cl][PF_6_] (**1**). Yield: (0.088 g, 92%). Found: C, 46.04; H, 4.15; N, 4.25. C_25_H_26_ClF_6_N_2_PRu∙H_2_O requires C, 45.91; H, 4.32; N, 4.28%. IR (KBr, *ν*_max_/cm^−1^): 3051 (w, *ν*(C–H)_arom_, 3032 (w, *ν*(C–H)_arom_), 2873 (w, *ν*_as_(C–H)_aliph_), 1572 (m, *ν*(C = N)), 1477 (s, *ν*(C = C)), 1383 (m, *ν*(C = C)), 841 (vs, *ν*(P–F)), 557 (s, *ν*(P–F)). UV-vis (Me_2_CO, 1.0 × 10^−4^): λ_max_/nm 347 (ε/dm^3^ mol^−1^ cm^−1^ 7600), 453 (1300). ^1^H NMR (400 MHz, Me_2_CO-d_6_) *δ*_H_**/**ppm 9.39 (1H, d, *J* = 8.0, H6′), 8.89 (1H, d, *J* = 8.0, H3), 8.64 (1H, d, *J* = 8.0, H5), 8.59 (1H, d, *J* = 8.0, H4), 8.38 (1H, m, *J* = 8.0, H4′), 8.14 (1H, d, *J* = 8.0, H7), 8.02 (1H, d, *J* = 8.0, H3′), 7.89 (2H, t, *J* = 4.0, H5′/H6), 6.01 (1H, d, *J* = 4.0, H-cym_ar_), 5.83 (1H, d, *J* = 4.0, H-cym_ar_), 5.57 (1H, d, *J* = 4.0, H-cym_ar_), 5.49 (1H, d, *J* = 4.0, H-cym_ar_), 3.31 (3H, s, CH_3_-quin), 2.11 (1H, m, C*H*-(CH_3_)_2_), 1.84 (3H, s, C*H*_3_-cym), 1.02 (3H, d, *J* = 8.0, CH-(C*H*_3_)_2_), 0.90 (3H, d, *J* = 8.0, *CH*-(C*H*_3_)_2_). ^13^C{^1^H} NMR (100 MHz, Me_2_CO-d_6_) *δ*_C_**/**ppm 159.24 (C6′), 156.26 (C2), 155.87 (C8), 152.21 (C8a), 141.91 (C3), 140.23 (C4′), 136.18 (C3′), 135.80 (C4a), 130.78 (C6), 129.12 (C5′), 127.51 (C5), 127.23 (C7), 125.13 (C2′), 119.60 (C4), 106.57 (CH_3_-*C*_cym_), 100.51 (*C*_cym_-CH-(CH_3_)_2_), 86.89 (C-C_cym-ar_), 85.64 (C-C_cym-ar_), 84.20 (C-C_cym-ar_), 83.29 (C-C_cym-ar_), 30.63 (*C*H-(CH_3_)_2_), 21.83 (*C*H_3_-quin), 21.27 (CH-(*C*H_3_)_2_), 20.76 (CH-(*C*H_3_)_2_), 16.81 (*C*H_3_-cym).

[Ru(η^6^-*p*-cymene)(**6′-Mepq**)Cl][PF_6_] (**2**). Yield: (0.085 g, 89%). Found: C, 46.81; H, 4.23; N, 4.36. C_25_H_26_ClF_6_N_2_PRu requires C, 47.21; H, 4.12; N, 4.40%. IR (KBr, *ν*_max_/cm^−1^): 3099 (w, *ν*(C–H)_arom_), 2876 (w, *ν*_as_(C–H)_aliph_), 1581 (w, *ν*(C = N)), 1479 (m, *ν*(C = C)), 1379 (w, *ν*(C = C)), 840 (vs, *ν*(P–F)), 557 (s, *ν*(P–F)). UV-vis (Me_2_CO, 1.0 × 10^−4^ mol dm^−3^): λ_max_/nm 341 (ε/dm^3^ mol^−1^ cm^−1^ 2040), 356 (24,305), 427 (3513). ^1^H NMR (400 MHz, Me_2_CO-d_6_) *δ*_H_**/**ppm 9.06 (d, 1H, *J* = 8.0, H8), 8.83 (d, 1H, *J* = 8.0, H3), 8.63 (d, 2H, *J* = 8.0, H4/H5), 8.23 (m, 2H, H3′/H4′), 8.08 (t, 1H, *J* = 8.0, H7), 7.89 (m, 2H, H6/H5′), 6.18 (1H, d, *J* = 4.0, H-cym_ar_), 6.05 (1H, d, *J* = 8.0, H-cym_ar_), 5.90 (1H, d, *J* = 8.0, H-cym_ar_), 5.80 (1H, d, *J* = 8.0, H-cym_ar_), 3.34 (3H, s, CH_3_-py), 2.32 (4H, m, C*H*_3_-cym/C*H*-(CH_3_)_2_), 1.00 (3H, d, *J* = 8.0, CH-(C*H*_3_)_2_), 0.93 (3H, d, *J* = 8.0, CH-(C*H*_3_)_2_). ^13^C{^1^H} NMR (100 MHz, Me_2_CO-d_6_) *δ*_C_**/**ppm 166.76 (C6′), 158.05 (C2′), 156.69 (C2), 150.73 (C8a), 141.97 (C3), 140.67 (C4′), 133.44 (C7), 130.62 (C8), 130.42 (C4a), 130.32 (C3′), 129.86 (C6), 129.72 (C5′), 123.61 (C5), 120.03 (C4), 106.74 (CH_3_-*C*_cym_), 104.84 (*C*_cym_-CH-(CH_3_)_2_), 86.82 (C-C_cym-ar_), 86.47 (C-C_cym-ar_), 86.26 (C-C_cym-ar_), 86.12 (C-C_cym-ar_), 31.44 (*C*H-(CH_3_)_2_), 28.31 (*C*H_3_-py), 22.18 (CH-(*C*H_3_)_2_), 22.10 (CH-(*C*H_3_)_2_), 18.32 (*C*H_3_-cym).

[Ru(η^6^-*p*-cymene)(**8,6′-Me_2_pq**)Cl][PF_6_] (**3**). Yield: (0.088 g, 90%). Found: C, 47.99; H, 4.57; N, 4.28. C_26_H_28_ClF_6_N_2_PRu requires C, 48.04; H, 4.34; N, 4.31%. IR (KBr, *ν*_max_/cm^−1^): 3079 (w, *ν*(C–H)_arom_), 2928 (m, *ν*_as_(C–H)_aliph_), 1571 (w, *ν*(C = N)), 1472 (m, *ν*(C = C)), 1383 (w, *ν*(C = C)), 838 (vs, *ν(*P–F)), 557 (s, *ν*(P–F)). UV-vis (Me_2_CO, 1.0 × 10^−4^ mol dm^−3^): λ_max_/nm 339 (ε/dm^3^ mol^−1^ cm^−1^ 12,250), 354 (14,100), 424 (2100). ^1^H NMR (400 MHz, Me_2_CO-*d*_6_) *δ*_H_**/**ppm 8.88 (1H, d, *J* = 8.0, H3), 8.55 (1H, d, *J* = 8.0, H4), 8.45 (1H, d, *J* = 8.0, H3′), 8.23 (1H, t, *J* = 8.0, H4′), 8.12 (1H, d, *J* = 8.0, H5), 7.99 (1H, d, *J* = 8.0, H7), 7.86 (2H, m, H6/H5′), 6.13 (1H, d, *J* = 4.0, H-cym_ar_), 5.71 (1H, d, *J* = 8.0, H-cym_ar_), 5.45 (1H, d, *J* = 8.0, H-cym_ar_), 4.95 (1H, d, *J* = 8.0, H-cym_ar_), 3.30 (3H, s, CH_3_-py), 3.27 (3H, s, CH_3_-quin), 2.24 (3H, s, CH_3_-cym), 2.02 (1H, m, C*H*-(CH_3_)_2_), 0.90 (3 H, d, *J* = 8.0, CH-(C*H*_3_)_2_), 0.85 (3H, d, *J* = 8.0, CH-(C*H*_3_)_2_). ^13^C{^1^H} NMR (100 MHz, Me_2_CO-*d*_6_) *δ*_C_**/**ppm 165.39 (C6′), 160.28 (C8), 156.77 (C2), 152.70 (C2′), 142.43 (C8a), 140.12 (C3), 136.09 (C4′), 135.88 (C4a), 130.37 (C3′), 129.09 (C6), 128.78 (C5′), 127.41 (C5), 123.69 (C7), 119.64 (C4), 106.0 (CH_3_-*C*_cym_), 105.0 (*C*_cym_-CH-(CH_3_)_2_*)*, 85.63 (C-C_cym-ar_), 85.16 (C-C_cym-ar_), 82.88 (C-C_cym-ar_), 82.11 (C-C_cym-ar_), 30.42 (*C*H-(CH_3_)_2_), 29.39 (*C*H_3_-py), 22.50 (*C*H_3_-quin), 22.04 (CH-(*C*H_3_)_2_), 20.42 (CH-(*C*H_3_)_2_), 17.27 (*C*H_3_-cym).

[Ru(η^6^-*p*-cymene)(**4,6**′**-Me_2_pq**)Cl][PF_6_] (**4**). Yield: (0.088 g, 90%). Found: C, 47.41; H, 4.50; N, 4.04. C_26_H_28_ClF_6_N_2_PRu∙0.5H_2_O requires C, 47.39; H, 4.28; N, 4.25%. IR (KBr, *ν*_max_/cm^−1^): 3087 (w, *ν*(C–H)_arom_), 2924 (m, *ν*_as_(C–H)_aliph_), 1597 (s, *ν*(C = N)), 1472 (s, *ν*(C = C)), 1386 (w, *ν*(C = C)), 836 (vs, *ν*(P–F)), 557 (s, *ν*(P–F)). UV-vis (Me_2_CO, 1.0 × 10^−4^): λ_max_/nm 338 (ε/dm^3^ mol^−1^ cm^−1^ 13,000), 347 (14,500), 423 (2200). ^1^H NMR (400 MHz, Me_2_CO-*d*_6_) *δ*_H_**/**ppm 9.05 (1H, d, *J* = 4.0, H8), 8.61 (1H, d, *J* = 8.0, H5), 8.55 (1H, s, H3), 8.31 (1H, d, *J* = 8.0, H3′), 8.21 (1H, t, *J* = 8.0, H4′), 8.05 (1H, t, *J* = 8.0, H7), 7.91 (1H, t, *J* = 8.0, H6), 7.85 (1H, d, *J* = 8.0, H5′), 6.15 (1H, d, *J* = 8.0, H-cym_ar_), 6.01 (1H, d, *J* = 4.0, H-cym_ar_), 5.86 (1H, d, *J* = 8.0, H-cym_ar_), 5.75 (1H, d, *J* = 8.0, H-cym_ar_), 3.33 (3H, s, C*H*_3_-py), 3.00 (3H, s, C*H*_3_-quin), 2.29 (4H, m, C*H*_3_-cym/C*H*-(CH_3_)_2_), 0.99 (3H, d, *J* = 8.0, CH-(C*H*_3_)_2_), 0.93 (3H, d, *J* = 8.0, CH-(C*H*_3_)_2_). ^13^C{^1^H} NMR (100 MHz, Me_2_CO-*d*_6_) *δ*_C_**/**ppm 166.68(C6′), 157.38 (C2′), 156.38 (C2), 151.76 (C4), 150.17 (C9), 140.59 (C4′), 132.92 (C8), 131.15 (C6), 130.18 (C9), 129.62 (C5′), 126.11 (C3′), 123.37 (C6), 120.83 (C3), 106.68 (CH_3_-*C*_cym_), 104.56 (*C*_cym_-CH-(CH_3_)_2_), 86.38 (C-C_cym-ar_), 86.22 (C-C_cym-ar_), 85.92 (C-C_cym-ar_), 31.44 (*C*H-(CH_3_)_2_), 28.32 (*C*H_3_-py), 22.22 (*C*H_3_-quin), 22.06 (CH-(*C*H_3_)_2_), 19.26 (CH-(*C*H_3_)_2_), 18.31 (*C*H_3_-cym), 13.26 (4-CH_3_).

[Ru(η^6^-*p*-cymene)(**4-Mepq**)Cl][PF_6_] (**5**). Yield: (0.080 g, 84%). Found: C, 44.01; H, 4.86; N, 3.99. C_25_H_26_ClF_6_N_2_PRu∙3H_2_O requires C, 43.51; H, 4.67; N, 4.06%. IR (KBr, *ν*_max_/cm^−1^): 3082 (w, *ν*(C–H)_arom_), 2877 (w, *ν*_as_(C–H)_aliph_), 1596 (s, *ν*(C = N)), 1484 (m, *ν*(C = C)), 1384 (m, *ν*(C = C)), 839 (vs. br, *ν*(P–F)), 557 (s, *ν*(P–F)). UV-vis (Me_2_CO, 1.0 × 10^−4^): λ_max_/nm 335 (ε/dm^3^ mol^−1^ cm^−1^ 12,600), 348 (13,000), 412 (3000). ^1^H NMR (400 MHz, Me_2_CO-d_6_) *δ*_H_**/**ppm 9.59 (1H, d, *J* = 8.0, H8), 8.98 (1H, d, *J* = 8.0, H6′), 8.74 (1H, d, *J* = 8.0, H5), 8.58 (1H, s, H3), 8.32 (2H, m, *J* = 8.0, H3′/H6), 8.09 (1H, td, *J* = 8.0, H4′), 7.95(1H, t, *J* = 8.0, H5′), 7.86 (1H, t, *J* = 8.0, H7), 6.15 (1H, d, *J* = 8.0, H-cym_ar_), 6.06 (1H, d, *J* = 8.0, H-cym_ar_), 6.00 (2H, m, H-cym_ar_), 3.00 (3H, s, CH_3_-quin), 2.41 (1H, sept, *J* = 5.0, C*H*-(CH_3_)_2_), 2.29 (3H, s, CH_3_-cym), 0.95 (3H, d, *J* = 5.0, CH-(C*H*_3_)_2_), 0.91 (3H, d, *J* = 5.0, CH-(C*H*_3_)_2_).^13^C{^1^H} NMR (100 MHz, Me_2_CO-d_6_) *δ*_C_**/**ppm 157.12 (2C, C8/C2), 156.51 (C4a), 156.39 (C2′), 151.71 (C4), 149.75 (C8a), 140.95 (C6), 133.05 (C4′), 131.58 (C6′), 130.32 (C5′), 128.94 (C7), 126.18 (C3′), 125.80 (C5), 120.83 (C3) 106.46 (CH_3_-*C*_cym_), 105.18 (*C*_cym_-CH-(CH_3_)_2_), 87.78 (C-C_cym-ar_), 86.80 (C-C_cym-ar_), 86.57 (C-C_cym-ar_), 85.76 (C-C_cym-ar_), 31.75 (*C*H-(CH_3_)_2_), 22.29 (CH-(*C*H_3_)_2_), 21.85 (CH-(*C*H_3_)_2_), 19.22 (CH_3_-C_cym_), 18.71 (CH_3-_quin).

**[**Ru(η^6^-*p*-cymene)(**biqcame**)Cl][Cl] (**6-Cl**). According to the general synthetic procedure 2.4 but under reflux conditions. Yield: (0.108 g, 95%). IR (KBr, *ν*_max_/cm^−1^): 3040 (w, *ν*(C–H)_arom_), 2962 (w, *ν*_as_(C–H)_aliph_), 1597 (vs, *ν*(C = N)), 1484 (m, *ν*(C = C)), 1383 (w, *ν*(C = C)), ^1^H NMR (400 MHz, 298K, CDCl_3_) *δ*_H_**/**ppm 9.14 (2H, d, *J* = 8.0, H8/H8′), 9.06 (2H, s, H3/H3′), 8.95 (2H, d, *J* = 8.0, H5/H5′), 8.07 (2H, t, *J* = 8.0, H7/H7′), 7.92 (2H, t, *J* = 8.0, H6/H6′), 5.98 (2H, d, *J* = 8.0, H-cym_ar_), 5.81 (2H, d, *J* = 8.0, H-cym_ar_), 4.19 (6H, s, COOCH_3_), 2.57 (1H, sept, *J* = 4.0, C*H*-(CH_3_)_2_), 2.10 (3H, s, C*H*_3_-cym), 1.11 (6H, d, *J* = 4.0, CH-(C*H*_3_)_2_). ^13^C{^1^H} NMR (100 MHz, CDCl_3_) *δ*_C_**/**ppm 158.58 (CO/CO’), 132.91 (C7/C7′), 131.62 (C6/C6′), 130.32 (C8/C8′), 127.09 (C5/C5′), 124.26 (C3/C3′), 87.62 (2C, C-C_cym_), 86.32 (2C, C-C_cym_), 53.32 (O-CH_3_/O-CH_3_′), 30.68 (*C*H-(CH_3_)_2_), 29.28 (CH-(*C*H_3_)_2_), 22.27 (CH-(*C*H_3_)_2_), 17.74 (CH_3_-cym).

[Ru(η^6^-*p*-cymene)(**biqcame**)Cl][PF_6_] (**6**). As for the synthesis of **6-Cl** and then according to the general synthetic procedure 2.4. Yield: (0.115 g, 95%). Found: C, 48.46; H, 4.15; N, 3.36. C_32_H_30_ClF_6_N_2_O_4_PRu requires C, 48.77; H, 3.84; N, 3.55%. IR (KBr, *ν*_max_/cm^−1^): 3093 (w, *ν*(C–H)_arom_), 3059 (w, *ν*(C–H)_arom_), 2926 (m, *ν*_as_(C–H)_aliph_), 1726 (s, *ν*(C = O)), 1583 (m, *ν*(C = N)), 1474 (w, *ν*(C = C)), 1381 (w, *ν*(C = C)), 835 (vs, *ν*(P-F)), 557 (s, *ν*(P–F)). UV-vis (Me_2_CO, 1.0 × 10^−4^): λ_max_/nm 371 (ε/dm^3^ mol^−1^ cm^−1^ 21,100), 387 (26,300), 476 (3800). ^1^H NMR (400 MHz, 298K, Me_2_CO-d_6_) *δ*_H_**/**ppm 9.29 (4H, m, H3/H8/H3′/H8′), 8.85 (2H, d, *J* = 8.0, H5/H5′), 8.20 (2H, t, *J* = 8.0, H7/H7′), 8.07 (2H, t, *J* = 8.0, H6/H6′), 6.07 (2H, d, *J* = 8.0, H-cym_ar_), 5.99 (2H, d, *J* = 8.0, H-cym_ar_), 4.17 (6H, s, COOC*H*_3_), 2.40 (3H, s, C*H*_3_-cym), 2.34 (1H, m, C*H*-(CH_3_)_2_), 0.96 (6H, d, *J* = 4.0, CH-(C*H*_3_)_2_). ^13^C{^1^H} NMR (100 MHz, Me_2_CO-d_6_) *δ*_C_**/**ppm 164.88 (C°/C°′), 156.16 (C8a/C8a′), 151.07 (C2/C2′), 139.96 (C4/C4′), 132.95 (C7/C7′), 131.48 (C6/C6′), 130.37 (C8/C8′), 126.53 (C5/C5′), 126.20 (C4a/C4a′), 120.93 (C3/C3′), 107.89 (CH_3_-*C*_cym_), 103.79 (*C*_cym_-CH-(CH_3_)_2_), 87.70(C-C_cym-ar_), 86.44 (C-C_cym-ar_), 78.42 (C-C_cym-ar_), 78.10 (C-C_cym-ar_), 53.13 (OCH_3_/OCH_3_′), 30.68 (*C*H-(CH_3_)_2_), 24.90 (CH-(*C*H_3_)_2_), 21.31 (CH-(*C*H_3_)_2_), 17.34 (CH-(*C*H_3_)_2_.

[Ru(η^6^-*p*-cymene)(**pq**)Cl][Cl] (**7-Cl**). Similar procedure to that of the published synthetic procedure, but without treatment with KPF_6_. Yield: 70 mg (75%). ^1^H NMR (400 MHz, Me_2_CO-d_6_) *δ*_H_**/**ppm 9.59 (1H, d, *J* = 8.0, H6′), 8.96 (1H, d, *J* = 8.0, H8), 8.82 (1H, d, *J* = 8.0, H3), 8.74 (1H, d, *J* = 8.0, H4), 8.63 (1H, d, *J* = 8.0, H3′), 8.35 (1H, t, *J* = 8.0, H4′), 8.23 (1H, d, *J* = 8.0, H5), 8.11 (1H, t, *J* = 8.0, H7), 7.92 (1H, t, *J* = 8.0, H6), 7.87 (1H, t, *J* = 4.0, H5′), 6.16 (1H, d, *J* = 8.0, H-cym_ar_), 6.08 (1H, d, *J* = 8.0, H-cym_ar_), 6.01 (2H, m, H-cym_ar_), 2.42 (1H, m, C*H*-(*C*H_3_)_2_), 2.28 (3H, s, C*H*_3_-cym), 0.94 (3H, d, *J* = 4.0, CH-(C*H*_3_)_2_), 0.90 (3H, d, *J* = 4.0, CH-(C*H*_3_)_2_). ^13^C{^1^H} NMR (100 MHz, 298K, Me_2_CO-d_6_) *δ*_C_**/**ppm 157.18 (C2′), 157.15 (C6′), 156.16 (C2), 150.30 (C8a), 141.86 (C3), 141.01 (C4′), 133.57 (C7), 131.00 (C8), 130.53 (C4), 130.49 (C6), 129.92 (C5), 129.06 (C5′), 126.02 (C4a), 119.99 (C3′), 106.59 (CH_3_-*C*_cym_), 105.32 (*C*_cym_-CH-(CH_3_)_2_), 87.90 (C-C_cym-ar_), 86.88 (C-C_cym-ar_), 86.48 (C-C_cym-ar_), 85.63 (C-C_cym-ar_), 31.72 (C*H*-(CH_3_)_2_), 22.30 (CH-(C*H*_3_)_2_), 21.71 (CH-(C*H*_3_)_2_), 18.69 (CH_3_-cym).

[Ru(η^6^-*p*-cymene)(**biq**)Cl][PF_6_] (**8**). Yield: (0.09 g, 85%). Found: C, 49.52; H, 4.21; N, 3.94. C_28_H_26_ClF_6_N_2_O_4_PRu requires C, 50.04; H, 4.17; N, 3.55%. IR (KBr, *ν*_max_/cm^−1^): 3069 (w, *ν*(C–H)_arom_), 2924 (w, *ν*_as_(C–H)_aliph_), 1596 (m, ν(C = N)), 1472 (m, *ν*(C = C)), 1385 (m, *ν*(C = C)), 841 (s, *ν*(P–F), 557 (s, *ν*(P–F)). UV-vis (Me_2_CO, 1.0 × 10^−4^ mol dm^−3^): λ_max_/nm 355 (ε/dm^3^ mol^−1^ cm^−1^ 21,000), 373 (31,100), 450 (2900). ^1^H NMR (400 MHz, Me_2_CO-d_6_) *δ*_H_**/**ppm 9.14 (2H, d, *J* = 8.0, H8/H8′), 8.85 (2H, d, *J* = 8.0 Hz, H4/H4′), 8.76 (2H, d, *J* = 8.0, H3/H3′), 8.21 (2H, d, *J* = 8.0, H5/H5′), 8.12 (2H, t, *J* = 8.0, H7/H7′), 7.93 (2H, t, *J* = 8.0, H6/H6′), 5.98 (2H, d, *J* = 8.0, H-cym_ar_), 5.87 (2H, d, *J* = 8.0, H-cym_ar_), 2.40 (3H, s, C*H*_3_-cym), 2.16 (1H, m, C*H*-(CH_3_)_2_), 0.89 (6H, d, *J* = 8.0, -CH-(C*H*_3_)_2_). ^13^C{^1^H} NMR (100 MHz, Me_2_CO-d_6_) *δ*_C_**/**ppm 156.72 (C8a/C8a′), 150.06 (C2/C2′), 141.27 (C4/C4′), 136.81 (C7/C7′), 132.94 (C6/C6′), 129.71 (C8/C8′), 129.63 (C5/C5′), 129.12 (C4a/C4a′), 120.16 (C3/C3′), 106.19 (CH_3_-*C*_cym_), 103.67 (*C*_cym_-CH-(CH_3_)_2_), 86.73 (2C-C_cym-ar_), 86.21 (2C-C_cym-ar_), 30.55 (*C*H-(CH_3_)_2_), 21.18 (CH-(*C*H_3_)_2_), 17.41 (*C*H_3_-cym).

### 3.2. General Synthetic Procedure of Complexes ***9***–***11***

In a Schlenk flask and under an argon atmosphere, 0.095 g (0.15 mmol) of [Ru(η^6^-*p*-cymene)(μ-Cl)Cl]_2_ and an equimolar amount of the appropriate organic ligand (L = pqcame (**9**); 4-Mepq (**10**); pq (**11**)) were dissolved in dry methanol (10 mL). The obtained orange solution was stirred for 18 h at room temperature and, after filtration, the volume of the solution was concentrated, and diethyl ether (10 mL) was added. The obtained orange precipitate was washed with diethyl ether (3 × 3 mL) and finally dried under vacuum to afford the final product.

#### Data for Complexes **9**–**11**

[Ru(η^6^-*p*-cymene)(**pqcame**)Cl][Ru(η^6^-*p*-cymene)Cl_3_] (**9**). Yield: (0.130 g, 95%). Found: C, 46.44; H, 4.44; N, 2.92. C_36_H_40_Cl_4_N_2_O_2_Ru_2_∙3H_2_O requires C, 46.46; H, 4.60; N, 3.20%. IR (KBr, *ν*_max_/cm^−1^): 3077 (w, *ν*(C–H)_arom_), 3046 (m, *ν*(C–H)_arom_), 2871 (w, *ν*_as_(C–H)_aliph_), 1725 (vs, *ν*_as_(C = O)), 1595 (w, *ν*(C = N)), 1479 (w, *ν*(C = C)), 1381 (w, *ν*(C = C)). UV-vis (CHCl_3_, 1.0 × 10^−4^ mol dm^−3^): λ_max_/nm 364 (ε/dm^3^ mol^−1^ cm^−1^ 24,700), 443 (8000). UV-vis (H_2_O, 1.0 × 10^−4^ mol dm^−3^): λ_max_/nm 358 (27,300), 416 (6700). ^1^H NMR (CDCl_3_, 400 MHz, 298K) *δ*_H_**/**ppm 9.84 (1H, d, *J* = 4.0, H8), 9.00 (1H, d, *J* = 8.0, H6′), 8.93 (1H, d, *J* = 8.0, H5), 8.67 (1H, s, H3), 8.32 (1H, d, *J* = 8.0, H3′), 8.04 (2H, m, H4′/H5′), 7.91 (1H, t, *J* = 8.0, H6), 6.33 (1H, d, *J* = 8.0, H-cym_ar_), 6.23 (1H, d, *J* = 4.0, H-cym_ar_), 6.00 (1H, d, *J* = 4.0, H-cym_ar_), 5.77 (1H, d, *J* = 8.0, H-cym_ar_), 5.48 (2H, t, *J* = 4.0, H-cym_ar_′), 5.35 (1H, d, *J* = 4.0, H-cym_ar_′), 5.24 (1H, d, *J* = 4.0, H-cym_ar_′), 4.17 (3H, s, COOC*H*_3_), 3.20 (0.60H, sept, *J* = 8.0, CH_A_-(CH_3_)_2_), 2.94 (0.40H, sept, *J* = 8.0, CH_Β_-(CH_3_)_2_), 2.40 (1H, sept, *J* = 8.0, CH’-(CH_3_’)_2_), 2.31 (1.8H, s, CH_3A_-cym), 2.18 (4.2H, s, CH_3_*’*-cym and CH_3Β_-cym), 1.38 (3H, d, *J* = 4.0, CH-(CH_3A_)_2_), 1.30 (3H, d, *J* = 4.0, CH-(CH_3Β_)_2_), 0.96 (3H, d, *J* = 8.0, CH’-(CH_3_’)_2_), 0.91 (3H, d, *J* = 8.0, CH’-(CH_3_’)_2_) (assignment marked with a prime refers to the *p*-cymene protons of the counter anion [Ru(η^6^-p-cymene)Cl_3_]**^−^**). ^13^C{^1^H} NMR (CDCl_3_, 100 MHz, 298K) *δ_C_*(ppm) 164.53 (CO), 159.23 (C2′), 156.32 (C2), 153.21 (C4′), 150.56 (C6′), 139.44 (C8), 137.88 (C8a), 132.30 (C4), 130.78 (C3′), 130.32 (C5′), 126.75 (C4a), 126.10 (C6), 125.43 (C7), 124.51 (C5), 119.54 (C3), 106.02 (C-C_cym-ar_), 103.89 (C′-C_cym-ar_), 100.71 (C-C_cym-ar_), 96.36 (C′-C_cym-ar_), 88.32 (C-C_cym-ar_), 87.01 (C-C_cym-ar_), 86.20 (C-C_cym-ar_), 86.11(C-C_cym-ar_), 81.83 (C′-C_cym-ar_), 81.31 (C′-C_cym-ar_), 80.56 (C′-C_cym-ar_), 79.71 (C′-C_cym-ar_), 53.71 (OCH_3_), 31.15 (*C*′H-(C′H_3_)_2_), 30.70 (*C_A_*H-(CH_3_)_2_), 30.64 (*C**_Β_***H-(CH_3_)_2_), 22.40 (2C, CH-(*C*H_3_)_2_), 22.15 (C′H-(*C*′H_3_)_2_), 21.62 (C′H-(*C*’H_3_)_2_), 18.92 (*C*_Β_H_3_-cym), 18.84 (*C*_A_H_3_-cym), 18.80 (*C*′H_3_-cym).

[Ru(η^6^-*p*-cymene)(**4-Mepq**)Cl][Ru(η^6^-*p*-cymene)Cl_3_] (**10**). Yield: (0.121 g, 92%). Found: C, 50.31; H, 5.04; N, 3.33. C_35_H_40_Cl_4_N_2_Ru_2_ requires C, 50.49; H, 4.84; N, 3.36%. IR (KBr, *ν*_max_/cm^−1^): 3061 (m, *ν*(C–H)_arom_, 3048 (m, *ν*(C–H)_arom_), 2920 (m, *ν*_as_(C–H)_aliph_), 1600 (s, *ν*(C = N)), 1486 (s, *ν*(C = C)), 1383 (m, *ν*(C = C)), 773 (m). UV-vis (CHCl**_3_**, 1.0 × 10^−4^ mol dm^−3^): λ_max_/nm 258 (ε/dm^3^ mol^−1^ cm^−1^ 30,800), 292 (27,300), 336 (16,400), 350(17,700), 414 (3600). UV-Vis (H_2_O, 1.0 × 10^−4^ mol dm^−3^): λ_max_/nm 253 (17,100), 286 (16,400), 331 (8600), 345 (11,300), 413 (1600). ^1^H NMR (CDCl_3_, 400 MHz, 298K) *δ*_H_**/**ppm 9.71 (1H, d, *J* = 4.0, H8), 8.79 (1H, d, *J =* 8.0, H6′), 8.32 (1H, d, *J* = 8.0, H5), 8.25 (1H, s, H3), 8.09 (1H, d, *J* = 8.0, H3′), 7.93 (1H, t, *J* = 4.0, H5′), 7.79 (2H, m, H7/H4′), 7.70 (1H, t, *J* = 8.0, H6), 6.16 (1H, d, *J* = 4.0, H-cym_ar_), 6.11 (1H, d, *J* = 8.0, H-cym_ar_), 5.78 (1H, d, *J* = 8.0, H-cym_ar_), 5.63 (1H, d, *J* = 4.0, H-cym_ar_), 5.47 (2H, t, *J* = 4.0, H-cym_ar_′), 5.32 (1H, d, *J* = 4.0, H-cym_ar_′), 5.24 (1H, d, *J* = 4.0, H-cym_ar_′), 3.20 (0.55H, sept, *J* = 4.0, CH_A_-(CH_3_)_2_), 2.89 (0.45H, sept, *J* = 4.0, CH_Β_-(CH_3_)_2_), 2.87 (3H, s, CH_3_ of 4-Mepq), 2.30 (1.65H, s, CH_3A_-cym), 2.24 (1H, sept, *J* = 8.0, C*H*’-(CH_3_’)_2_), 2.19 (3H, s, C*H*_3_′-cym), 2.15 (1.35H, s, CH_3B_-cym), 1.38 (3H, d, *J* = 4.0, CH-(CH_3A_)_2_), 1.27 (3H, d, *J* = 4.0, CH-(CH_3Β_)_2_), 0.84 (3H, d, *J* = 8.0, CH’-(CH_3_’)_2_), 0.79 (3H, d, *J* = 8.0, CH’-(CH_3_’)_2_) (assignment marked with a prime refers to the *p*-cymene protons of the counter anion [Ru(η^6^-p-cymene)Cl_3_]**^−^**). ^13^C{^1^H} NMR (CDCl_3_, 100 MHz, 298K) *δ*_C_**/**ppm 158.58 (C2′), 156.45 (C8), 154.45 (C2), 150.56 (C8a), 149.13 (C4), 139.83 (C7), 132.18 (C4a), 130.47 (C4′), 129.85 (C6), 129.84 (C5′), 129.54 (C6′), 125.64 (C5), 125.32 (C3′), 120.68 (C3), 105.27 (C-C_cym-ar_), 104.51 (C′-C_cym-ar_), 101.21 (C-C_cym-ar_), 97.19 (C′-C_cym-ar_), 96.75 (C-C_cym-ar_), 88.31 (C-C_cym-ar_), 86.14 (C-C_cym-ar_), 85.56 (C-C_cym-ar_), 82.23 (C′-C_cym-ar_), 81.74 (C′-C_cym-ar_), 80.99 (C′-C_cym-ar_), 80.22 (C′-C_cym-ar_), 31.37 (*C*_A_H-(CH_3_)_2_), 31.34 (*C*_B_H-(CH_3_)_2_), 31.16 (*C*′H-(C′H_3_)_2_), 31.07 (*C*′H-(C′H_3_)_2_), 22.84 (C′H-(*C*’H_3_)_2_), 22.69 (C′H-(*C*’H_3_)_2_, 22.58 (CH-(*C*H_3_)_2_), 22.07 (CH-(*C*H_3_)_2_), 19.79 (*C*′H_3_-cym), 19.35 (*C*_Β_H_3_-cym), 19.32 (*C*_A_H_3_-cym), 19.24 (*C*H_3_-quin).

[Ru(η^6^-*p*-cymene)(pq)Cl][Ru(η^6^-*p*-cymene)Cl_3_] (**11**). Yield: (0.120 g, 94%). Found: C, 50.28; H, 4.19; N, 3.37. C_34_H_38_Cl_4_N_2_Ru_2_ requires C, 50.49; H, 4.84; N, 3.36%. IR (KBr, *ν*_max_/cm^−1^): 3056 (s, *ν*(C–H)_arom_), 3031 (s, *ν*(C–H)_arom_), 2923 (s, *ν*_as_(C–H)_aliph_), 1388 (s, *ν*(C = C)). UV-vis (CHCl_3_, 1.0 × 10^−4^): λ_max_/nm 268 (ε/dm^3^ mol^−1^ cm^−1^ 14,700), 291 (14,300), 337 (8400), 351 (9300), 417 (1900). ^1^H NMR (CDCl_3_, 400 MHz, 298K) *δ*_H_**/**ppm 9.72 (1H, d, *J* = 4.0, H8), 8.79 (1H, d, *J* = 8.0, H6′), 8.52 (1H, d, *J* = 8.0, H3), 8.46 (2H, t, *J* = 8.0, H4/H5), 7.90 (3H, m, H6/7/H5′), 7.72 (1H, t, *J* = 8.0, H4′), 6.15 (2H, m, H-cym_ar_), 5.84 (2H, d, *J* = 8.0, H-cym_ar_), 5.69 (1H, d, *J* = 8.0, H-cym_ar_), 5.48 (1H, t, *J* = 8.0, H-cym_ar_′), 5.33 (1H, d, *J* = 8.0, H-cym_ar_′), 5.24 (1H, d, *J* = 8.0, H-cym_ar_′), 3.20 (0.55H, sept, *J* = 4.0, CH_A_-(CH_3_)_2_), 2.92 (0.45H, sept, *J* = 4.0, C*H*′-(CH_3_’)_2_), 2.30 (4.65H, m, CH_3A_-cym and C*H*_3_′-cym), 2.19 (3H, s, CH_3_’-cym), 2.15 (1.35H, s, CH_3_-cym), 1.37 (3H, d, *J* = 8.0, CH-(CH_3A_)_2_, 1.27 (d, 3H, *J* = 8.0, CH-(CH_3Β_)_2_), 0.89 (3H, d, *J* = 4.0, CH-(C*H*_3_)_2_), 0.83 (3H, d, *J* = 4.0, CH-(C*H*_3_)_2_) (assignment marked with a prime refers to the *p*-cymene protons of the counter anion [Ru(η^6^-p-cymene)Cl_3_]**^−^**). ^13^C NMR (CDCl_3_, 100 MHz, 298K) *δ*_C_**/**ppm 158.22 (C2′), 156.78 (C6′), 154.21 (C2), 149.51 (C8a), 140.80 (C5), 139.83 (C5′), 132.43 (C7), 129.65 (C6), 129.62 (C4′), 129.49 (C8), 129.46 (C4), 129.25 (C4a), 125.36 (C3’), 119.67 (C3), 105.42 (C-C_cym-ar_), 104.08 (C′-C_cym-ar_), 100.90 (C-C_cym-ar_), 96.49 (C′-C_cym-ar_), 88.00 (C-C_cym-ar_), 86.22 (C′-C_cym-ar_), 85.94 (C-C_cym-ar_), 85.47 (C-C_cym-ar_), 81.96 (C-C_cym-ar_), 81.44 (C′-C_cym-ar_), 80.68 (C′-C_cym-ar_), 79.88 (C′-C_cym-ar_), 31.14 (*C*_A_H-(CH_3_)_2_), 30.86 (*C*_Β_H-(CH_3_)_2_), 30.77 (*C*′H-(C′H_3_)_2_), 29.83 (*C*′H-(C′ H_3_)_2_), 22.54 (C′H-(*C*’H_3_)_2_), 22.52 (C′H-(*C*’H_3_)_2_), 22.28 (CH-(*C*H_3_)_2_), 21.68 (CH-(*C*H_3_)_2_), 19.06 (*C*_Β_H_3_-cym), 19.02 (*C*_A_H_3_-cym), 18.93 (*C*′H_3_-cym).

### 3.3. Single-Crystal X-Ray Structural Determination

The data collections of complexes **1**–**4, 6**, **8** and **9** were performed on a Bruker X8-KappaApexII diffractometer by using graphite monochromated MoKa radiation (*λ* = 0.7103 Å), generated by a sealed tube. For the data collection of **8-Mepq** and **4,6′-Me_2_pq**, a Bruker D8-Venture diffractometer with Cu Ka radiation (*κ* = 1.5418 Å) generated from an IlsHeliosOptics source was used. For **8,6′-Me_2_pq**, the collection of the data was performed on a Bruker APEX-II CCD diffractometer by using graphite monochromated Mo-*K*_α_ radiation (*λ* = 0.7103 Å), generated by a sealed tube. Both diffractometers were equipped with a low-temperature (100(2) K) device (Bruker Kryoflex I and Oxford Cryostream 800er series). Intensities were measured by fine-slicing x and u-scans and corrected for background, polarization, absorption and Lorentz effects. The structures were solved by intrinsic phasing methods implemented in Sheldrick’s XT program and refined anisotropically by the least-square procedure implemented in the SHELX program system [61,62]. Hydrogen atoms were included using the riding model on the bound carbon atoms. The structural data have been deposited with the Cambridge Crystallographic Data Centre (CCDC) with reference numbers 2,415,190 (**8-Mepq**), 2,415,191 (**4,6′-Me_2_pqca**), 2,415,192 (**8,6′-Me_2_pq**), 2,415,193 (**1**), 2,415,194 (**2**), 2,415,195 (**3**), 2,415,196 (**4**), 2,415,197 (**6**), 2,415,198 (**8**) and 2,415,199 (**9**), respectively. Crystallographic data and refinement conditions for **8-Mepq**, **4,6′-Me_2_pqca** and **8,6′-Me_2_pq** are summarized in Table S1 while the data for complexes **1-4** and **6, 8** and **9** are presented in Tables S2 and S3.

### 3.4. Catalytic Transfer Hydrogenation Experiment

A 100 mL two-necked flask was charged with the ruthenium catalyst (0.006 mmol), the appropriate ketone (2.22 mmol) and 15 mL of 2-propanol. The mixture was heated at 82 °C and the appropriate volume of a 0.015 M solution of KOH in 2-propanol solution was added (0.22 mmol) leading to a substrate/catalyst/base ratio of ∼400/1/40. Approximately 0.1 mL of the reaction mixture was sampled, the solvent was pumped down and, subsequently, the oily residue was analyzed by ^1^H NMR spectroscopy. The percentage conversions during the reaction were determined by means of ^1^H NMR spectroscopy. Upon completion of the reaction, the solution was cooled at room temperature and the solvent evaporated to dryness. Subsequently, hexane (15 mL) was added, and the residue was filtered through a small pad of dry SiO_2_. The resulting clear solution was evaporated to dryness affording the relevant alcohol. All experiments were performed twice.

## 4. Conclusions

In conclusion, a series of new substituted pyridine–quinoline ligands were synthesized as scaffolds to investigate their coordination chemistry. The single-crystal X-ray structures of the organic precursors and relevant ruthenium(II)-*p*-cymene complexes were determined for the first time. The new catalyst precursors can be easily prepared in high yields, as air stable solids, that can be stored for months without decomposition. The cationic ruthenium(II) *p*-cymene organometallic complexes successfully catalyze the transfer hydrogenation reaction of various ketone substrates under optimized reaction conditions. Particularly complexes 1 and 5 with methyl groups at positions 8 and 4 of the quinoline moiety, showed the best catalytic activity. Those with electron withdrawing groups (methyl ester) were less efficient, while the presence of the bulkier [Ru(η^6^-*p*-cymene)Cl_3_]^−^ complex counter anion seems to affect, to some extent, the catalytic activity exerted. An inner sphere mechanism can be proposed based on the detection of ruthenium(II)-H species.

## Figures and Tables

**Table 1:** Catalytic transfer hydrogenation of acetophenone using ruthenium(II) complexes **1**–**11**.

Entry	Complex *	Time (min)	Conversion (%) ^a^	ΤOΝ ^b^	TOF (h^−1^) ^c^
1	**1**	15	100	400	1600
2	**2**	15	85		
3	**2**	30	100	400	800
4	**3**	15	20		
5	**3**	60	100	400	400
6	**4**	15	90		
7	**4**	30	100	400	800
8	**5**	15	100	400	1600
9	**Ru-pqcame**	60	38		
10	**Ru-pqcame**	180	95	380	127
11	**6**	180	100	400	133
12	**7-Cl**	60	95	380	380
13	**8**	60	90	360	360
14	**9**	60	30		
15	**9**	180	90	360	120
16	**1** **0**	15	90	360	1440
17	**1** **1**	60	90	360	360

* Reaction conditions: ketone (2 mmol), catalyst (0.25 mol%), KOH (10 mmol%) (substrate/catalyst/base ratio = 400/1/40). Temperature: 82 °C, hydrogen donor: 2-propanol. ^a^ Conversion was monitored by ^1^H NMR and are the average of two experiments. ^b^ TON (Turnover number) = moles of product per moles of ruthenium catalyst according to ^1^H NMR. ^c^ TOF = TON/h (after completion of the reaction). Standard deviation (SD) = ±1.0%. For **2**, **4** and **6** only for final products.

**Table 2:** Catalytic transfer hydrogenation of substituted acetophenone derivatives and benzophenone, using catalysts **1**–**11**.

Entry	Substrate *	Complex	Time (min)	Conversion (%) ^a^	TON ^b^	TOF (h^−1^) ^c^
1	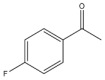	**1**	15	99	396	1584
2		**2**	30	95	380	760
3		**3**	60	70	280	280
4		**4**	30	99	392	792
5		**5**	15	95	380	1520
6		**Ru-pqcame**	180	85	340	113
7		**6**	180	87	348	116
8		**7** **-Cl**	60	92	368	388
9		**8**	60	82	328	328
10		**9**	180	85	340	113
11		**1** **0**	60	88	352	352
12		**1** **1**	60	83	332	332
13	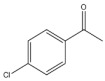	**1**	15	99	396	1584
14		**2**	30	98	392	784
15		**3**	60	90	360	360
16		**4**	30	98	392	784
17		**5**	15	98	392	1568
18		**Ru-pqcame**	180	95	380	127
19		**6**	180	98	392	131
20		**7** **-Cl**	60	95	380	380
21		**8**	60	90	360	360
22		**9**	180	90	360	120
23		**1** **0**	60	97	388	388
24		**1** **1**	60	82	328	328
25	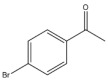	**1**	30	66	264	528
26		**2**	30	72	288	576
27		**3**	60	55	220	220
28		**4**	30	75	300	600
29		**5**	30	72	288	676
30		**Ru-pqcame**	180	72	288	96
31		**6**	180	88	336	117
32		**7** **-Cl**	60	65	260	260
33		**8**	60	66	264	264
34		**9**	180	60	240	80
35		**1** **0**	60	73	292	292
36		**1** **1**	60	66	264	264
37	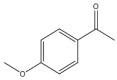	**1**	30	89	356	712
38		**2**	30	85	340	680
39		**3**	60	65	260	260
40		**4**	30	96	386	772
41		**5**	30	96	384	768
42		**Ru-pqcame**	180	78	312	104
43		**6**	180	94	376	126
44		**7** **-Cl**	60	75	300	300
45		**8**	60	70	380	280
46		**9**	180	75	300	100
47		**1** **0**	60	79	316	316
48		**1** **1**	60	78	312	312
49	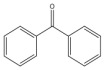	**1**	60	99	396	396
50		**2**	60	95	380	380
51		**3**	60	80	320	320
52		**4**	60	90	360	360
53		**5**	60	96	364	364
54		**Ru-pqcame**	180	85	340	113
55		**6**	180	82	328	109
56		**7** **-Cl**	60	96	384	384
57		**8**	60	88	352	352
58		**9**	180	82	328	109
59		**10**	60	88	352	352
60		**1** **1**	60	99	396	396

* Reaction conditions: ketone (2 mmol), catalyst (0.25 mmol %), KOH (10 mmol %). Temperature: 82 °C, hydrogen donor: 2-propanol; ^a^ Conversion was monitored by ^1^H NMR and are the average of two experiments. ^b^ TON (Turnover number) = moles of product per mole of ruthenium catalyst according to ^1^H NMR. ^c^ TOF = TON/h (after completion of the reaction). SD = ±1.0%.

## Data Availability

The data presented in this study are available on request from the corresponding author.

## Supplementary Materials

The following supporting information can be downloaded at https://www.mdpi.com/article/10.3390/molecules30142945/s1. Figures S1–S12: NMR spectra of the ligand precursors 8-Mepq, 6′-Mepq, 8,6′-Me_2_pq, 4,6′-Me_2_pq and pq in CDCl_3_; Figures S13–S15: ESI-HRMS of 8-Mepq, 6′-Mepq and 8,6′-Me_2_pq in methanol; Figures S16–S18: Intermolecular interactions in the unit cell of 8-Mepq, 4,6′-Me_2_pq and 8,6′-Me_2_pq; Figures S19–S25: FT-IR spectra of complexes **1**–**6**, **8**; Figures S26–S47: NMR spectra of complexes **1**–**5**, Ru-pqcame, **6**–**8** in (CD_3_)_2_CO; Figures S48–S55: UV-vis spectra of complexes **1**–**6**, Ru-pqcame and 8 in acetone; Figures S56–S60: Intermolecular interactions in the unit cell of **1**–**4**, **6**; Figure S61: Molecular structure of cation of **8**; Table S1: Selected improved crystallographic data for **8**; Figure S62: Intermolecular interactions in the unit cell of **8**; Figures S63–S65: FT-IR spectra of complexes **9**–**11**; Figures S66–S74: NMR spectra of complexes **9**–**11** in CDCl_3_; Figures S75–S79: UV-vis spectra of complexes **9**–**11** in CHCl_3_ and in H_2_O; Figure S80: Intermolecular interactions in the unit cell of **9**; Figure S81: Conversion versus reaction time for acetophenone transfer hydrogenation by catalysts Ru-pqcame and **10** within 60 min; Figure S82: ^1^H NMR spectrum (CH_3_OD) of a sample (pine green solid) of **4**, showing the formation of Ru-H species; Tables S2–S4: Crystal and refinement data for ligand precursors 8-Mepq, 4,6′-Me_2_pqca, 8,6′- Me_2_pqca and the metal complexes **1**–**4**, **7**, **9** and **10**.
