# Supplementary material for: Pyridine–Quinoline and Biquinoline-Based Ruthenium p-Cymene Complexes as Efficient Catalysts for Transfer Hydrogenation Studies: Synthesis and Structural Characterization
Source: Molecules. 2025 Jul 11;30(14):2945. doi: 10.3390/molecules30142945 (PMC12300421; doi:10.3390/molecules30142945)
Supplement: Supplementary file 1 [file molecules-30-02945-s001.zip › molecules-3713836-supplementary.pdf]

# Supplementary material

**For**

# Pyridine–Quinoline and Biquinoline–based Ruthenium *p*-cymene complexes as efficient catalysts for transfer hydrogenation studies: Synthesis and Structural Characterization

**Nikolaos Zacharopoulos[1], Gregor Schnakenburg[2], Eleni I. Panagopoulou[3], Nikolaos S. Thomaidis[3] and Athanassios I. Philippopoulos[1,*]**

[1] Laboratory of Inorganic Chemistry, Department of Chemistry, National and Kapodistrian University of Athens, Panepistimiopolis Zografou 15771, Athens, Greece.

[2] Institut für Anorganische Chemie, Rheinische Friedrich-Wilhelms-Universität Bonn, Gerhard-Domagk-Straße 1, D-53121 Bonn, Germany.

[3] Laboratory of Analytical Chemistry, Department of Chemistry, National and Kapodistrian University of Athens, Panepistimiopolis Zografou, 15771 Athens, Greece.

**Correspondence**

Athanassios. I. Philippopoulos, Laboratory of Inorganic Chemistry, Department of Chemistry, National and Kapodistrian University of Athens, Panepistimiopolis Zografou 15771, Athens, Greece. E-mail: atphilip@chem.uoa.gr

# Table of Contents

**Entry**


1) Figure S1 — $^{1}$H NMR spectrum of **8-Mepq** in $CDCl_3$

2) Figure S2 — $^{13}C\{^{1}H\}$-NMR of **8-Mepq** in $CDCl_3$

3) Figure S3 — $^{1}$H-NMR spectrum of **6′-Mepq** in $CDCl_3$

4) Figure S4 — $^{13}C\{^{1}H\}$-NMR of **6′-Mepq** in $CDCl_3$

5) Figure S5 — $^{1}$H-NMR spectrum of **8,6′-Me$_2$pq** in $CDCl_3$

6) Figure S6 — $^{13}C\{^{1}H\}$-NMR spectrum of **8,6′-Me$_2$pq** in $CDCl_3$

7) Figure S7 — $^{1}$H-NMR spectrum of **4,6′-Me$_2$pq** in $CDCl_3$

8) Figure S8 — $^{1}H$-$^{1}H$ COSY spectrum of **4,6′-Me$_2$pq** in $CDCl_3$

9) Figure S9 — $^{13}C\{^{1}H\}$-NMR spectrum of **4,6′-Me$_2$pq** in $CDCl_3$

10) Figure S10 — HSQC spectrum of **4,6′-Me$_2$pq** in $CDCl_3$

11)Figure S11 — HMBC spectrum of **4,6′-Me$_2$pq** in $CDCl_3$

12) Figure S12 — $^{1}$H-NMR spectrum of **pq** in $CDCl_3$

13)Figure S13 — ESI-HRMS of **8-Mepq** in methanol

14) Figure S14 — ESI-HRMS of **6′-Mepq** in methanol

15) Figure S15 — ESI-HRMS of **8,6′-Me$_2$pq** in methanol

16) Figure S16 — Intermolecular interactions in the unit cell of **8-Mepq**

17) Figure S17 — Intermolecular interactions in the unit cell of **4,6′-Me$_2$pq**

18) Figure S18 — Intermolecular interactions in the unit cell of **8,6′-Me$_2$pq**

19) Figure S19 — FT-IR spectrum of **1**

20) Figure S20 — FT-IR spectrum of **2**

21) Figure S21 — FT-IR spectrum of **3**

22) Figure S22 — FT-IR spectrum of **4**

23) Figure S23 — FT-IR spectrum of **5**

24) Figure S24 — FT-IR spectrum of **6**

25) Figure S25 FT-IR spectrum of **8**

26) Figure S26 $^{1}$H-NMR spectrum of **1** in $(CD_3)_2CO$

27) Figure S27 $^{1}$H-$^{1}$H COSY spectrum of **1** in $(CD_3)_2CO$

28) Figure S28 $^{13}C\{^{1}H\}$-NMR spectrum of **1** in $(CD_3)_2CO$

29) Figure S29 $^{1}$H-$^{13}$C-HSQC spectrum of **1** in $(CD_3)_2CO$

30) Figure S30 $^{1}$H-NMR spectrum of **2** in $(CD_3)_2CO$

31) Figure S31 $^{1}$H-$^{1}$H COSY spectrum of **2** in $(CD_3)_2CO$

32) Figure S32 $^{13}C\{^{1}H\}$-NMR spectrum of **2** in $(CD_3)_2CO$

33) Figure S33 $^{1}$H-$^{13}$C-HSQC spectrum of **2** in $(CD_3)_2CO$

34) Figure S34 $^{1}$H-$^{1}$H COSY spectrum of **3** in $(CD_3)_2CO$

35) Figure S35 $^{1}$H-$^{1}$H COSY spectrum of **4** in $(CD_3)_2CO$

36) Figure S36 $^{13}C\{^{1}H\}$-NMR spectrum of **4** in $(CD_3)_2CO$

37) Figure S37 $^{1}$H-$^{13}$C-HSQC spectrum of **4** in $(CD_3)_2CO$

38) Figure S38 $^{1}$H-NMR spectrum of **5** in $(CD_3)_2CO$

39) Figure S39 $^{1}$H-$^{1}$H COSY spectrum of **5** in $(CD_3)_2CO$

40) Figure S40 $^{13}C\{^{1}H\}$-NMR spectrum of **5** in $(CD_3)_2CO$

41) Figure S41 $^{1}$H-$^{13}$C-HSQC spectrum of **5** in $(CD_3)_2CO$

42) Figure S42 $^{1}$H-NMR spectrum of **Ru-pqcame** in $(CD_3)_2CO$

43) Figure S43 $^{1}$H-NMR spectrum of **6** in $(CD_3)_2CO$

44) Figure S44 $^{13}C\{^{1}H\}$-NMR of **6** in $(CD_3)_2CO$

45) Figure S45 $^{1}$H-NMR spectrum of **6-Cl** in $CDCl_3$

46) Figure S46 $^{1}$H-NMR spectrum of **7-Cl** in $(CD_3)_2CO$

47) Figure S47 $^{1}$H-NMR spectrum of **8** in $(CD_3)_2CO$

48) Figure S48 UV-vis spectrum of **1** in acetone

49) Figure S49 UV-vis spectrum of **2** in acetone

50) Figure S50 UV-vis spectrum of **3** in acetone

51) Figure S51 UV-vis spectrum of **4** in acetone

52) Figure S52 UV-vis spectrum of **5** in acetone

53) Figure S53 UV-vis spectrum of **Ru-pqcame** in acetone

54) Figure S54 UV-vis spectrum of **6** in acetone

55) Figure S55 UV-vis spectrum of **8** in acetone

56) Figure S56 Intermolecular interactions in the unit cell of **1**

**57)** Figure S57 Intermolecular interactions in the unit cell of **4**

**58)** Figure S58 Intermolecular interactions in the unit cell of **2**

**59)** Figure S59 Intermolecular interactions in the unit cell of **6**

**60)** Figure S60 Intermolecular interactions in the unit cell of **3**

**61)** Figure S61 Molecular structure of the complex cation of **8**. Hydrogen atoms and the $PF_6^-$ anion are omitted for clarity. The ellipsoids were plotted at the 50% probability

**62)** Table S1 Selected improved crystallographic data for **8**

**63)** Figure S62 Intermolecular interactions in the unit cell of **8**

**63)** Figure S63 FT-IR spectrum of **9**

64) Figure S64 FT-IR spectrum of **10**

65) Figure S65 FT-IR spectrum of **11**

66) Figure S66 $^{1}H$-NMR spectrum of **9** in $CDCl_3$

67) Figure S67 $^{13}C\{^{1}H\}$-NMR spectrum of **9** in $CDCl_3$

68) Figure S68 $^{1}H$-$^{13}C$-HSQC spectrum of **9** in $CDCl_3$

69) Figure S69 $^{1}H$-$^{1}H$ COSY spectrum of **10** in $CDCl_3$

70) Figure S70 $^{13}C\{^{1}H\}$-NMR spectrum of **10** in $CDCl_3$

71) Figure S71 $^{1}H$-$^{13}C$-HSQC spectrum of **10** in $CDCl_3$

72) Figure S72 $^{1}H$-$^{1}H$ COSY spectrum of **11** in $CDCl_3$

73) Figure S73 $^{13}C\{^{1}H\}$-NMR spectrum of **11** in $CDCl_3$

74) Figure S74 $^{1}H$-$^{13}C$-HSQC spectrum of **11** in $CDCl_3$

75) Figure S75 UV-vis spectrum of **9** in $CHCl_3$

76) Figure S76 UV-vis spectrum of **9** in $H_2O$

77) Figure S77 UV-vis spectrum of **10** in $CHCl_3$

78) Figure S78 UV-vis spectrum of **10** in $H_2O$

79) Figure S79 UV-vis spectrum of **11** in $CHCl_3$

80) Figure S80 Intermolecular interactions in the single crystal of **9**

81) Figure S81 Conversion versus reaction time for acetophenone transfer hydrogenation by catalysts **Ru-pqcame** and **10** within 60 min

82) Figure S82 $^{1}H$ NMR spectrum ($CH_3OD$) of a sample of **4** showing the formation of Ru-H species

83) Table S2 Crystal and refinement data for **8-Mepq, 4,6′-Me$_2$pqca** and **8,6′-Me$_2$pqca**

84) Table S3 Crystal and refinement data for **1–4**

85) Table S4 Crystal and refinement data for **7, 9** and **10**

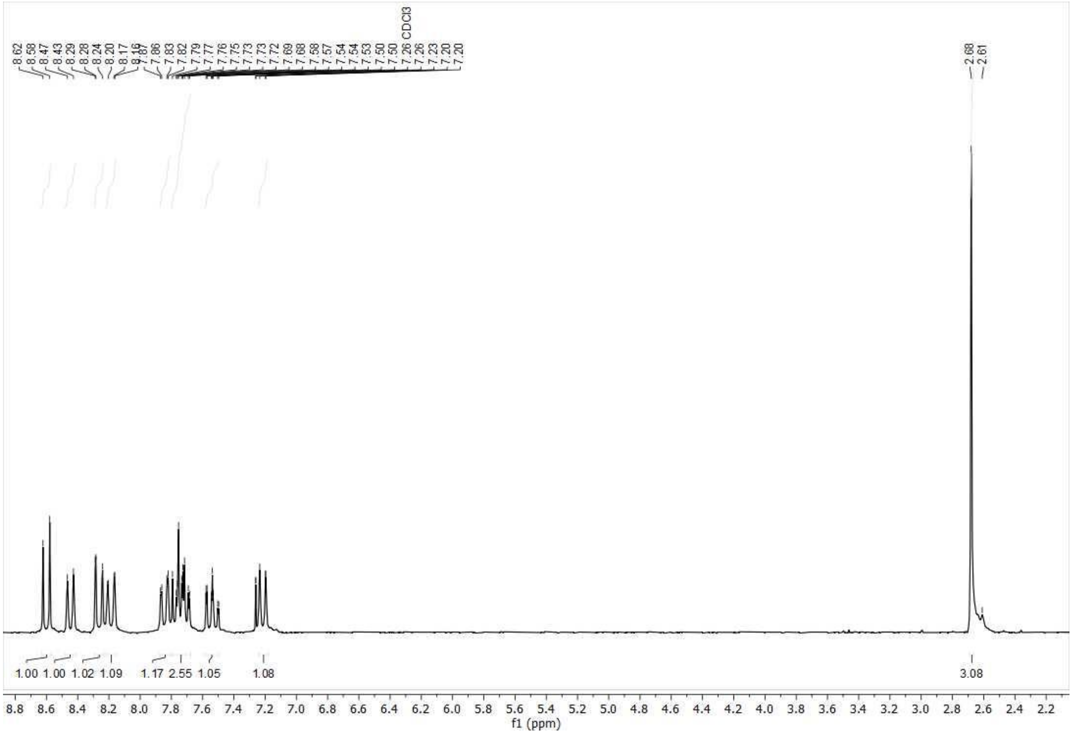


**Figure S1.** $^{1}$H-NMR of **8-Mepq** in $CDCl_3$

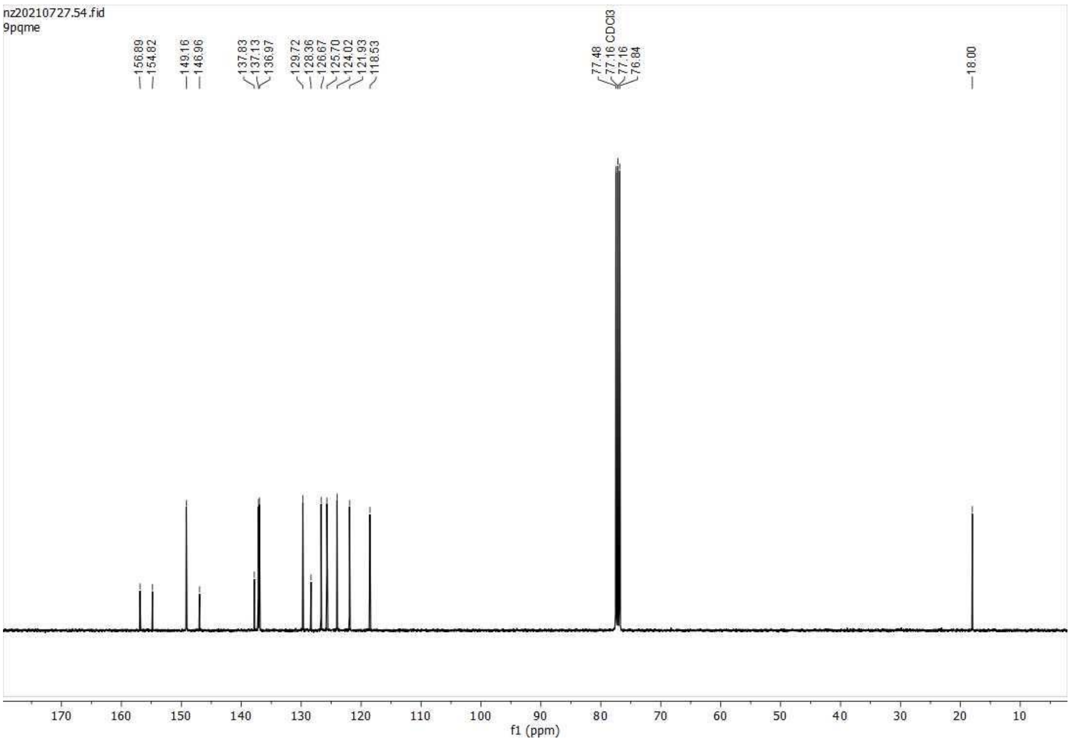


**Figure S2.** $^{13}$C{$^{1}$H}-NMR of **8-Mepq** in $CDCl_3$

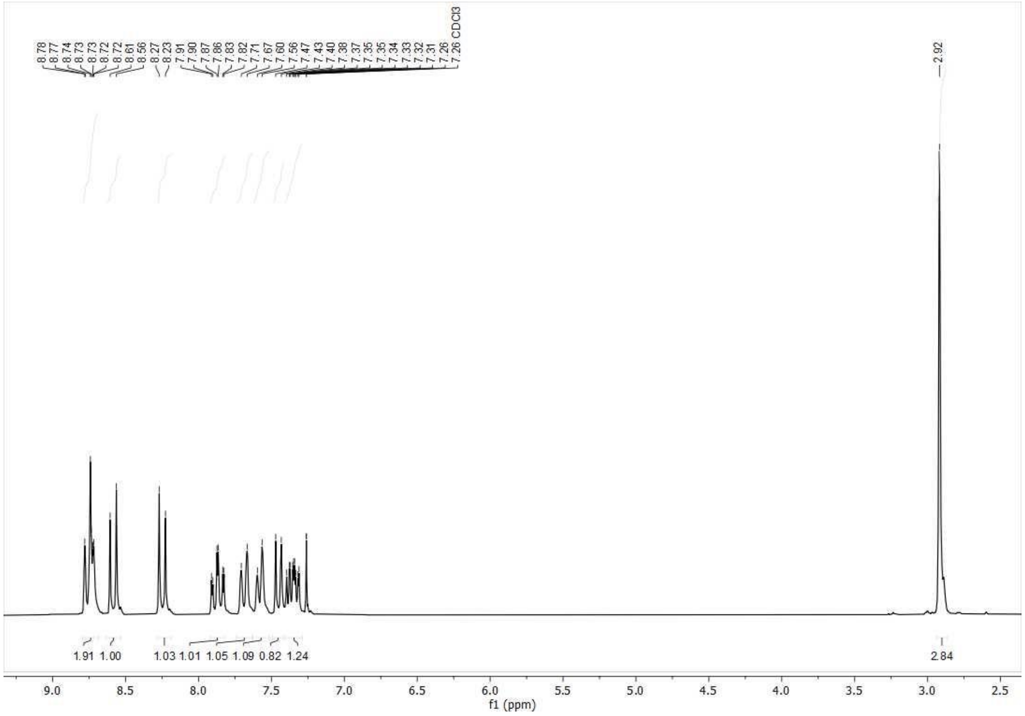

**Figure S3.** $^{1}H$-NMR spectrum of **6′-Mepq** in $CDCl_3$

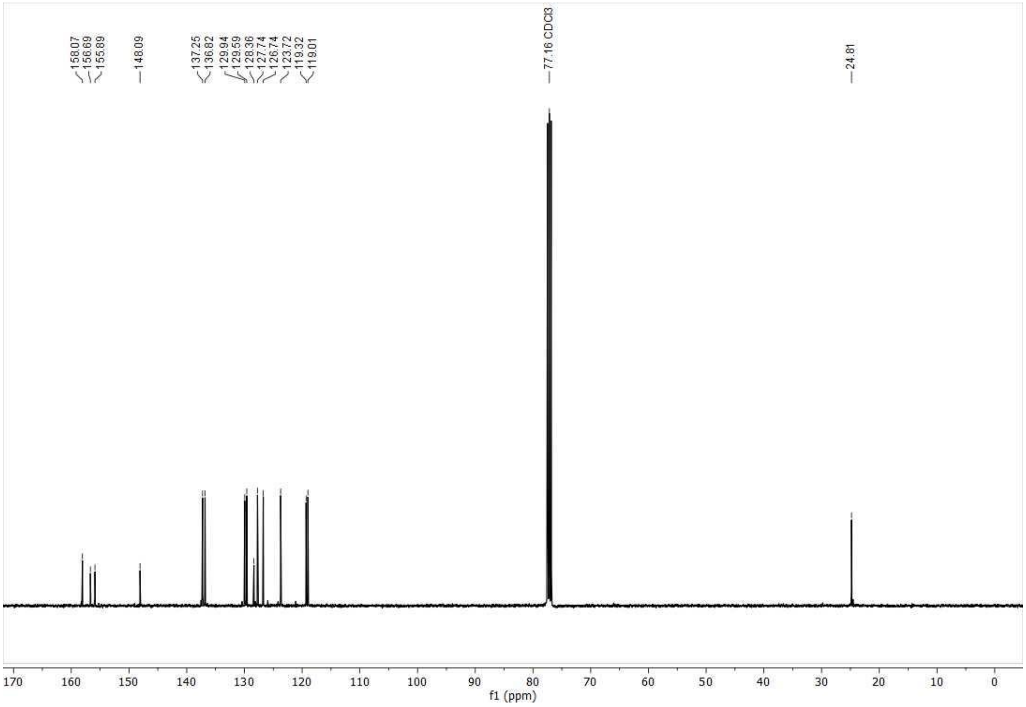

**Figure S4.** $^{13}C\{^{1}H\}$-NMR of **6′-Mepq** in $CDCl_3$

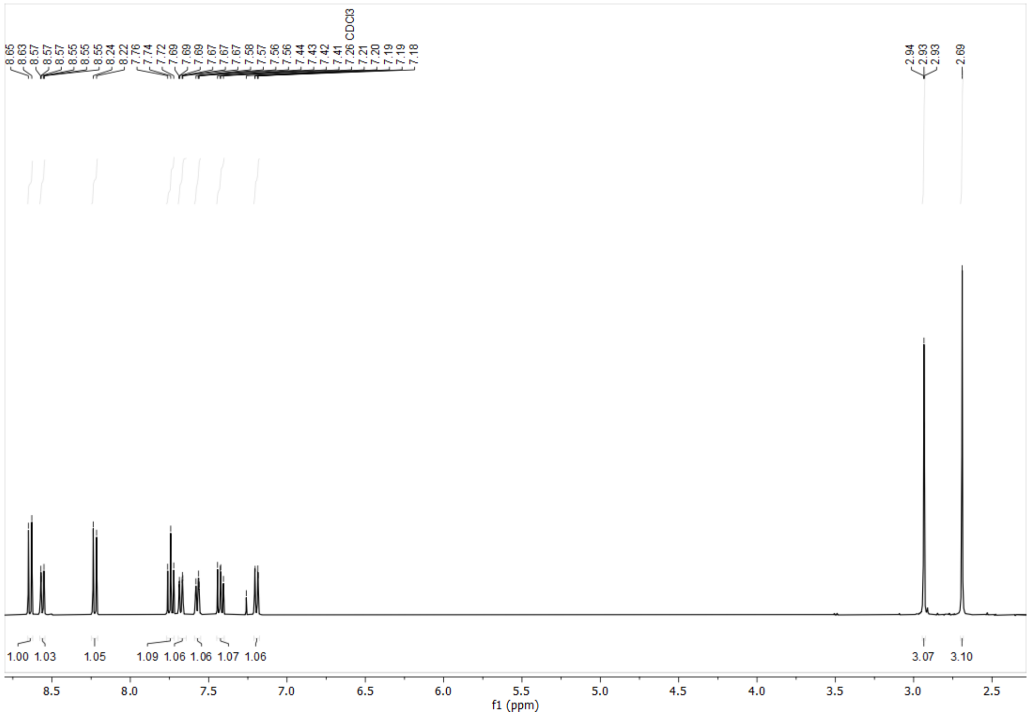

**Figure S5.** $^{1}H$-NMR spectrum of **8,6′-Me₂pq** in $CDCl_3$

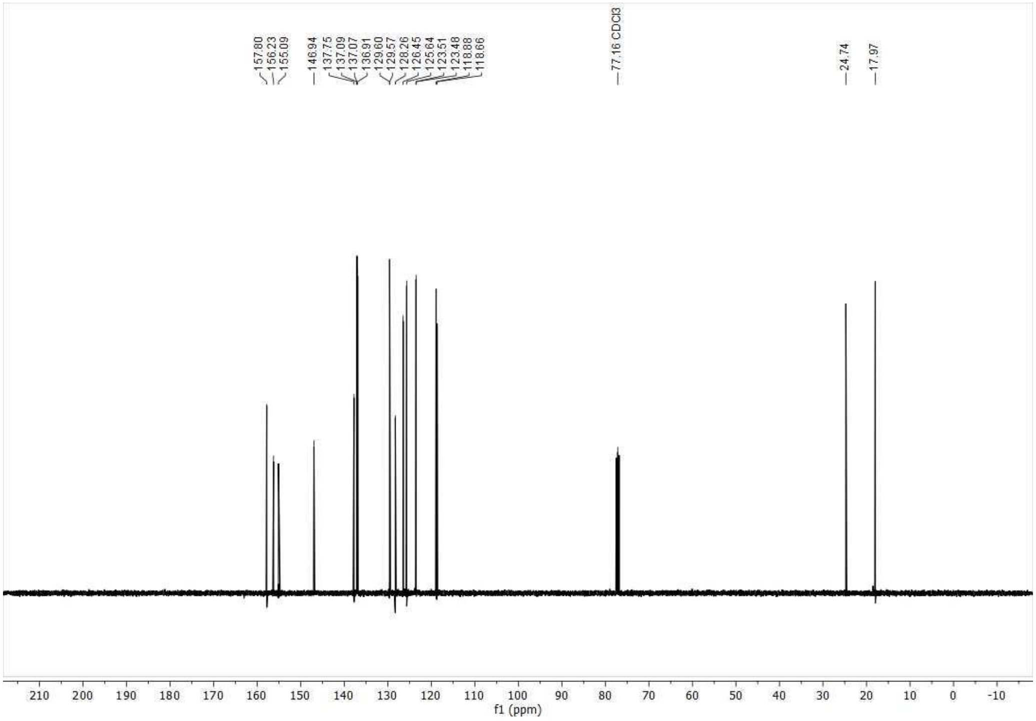

**Figure S6.** $^{13}C\{^{1}H\}$-NMR spectrum of **8,6′-Me₂pq** in $CDCl_3$

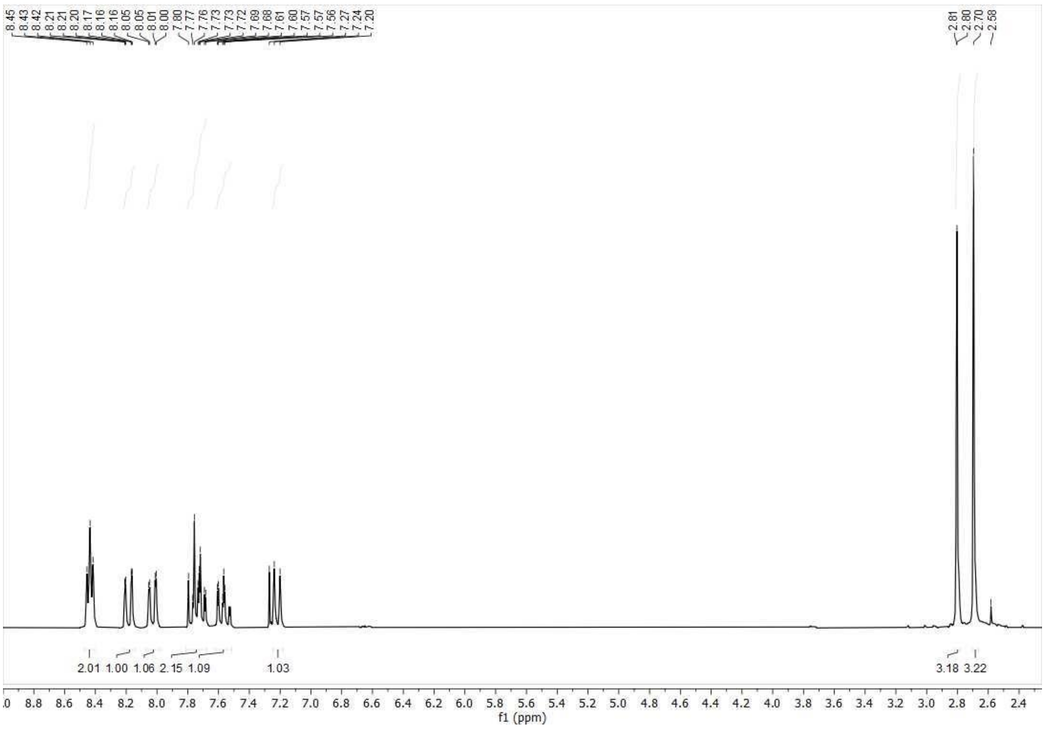

**Figure S7.** $^{1}$H-NMR spectrum of **4,6′-Me$_2$pq** in $CDCl_3$

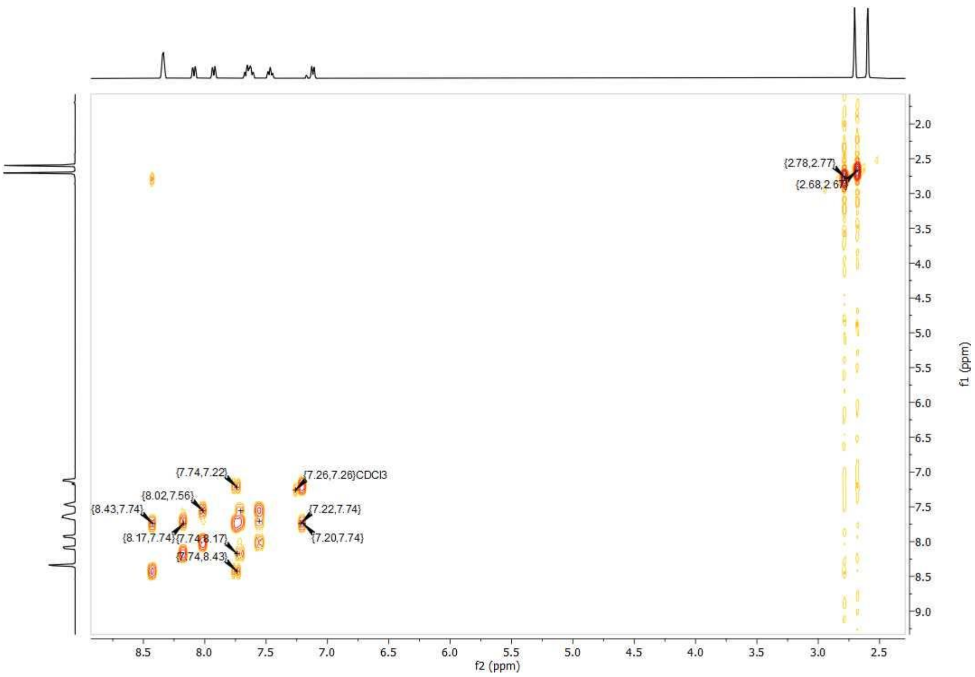


**Figure S8.** $^1$H-$^1$H COSY spectrum of **4,6′-Me$_2$pq** in $CDCl_3$

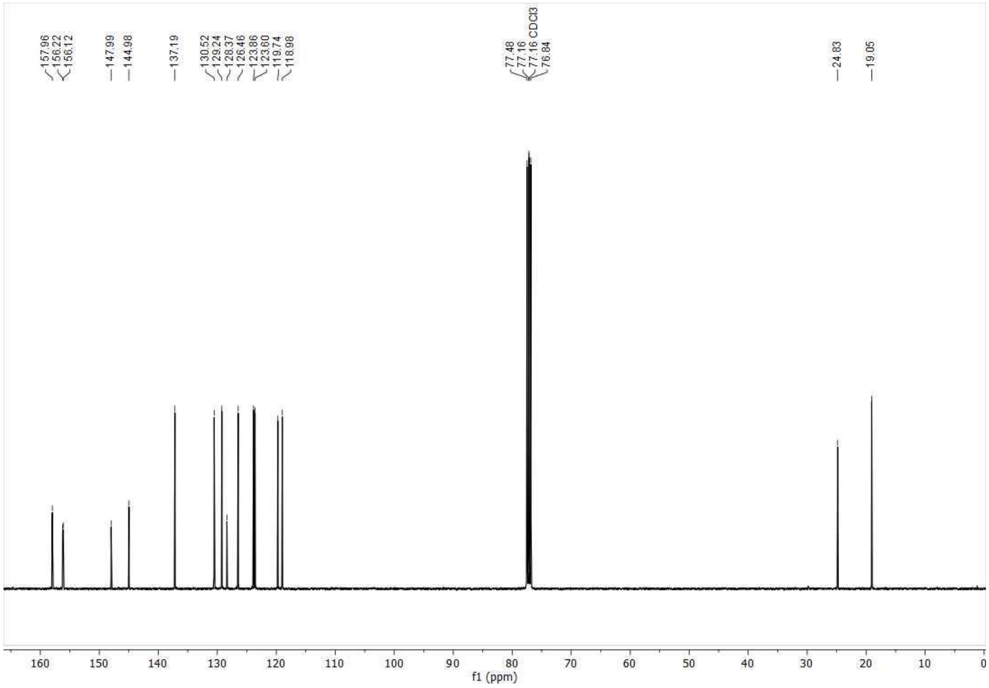


**Figure S9.** $^{13}$C{$^1$H}-NMR spectrum of **4,6′-Me$_2$pq** in $CDCl_3$

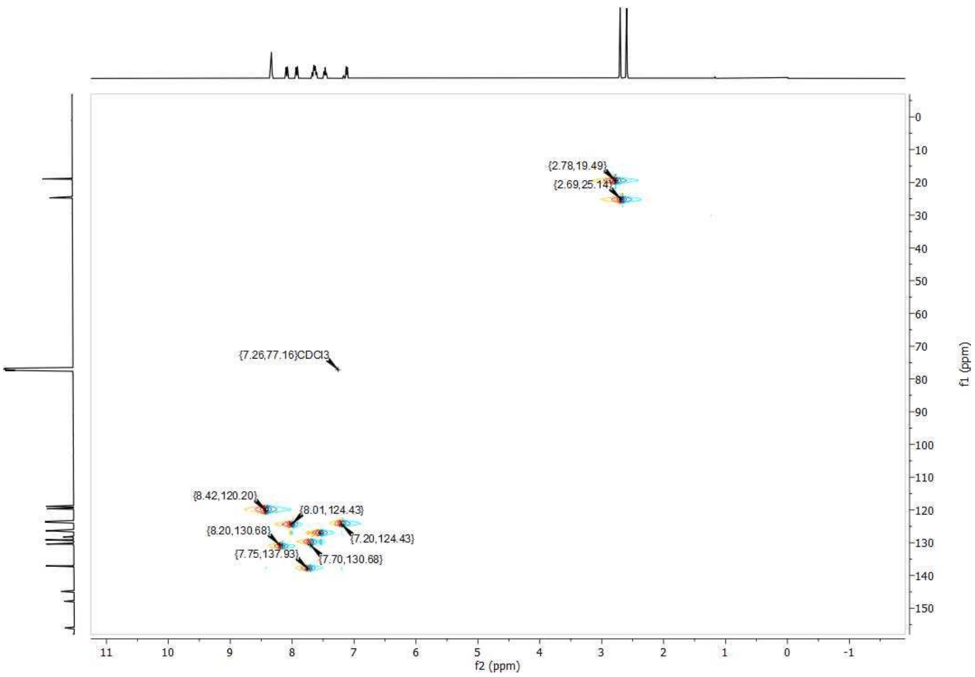


**Figure S10.** $^{1}H$-$^{13}C$-HSQC of **4,6′-Me$_2$pq** in $CDCl_3$

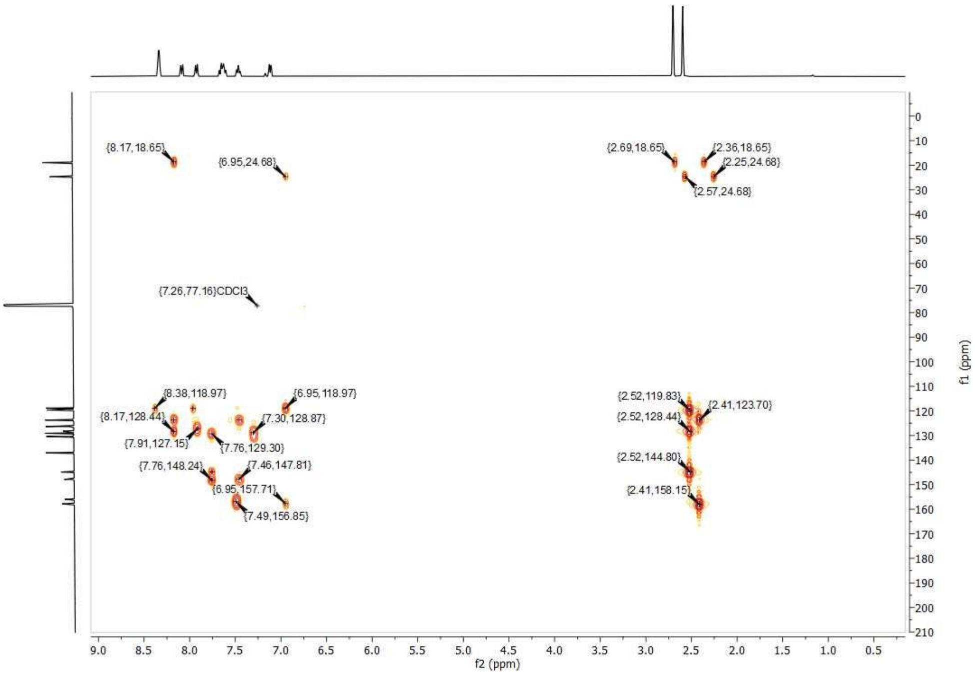


**Figure S11.** $^{1}H$-$^{13}C$-HMBC spectrum of **4,6′-Me$_2$pq** in $CDCl_3$

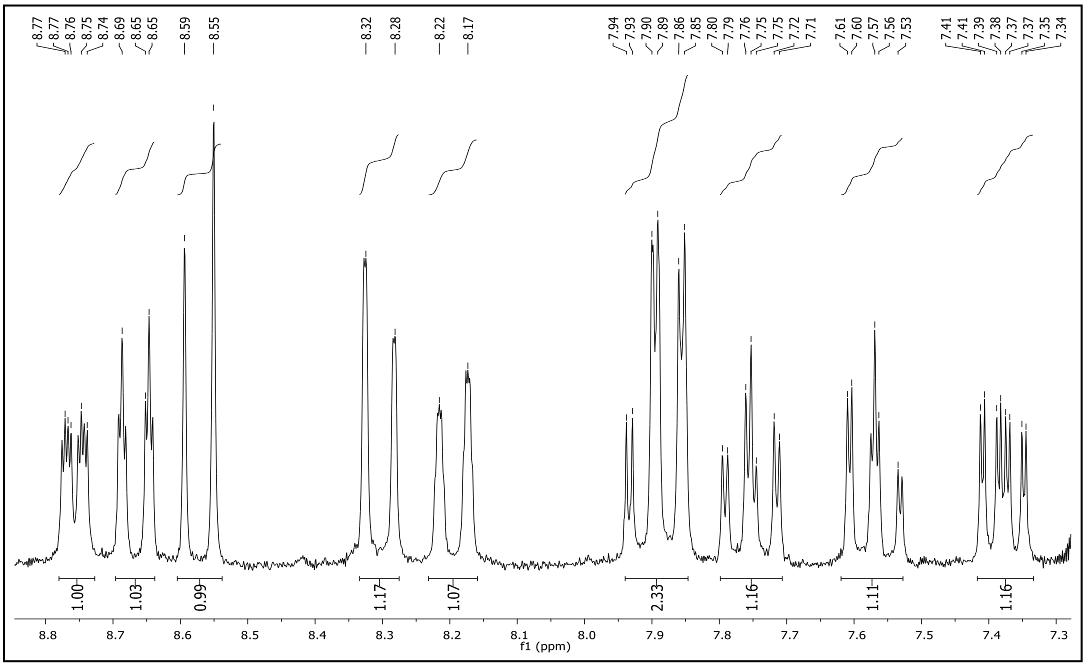


**Figure S12.** $^1$H NMR spectrum of **pq** in $CDCl_3$

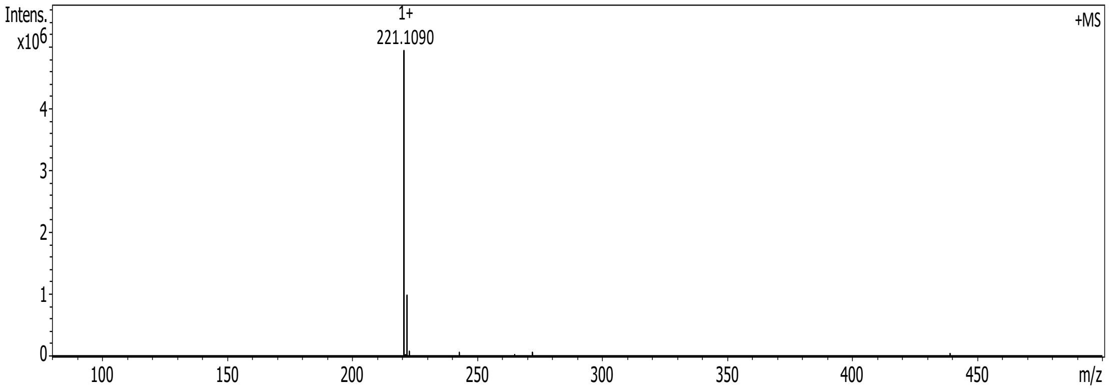


**Figure S13.** ESI-HRMS of **8-Mepq** in methanol

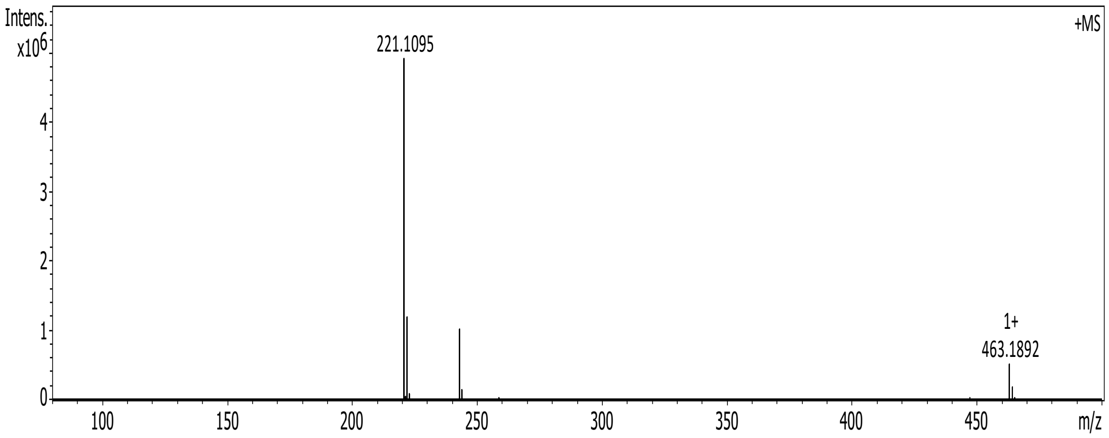


**Figure S14.** ESI-HRMS of **6′-Mepq** in methanol

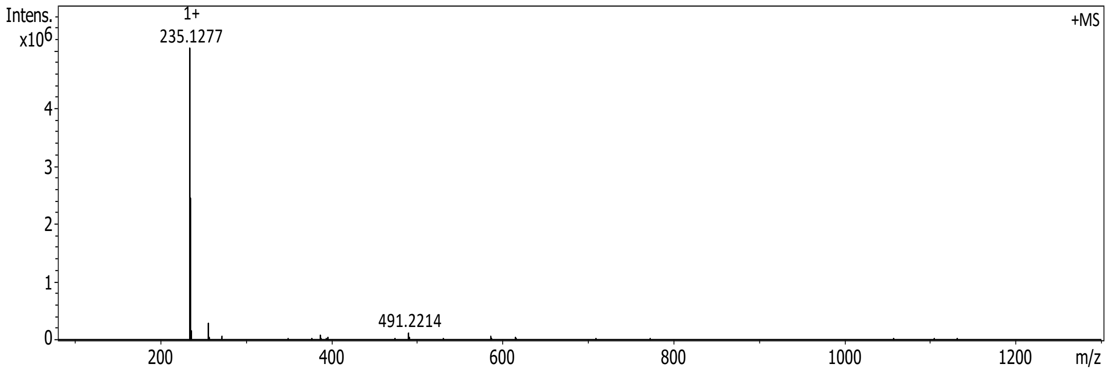


**(a)**

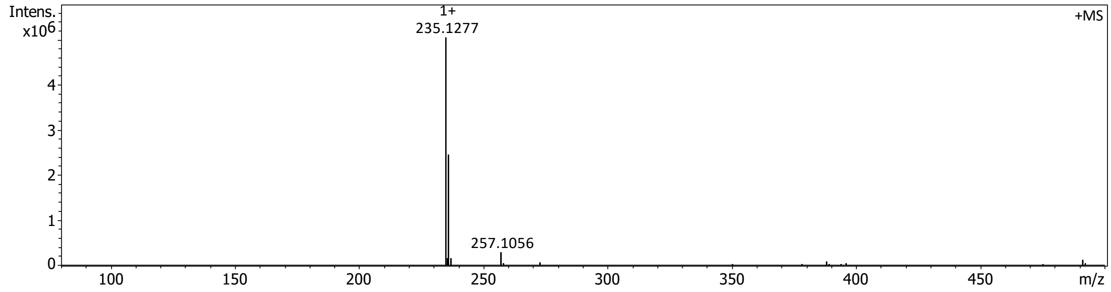


**(b)**

**Figure S15.** ESI-HRMS of **8,6′-Me$_2$pq** in methanol

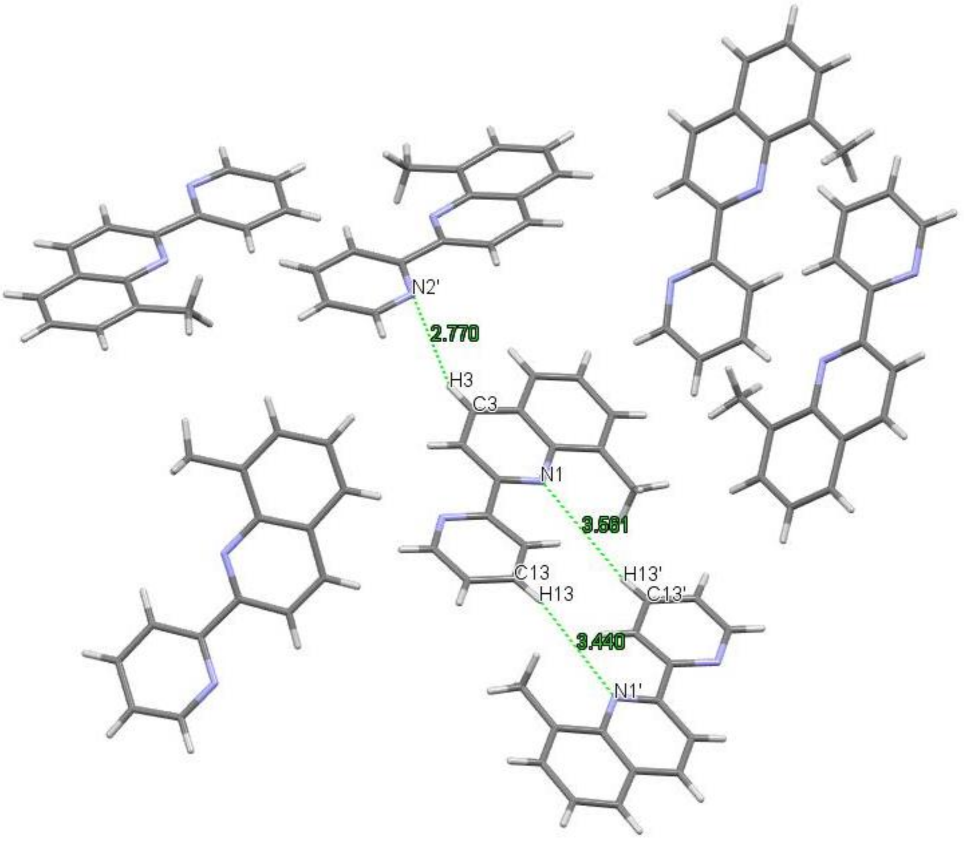


**Figure S16**. Intermolecular interactions in the single crystal of **8-Mepq**

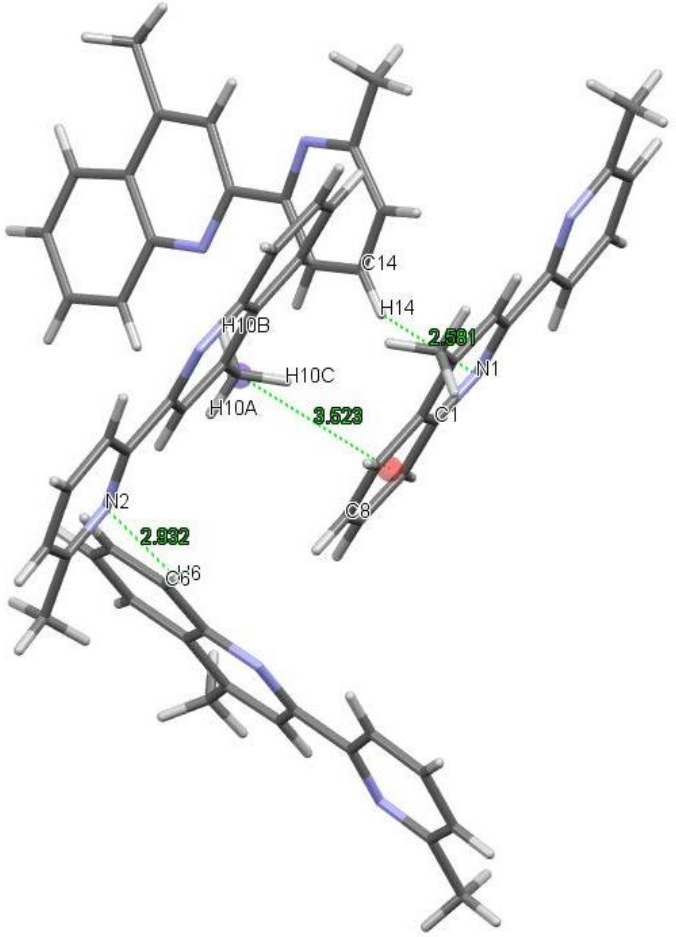


**Figure S17.** Intermolecular interactions in the single crystal of **4,6′-Me₂pq**

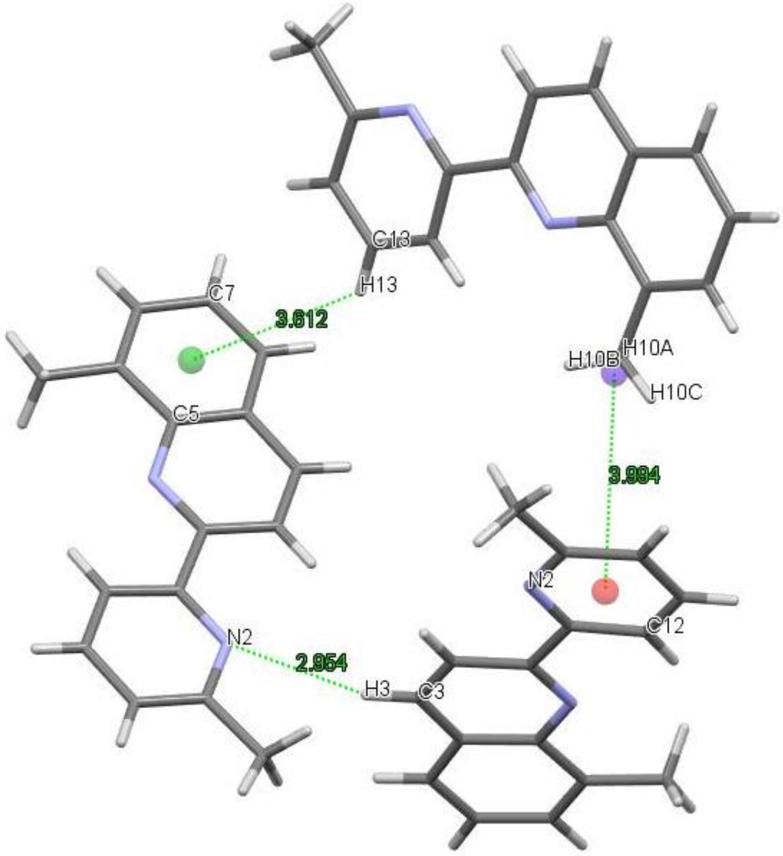


**Figure S18.** Intermolecular interactions in the single crystal of **8,6′-Me$_2$pq**

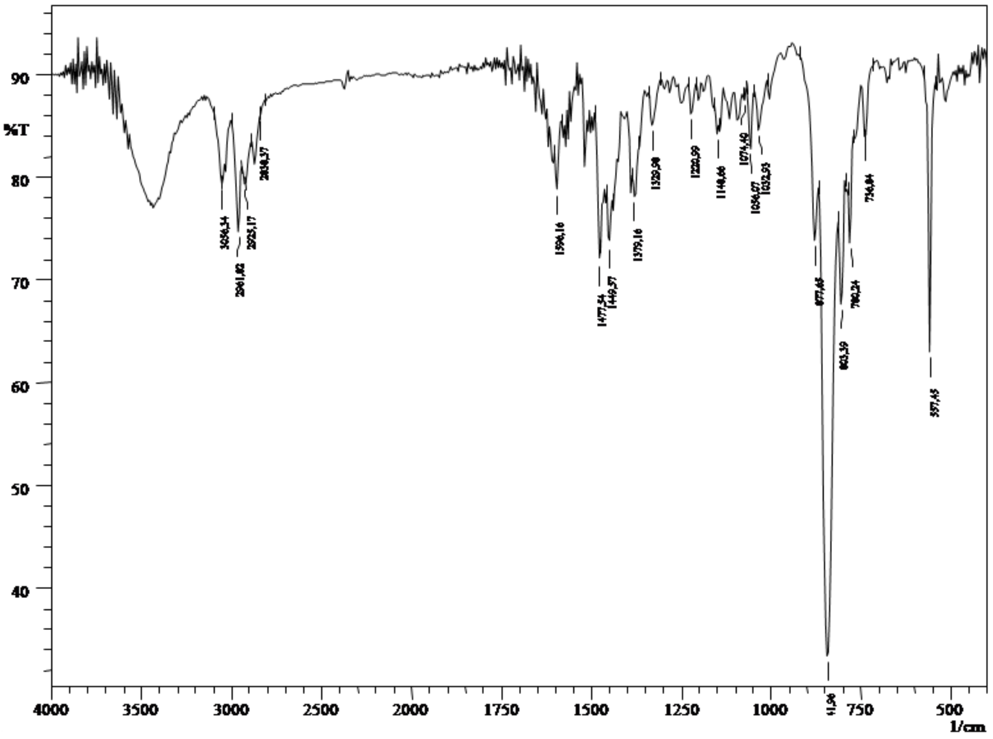


**Figure S19.** FT-IR spectrum of **1**

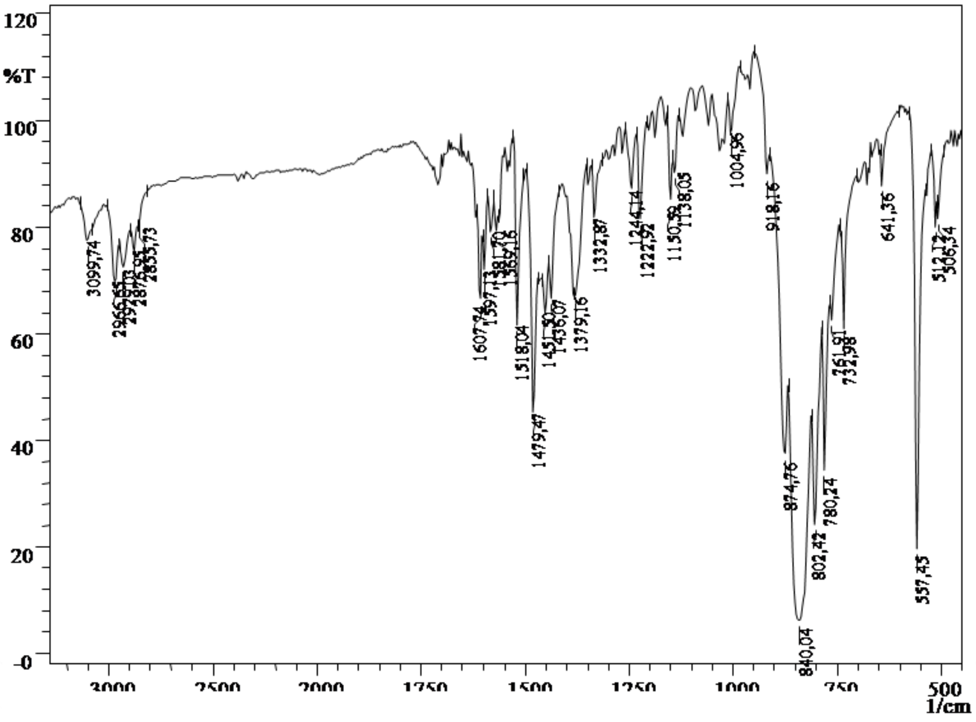


**Figure S20**. FT-IR spectrum of **2**

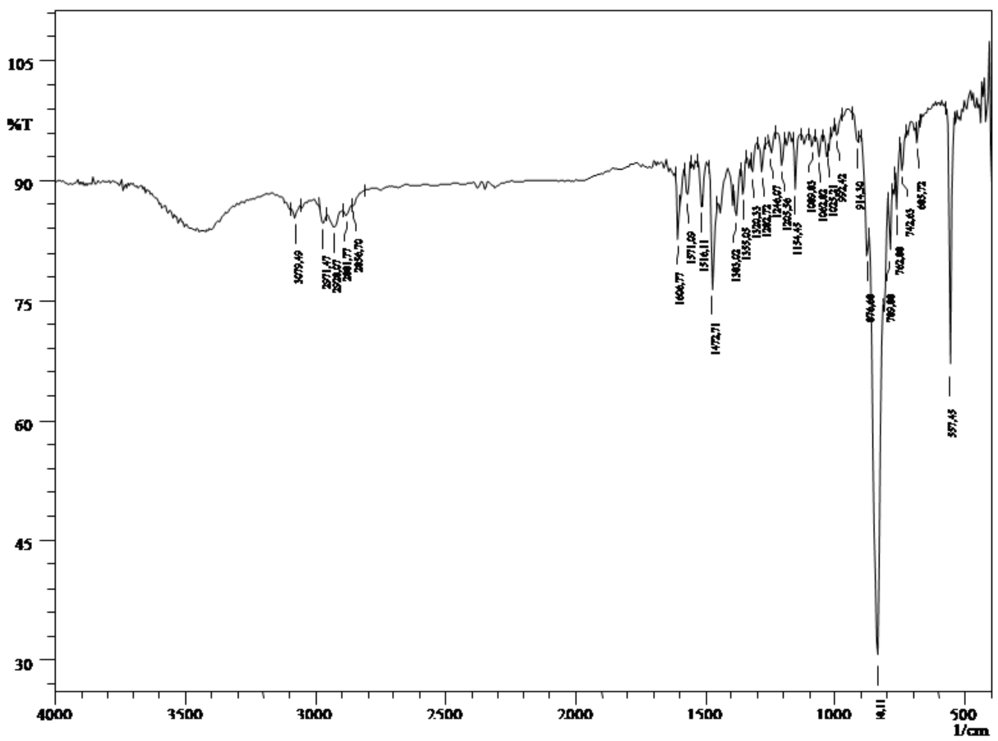


**Figure S21.** FT-IR spectrum of **3**

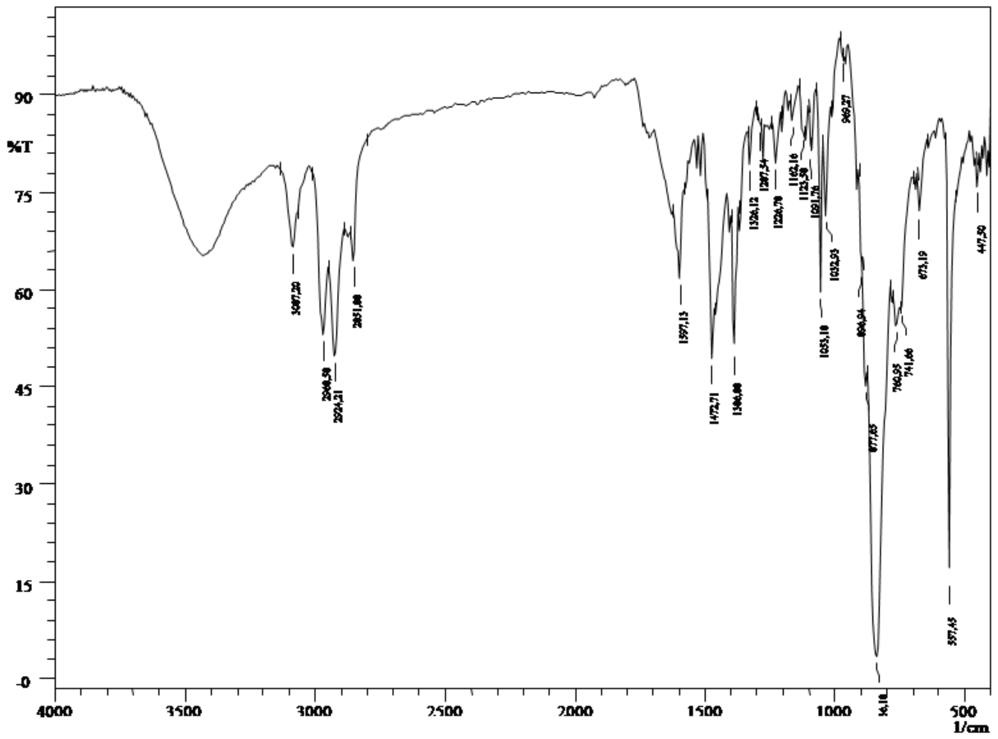


**Figure S22.** FT-IR spectrum of **4**

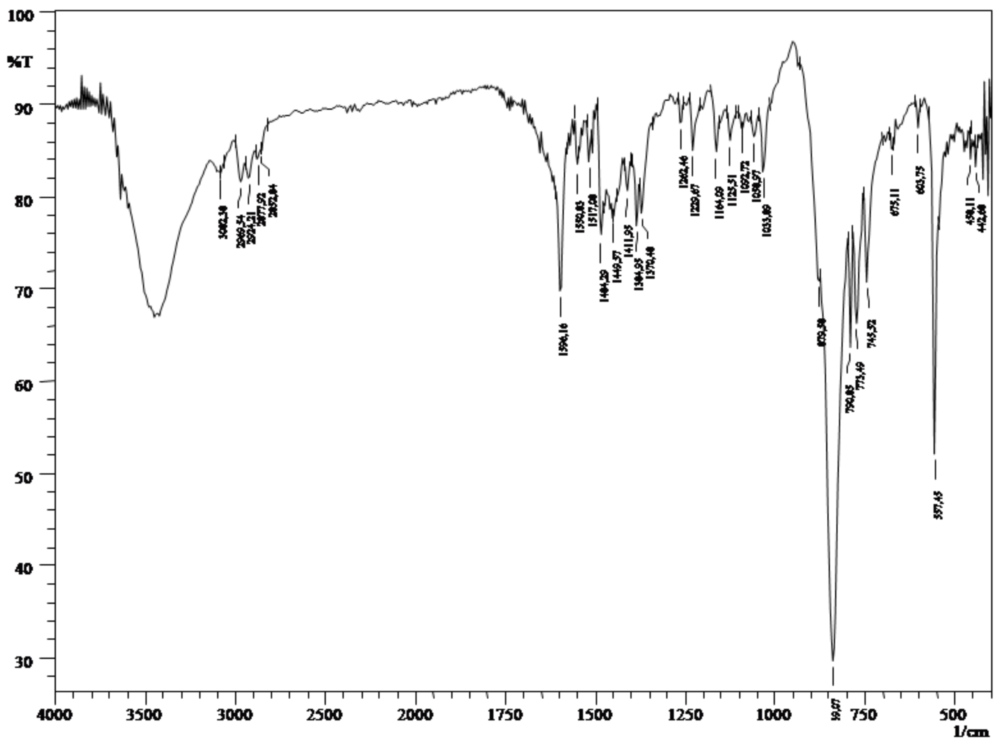


**Figure S23.** FT-IR spectrum of **5**

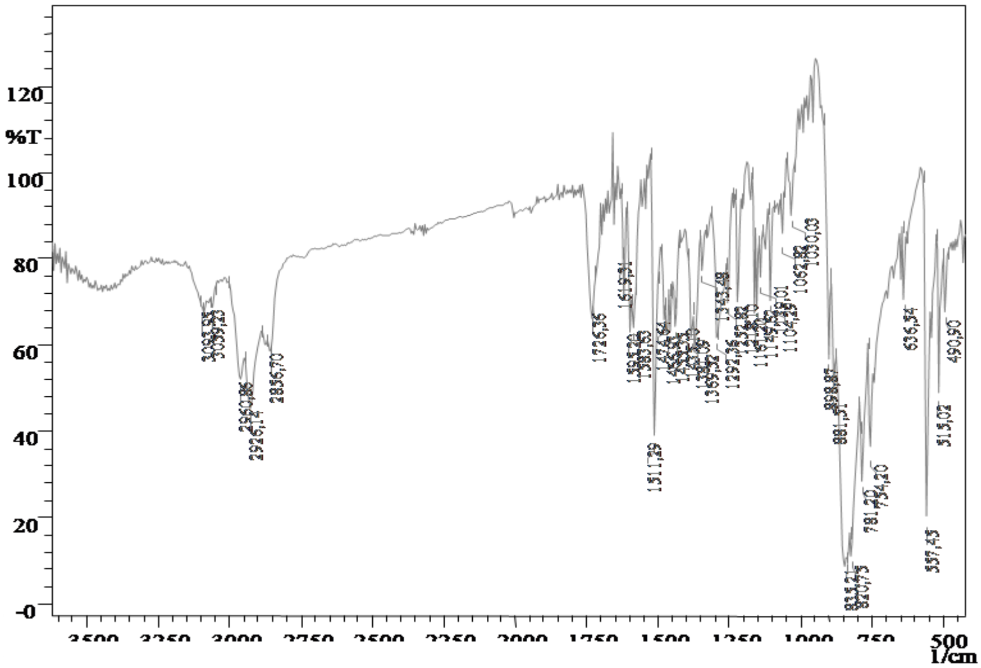


**Figure S24.** FT-IR spectrum of **6**

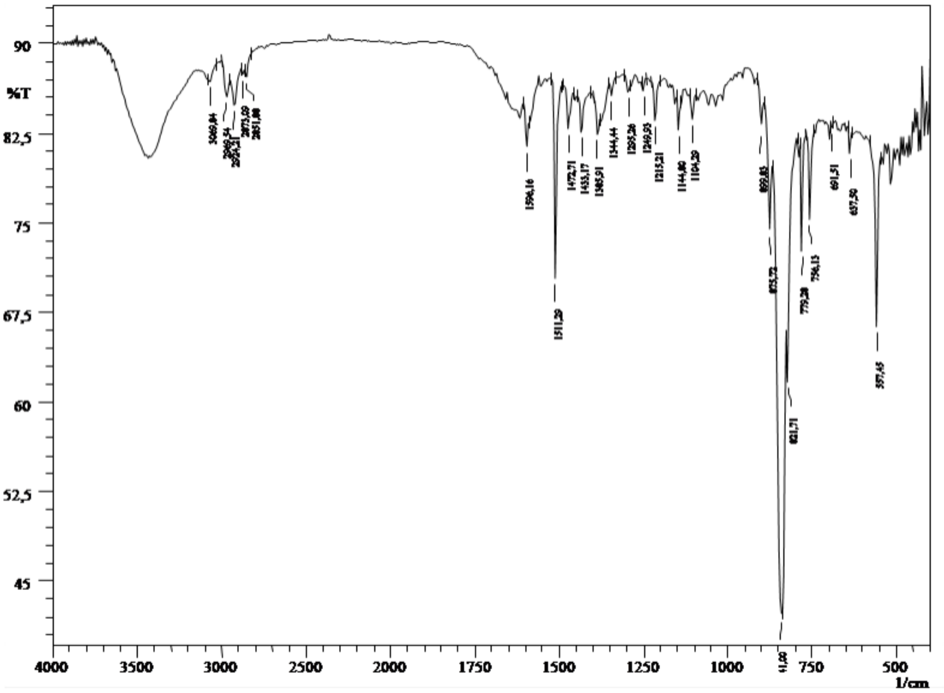


**Figure S25.** FT-IR spectrum of **8**

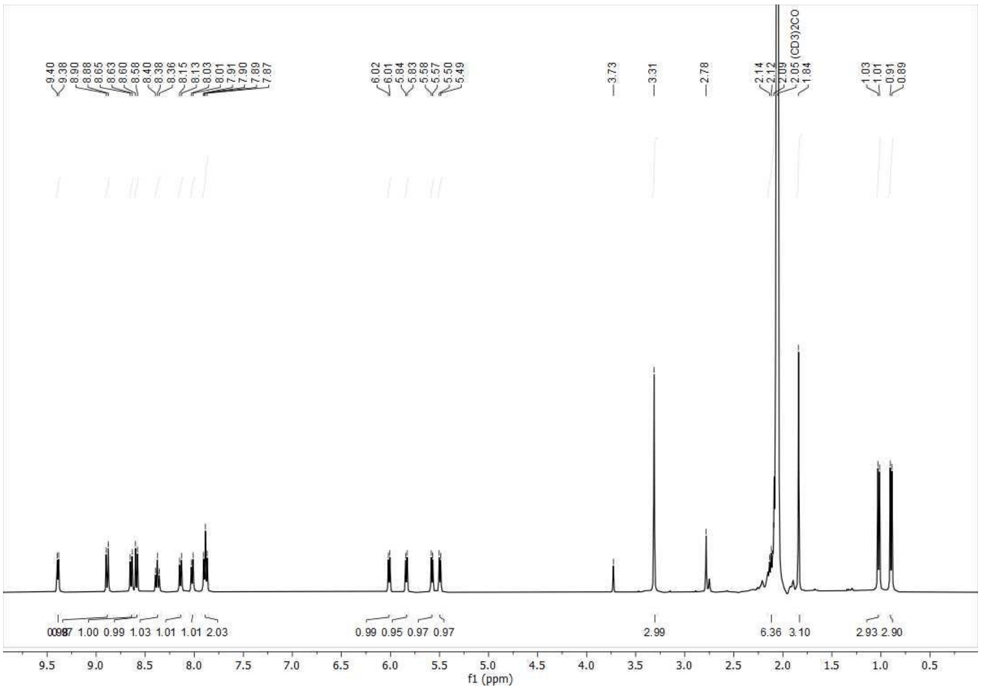


**Figure S26**. $^{1}$H-NMR spectrum of **1** in $(CD_3)_2CO$

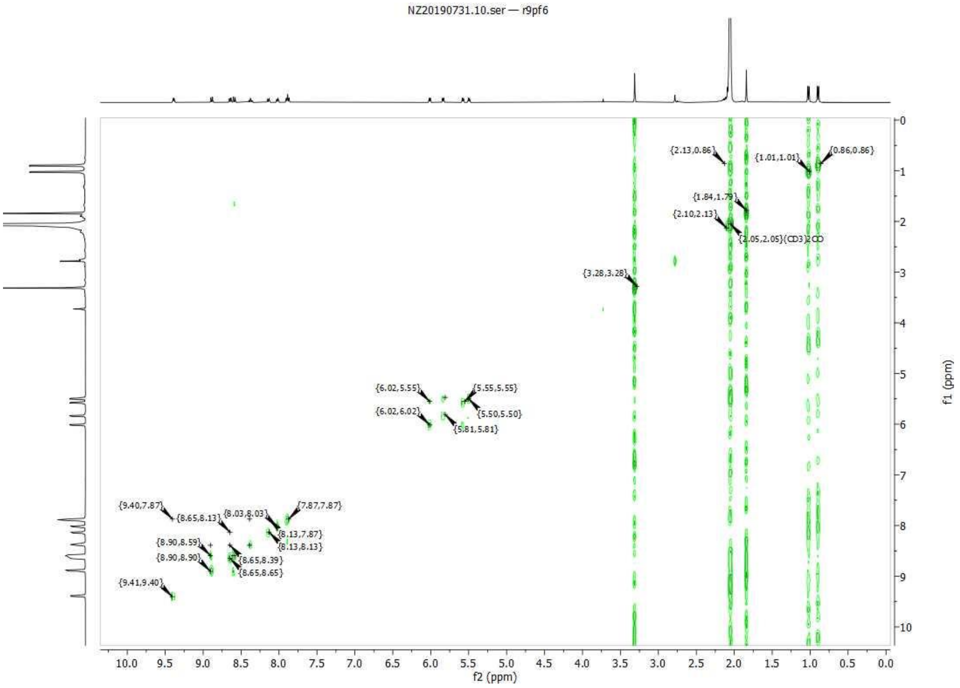

**Figure S27**. $^1H$-$^1H$ COSY spectrum of **1** in $(CD_3)_2CO$

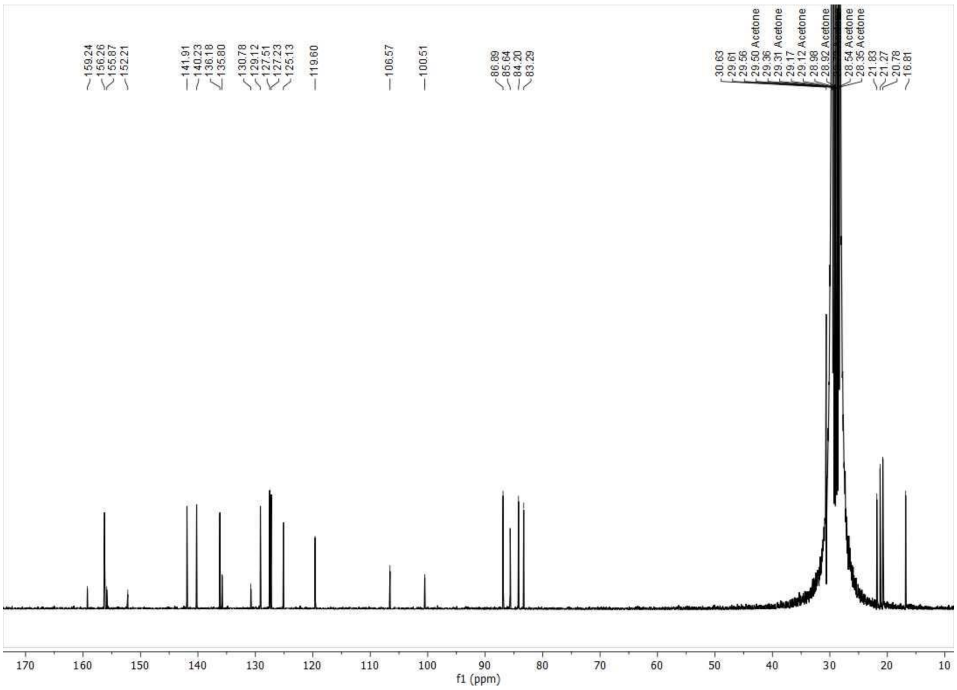

**Figure S28**. $^{13}C\{^1H\}$-NMR spectrum of **1** in $(CD_3)_2CO$

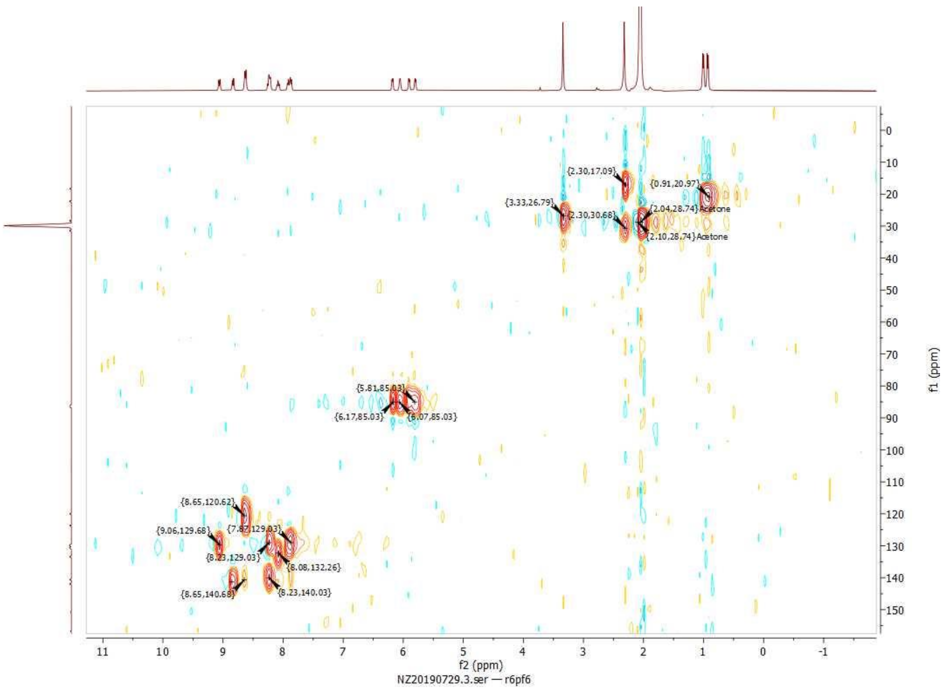

**Figure S29**. $^{1}$H-$^{13}$C-HSQC spectrum of **1** in $(CD_3)_2CO$

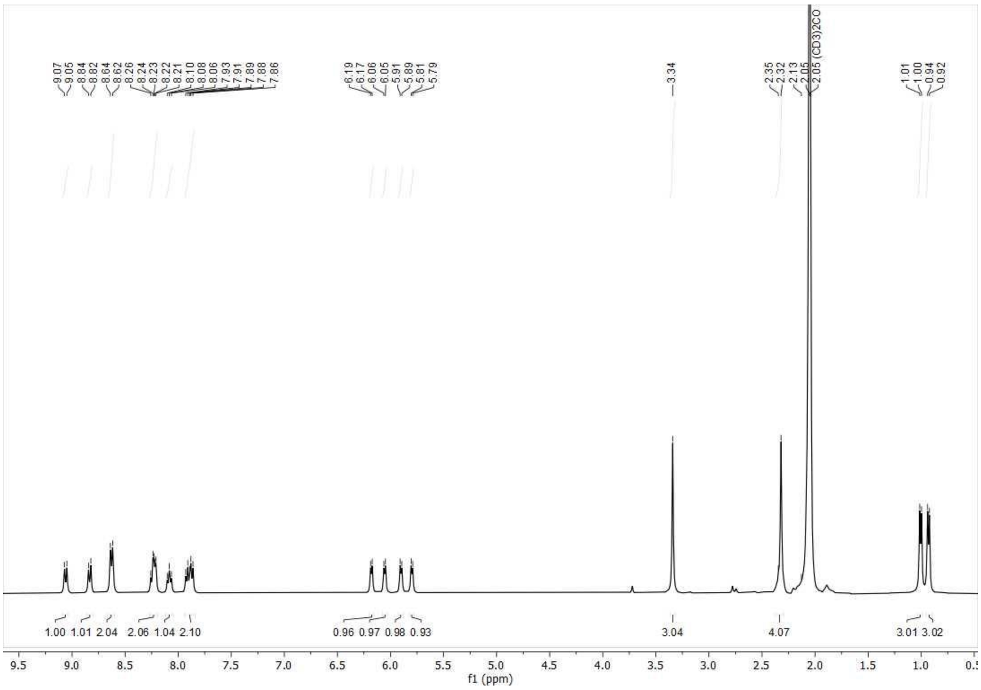

**Figure S30**. $^{1}$H-NMR spectrum of **2** in $(CD_3)_2CO$

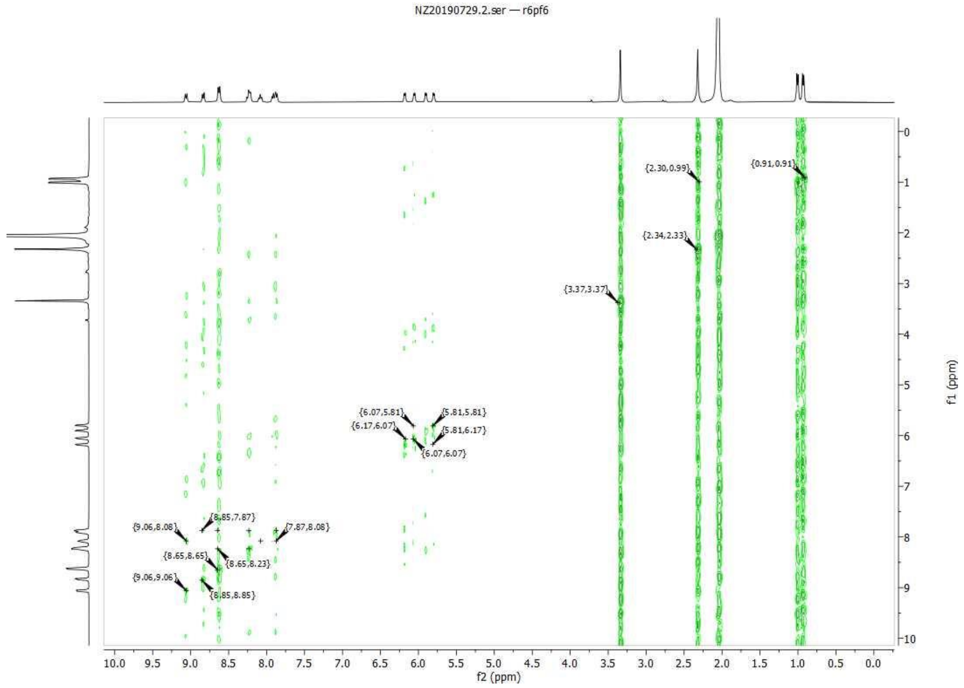


**Figure S31.** $^{1}H$-$^{1}H$ COSY spectrum of **2** in $(CD_3)_2CO$

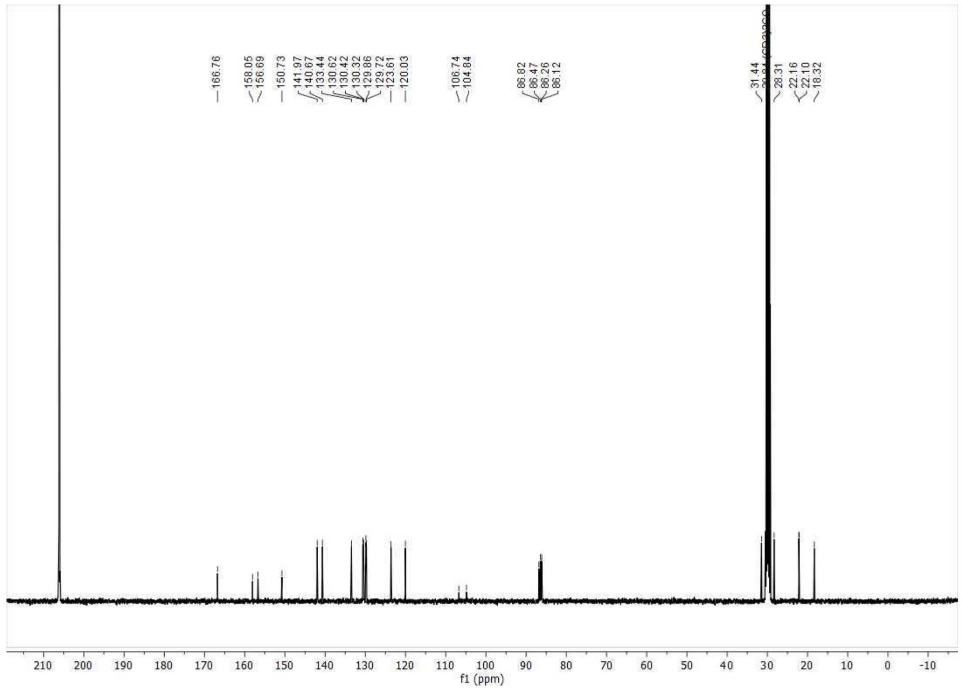


**Figure S32**. $^{13}C\{^{1}H\}$-NMR spectrum of **2** in $(CD_3)_2CO$

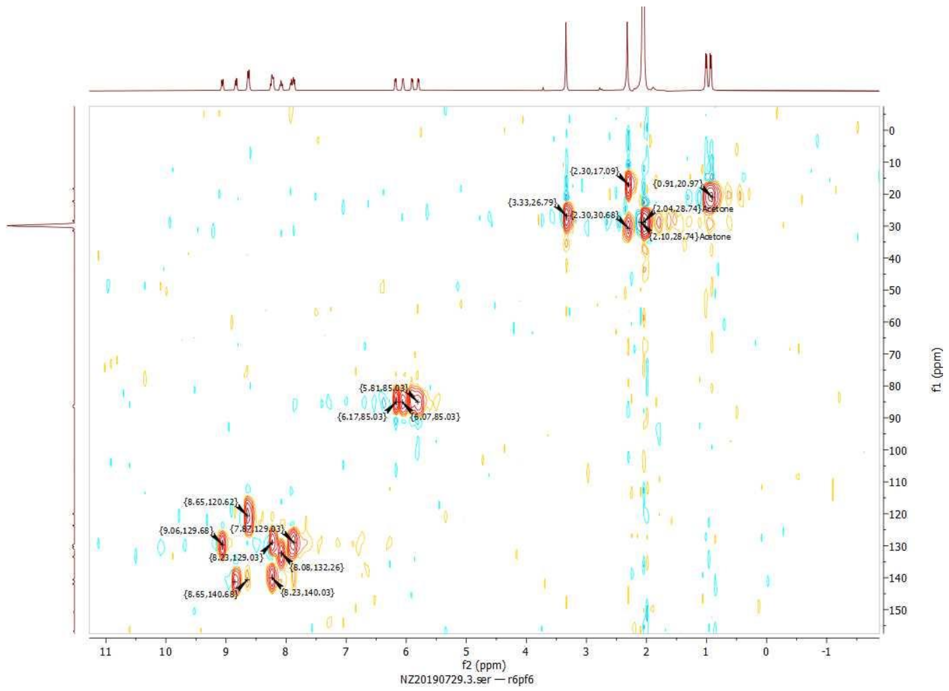


**Figure S33**. $^{1}H$-$^{13}C$-HSQC spectrum of **2** in $(CD_3)_2CO$

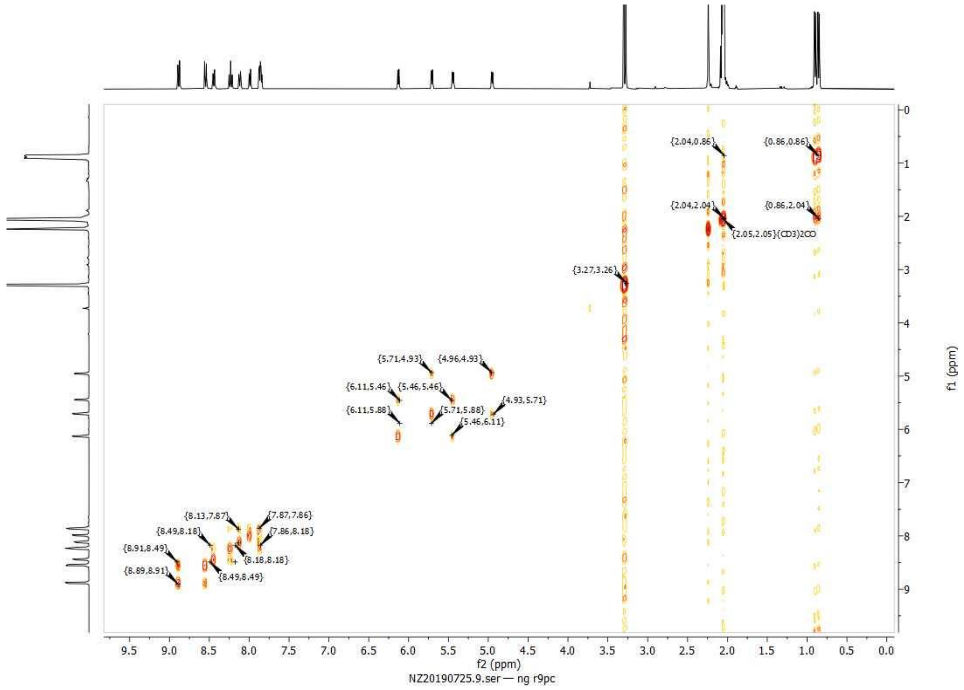


**Figure S34**. $^{1}H$-$^{1}H$ COSY spectrum of **3** in $(CD_3)_2CO$

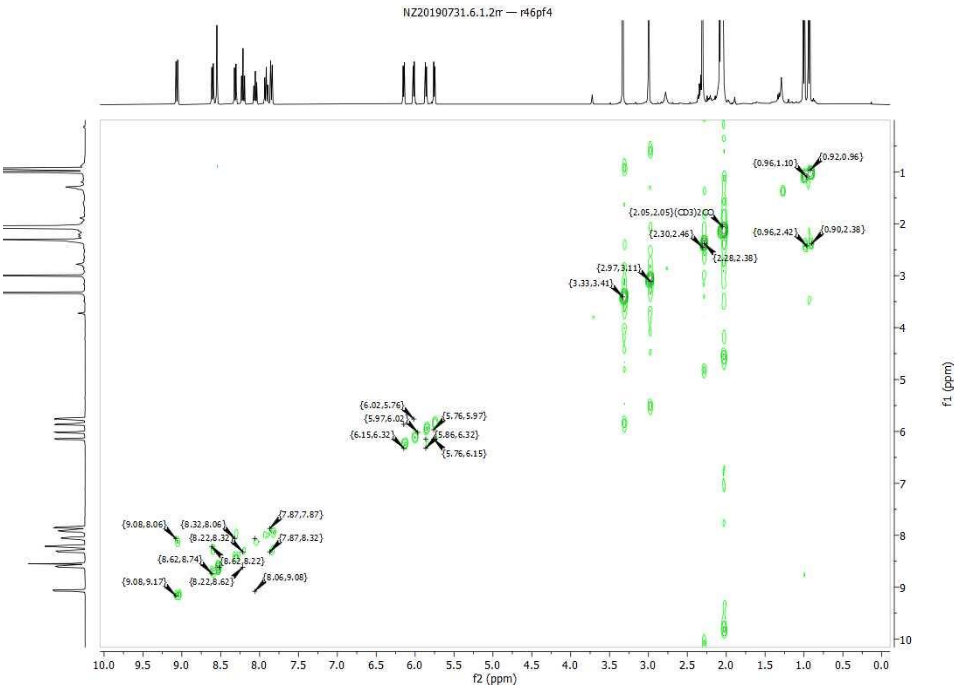

**Figure S35**. $^{1}H$-$^{1}H$ COSY spectrum of **4** in $(CD_3)_2CO$

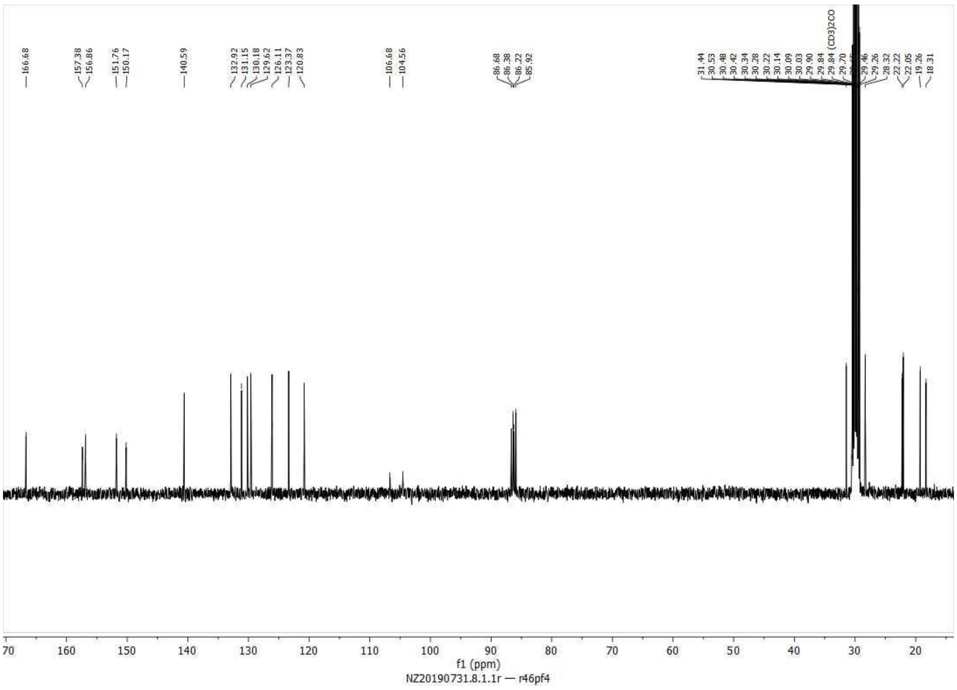

**Figure S36**. $^{13}C\{^{1}H\}$-NMR spectrum of **4** in $(CD_3)_2CO$

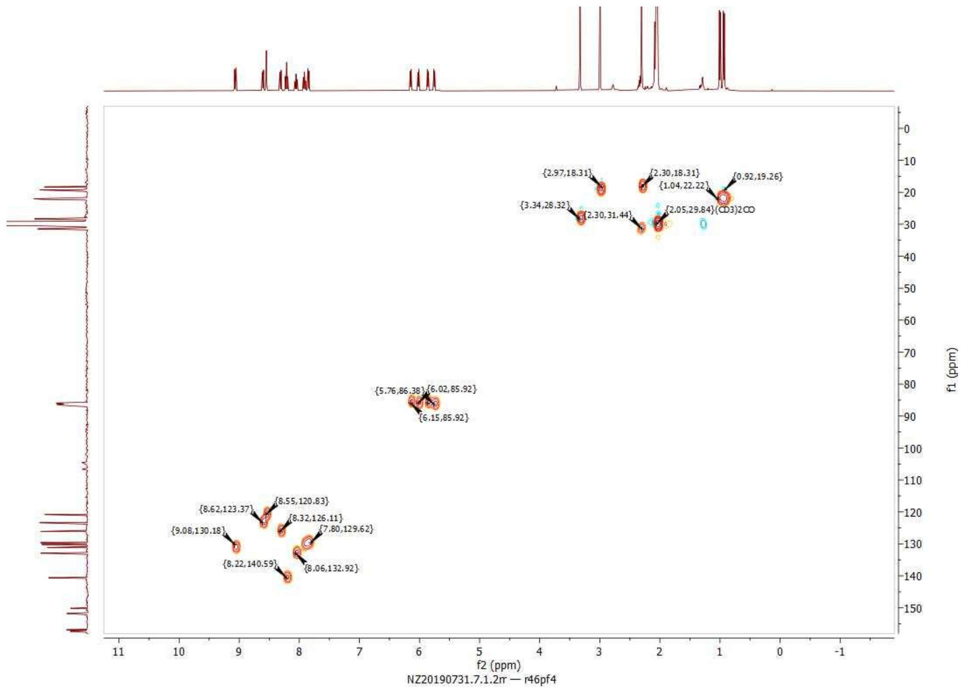

**Figure S37**. $^{1}$H-$^{13}$C-HSQC spectrum of **4** in $(CD_3)_2CO$

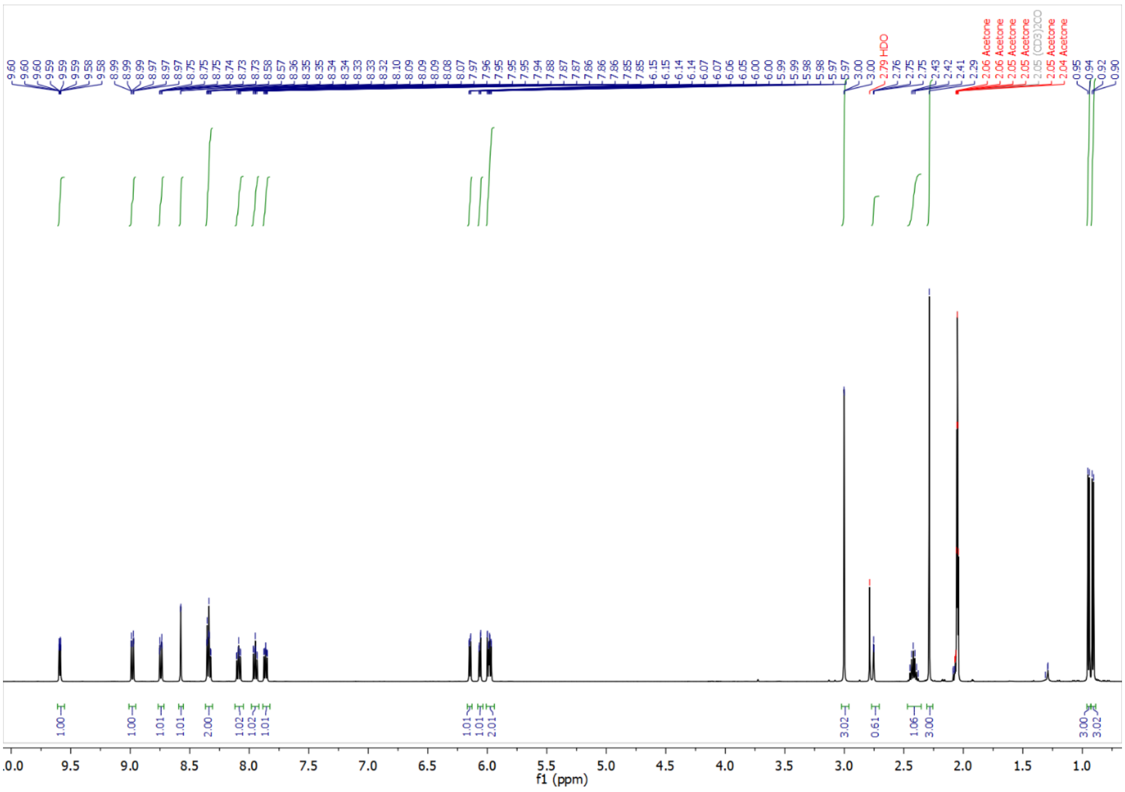

**Figure S38**. $^{1}$H-NMR spectrum of **5** in $(CD_3)_2CO$

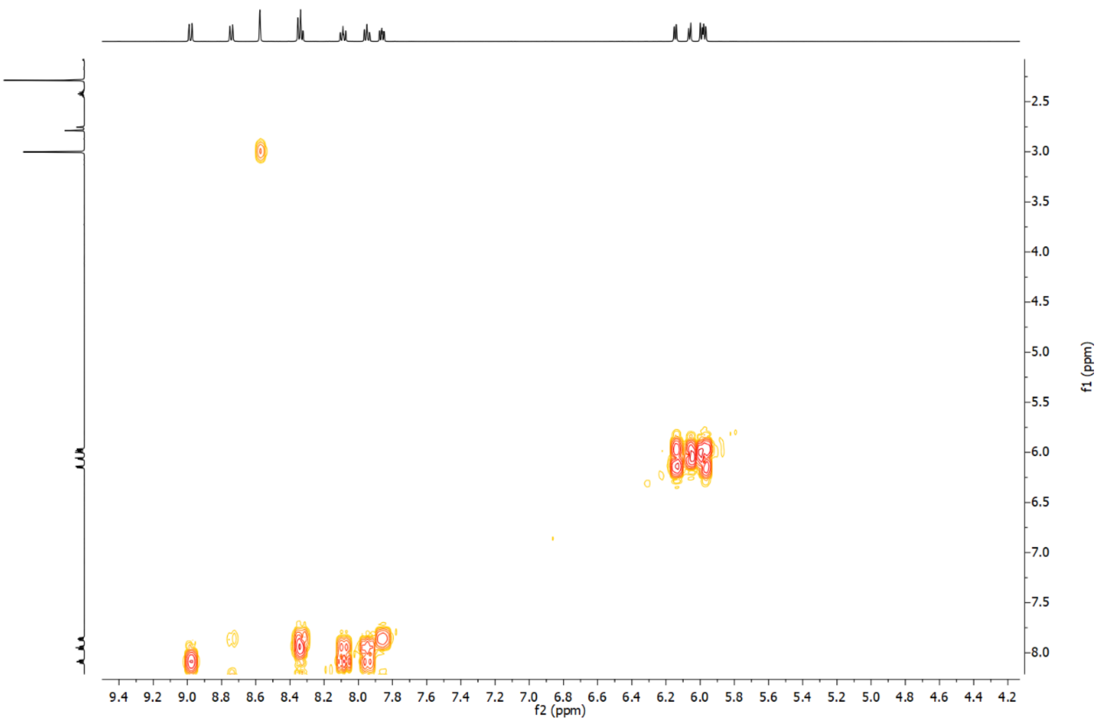

**Figure S39**.$^1$H-$^1$H COSY spectrum of **5** in $(CD_3)_2CO$

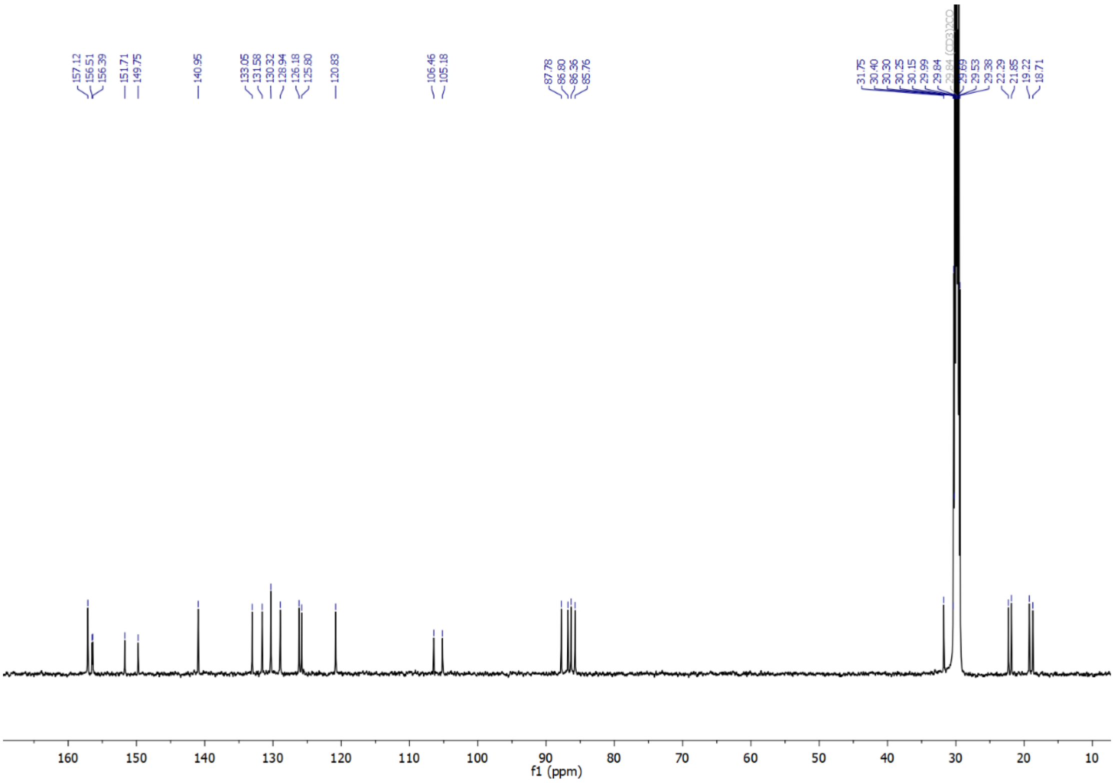

**Figure S40**. $^{13}C\{^1H\}$-NMR spectrum of **5** in $(CD_3)_2CO$

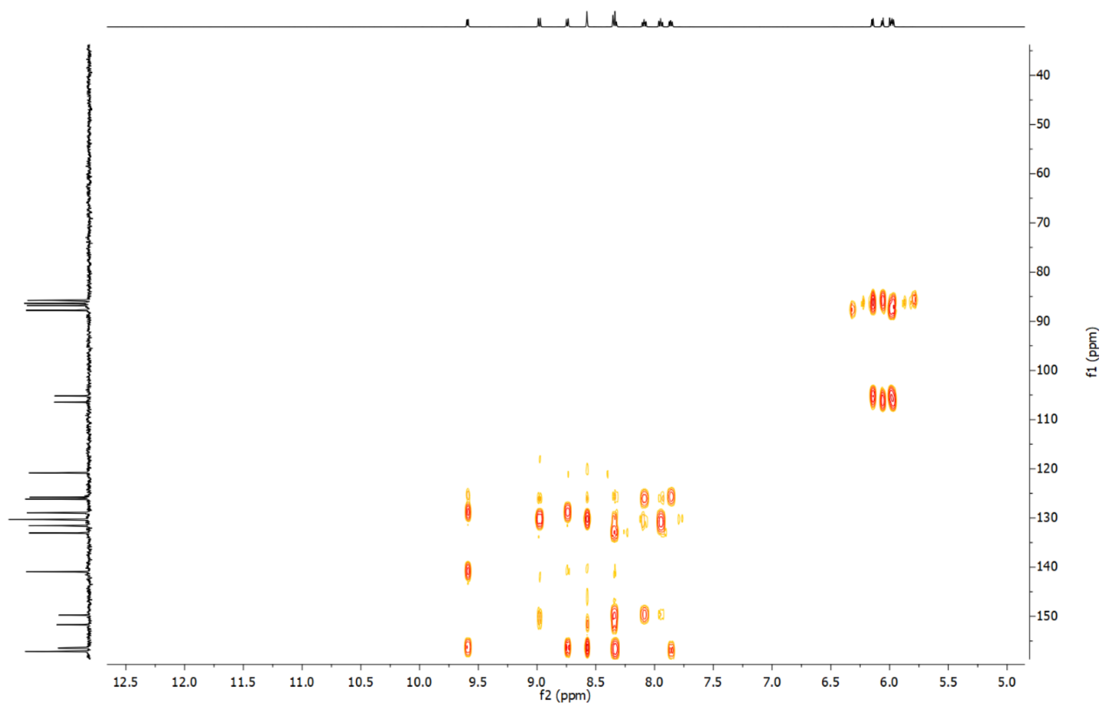

**Figure S41**. $^{1}$H-$^{13}$C-HSQC spectrum of **5** in $(CD_3)_2CO$

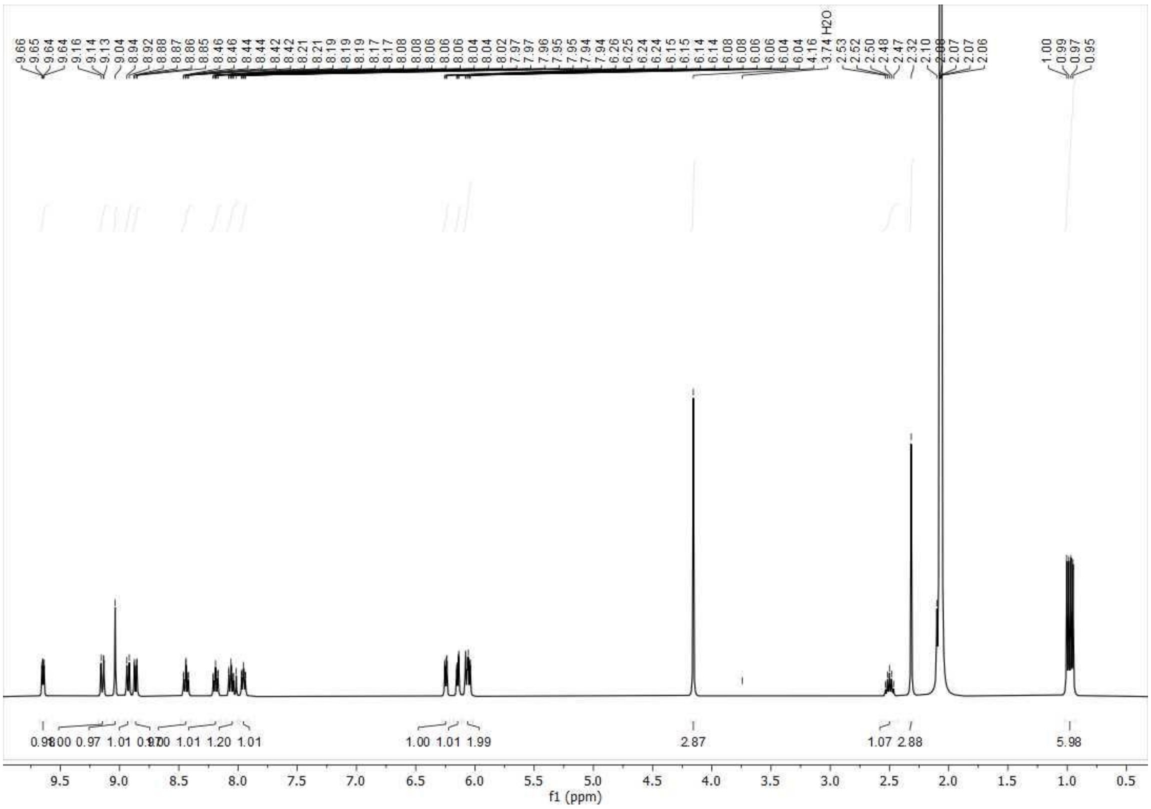

**Figure S42**. $^{1}$H-NMR spectrum of **Ru-pqcame** in $(CD_3)_2CO$

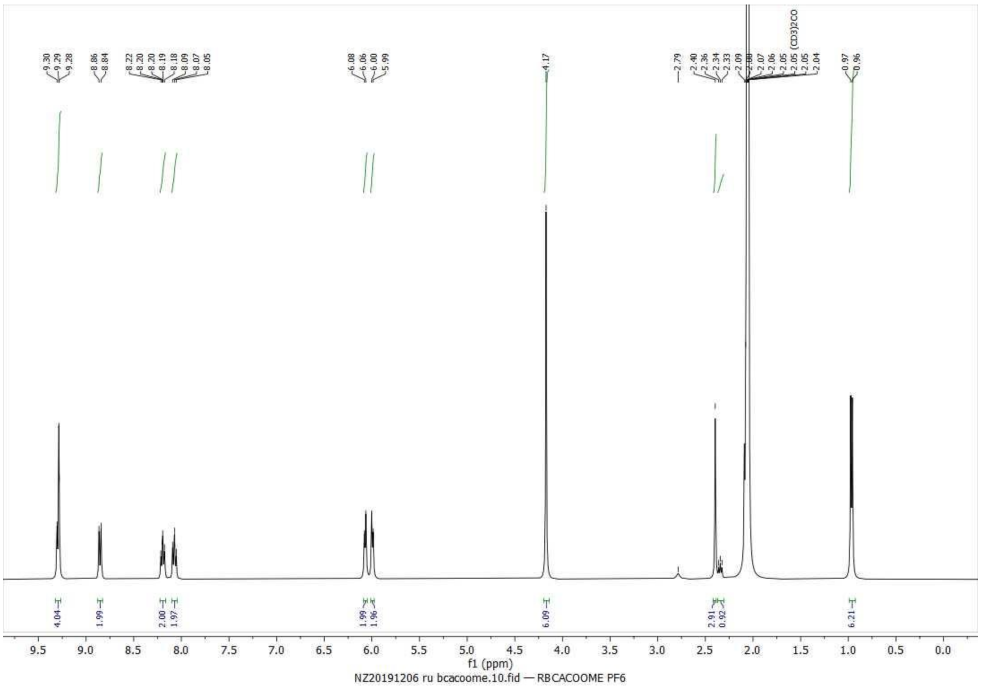

**Figure S43**. $^{1}$H-NMR spectrum of **6** in $(CD_3)_2CO$

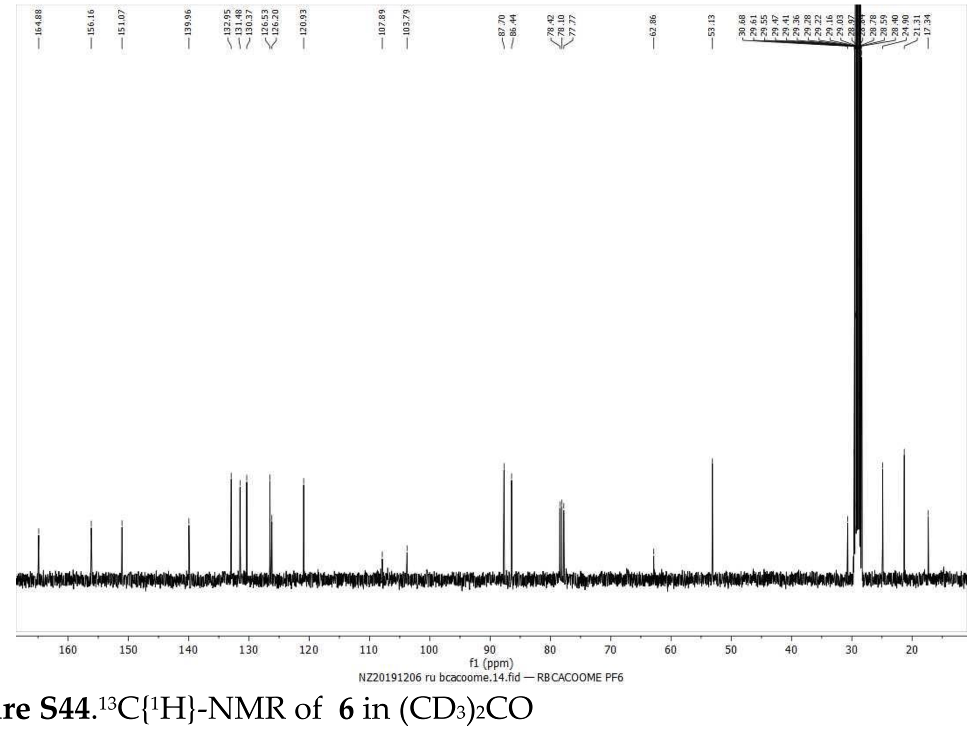

**Figure S44**. $^{13}C\{^{1}H\}$-NMR of **6** in $(CD_3)_2CO$

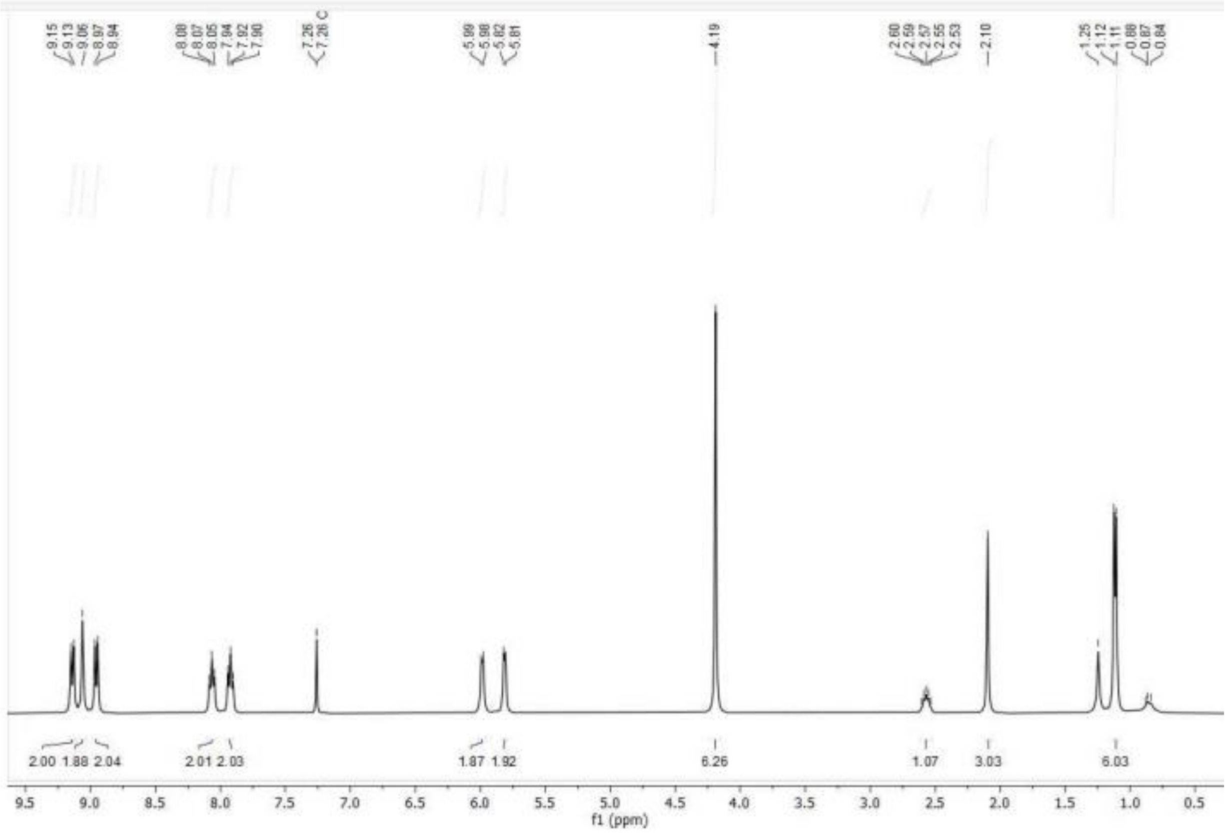


**Figure S45**. $^{1}H$-NMR spectrum of **6-Cl** in $CDCl_3$

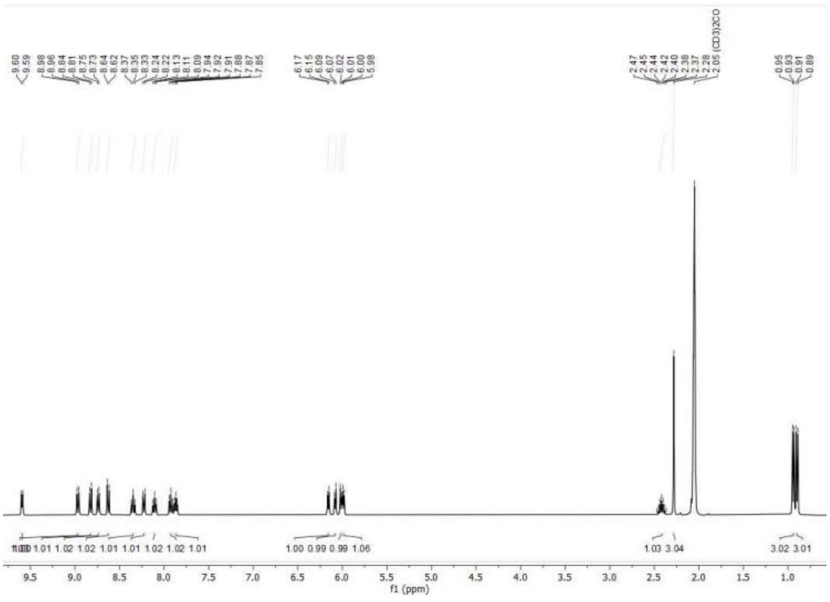

**Figure S46**. $^{1}$H-NMR spectrum of **7-Cl** in $(CD_3)_2CO$

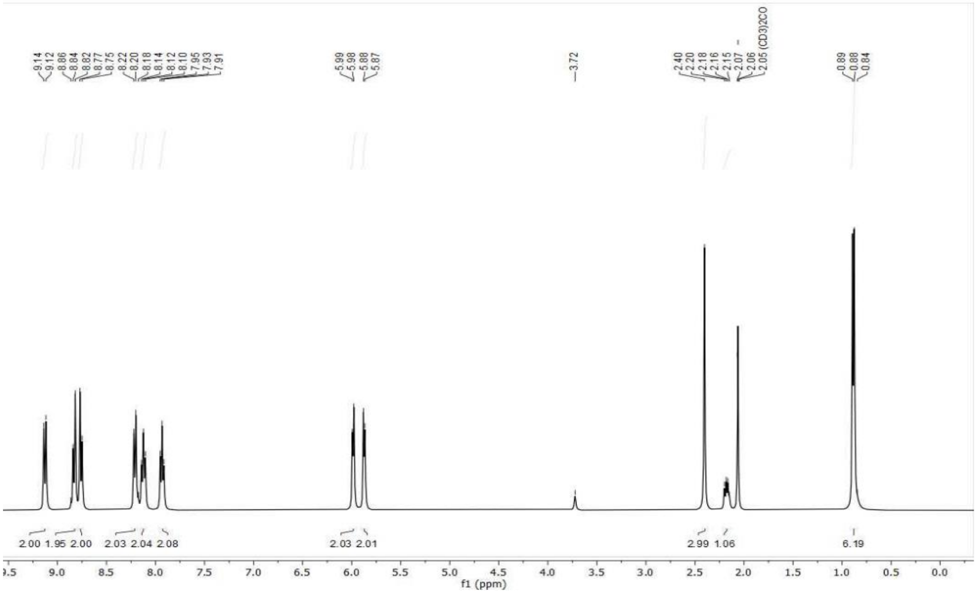

**Figure S47**. $^{1}$H-NMR spectrum of **8** in $(CD_3)_2CO$

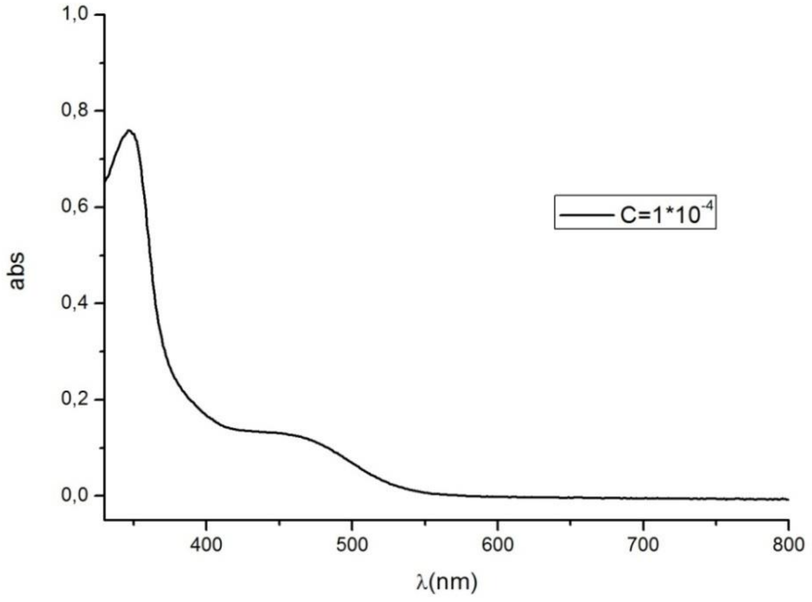


**Figure S48**. UV-vis spectrum of **1** in acetone

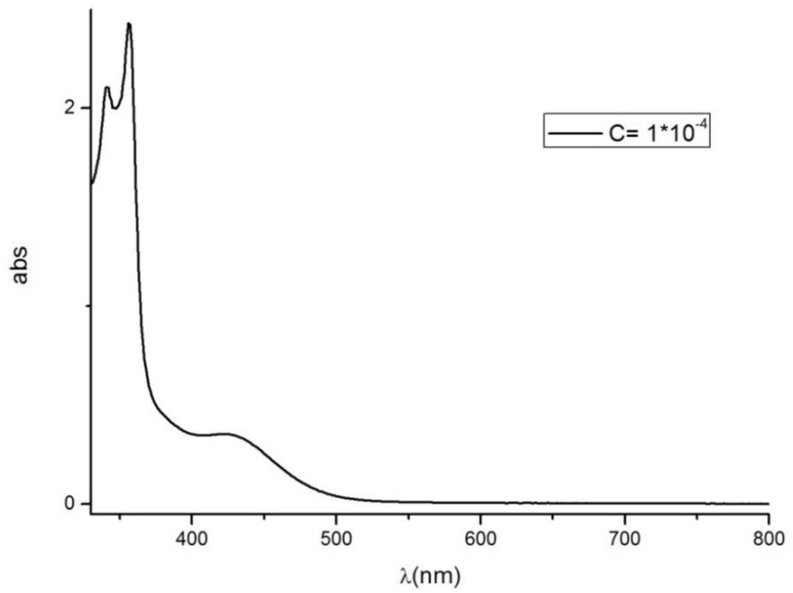


**Figure S49**. UV-vis spectrum of **2** in acetone

.

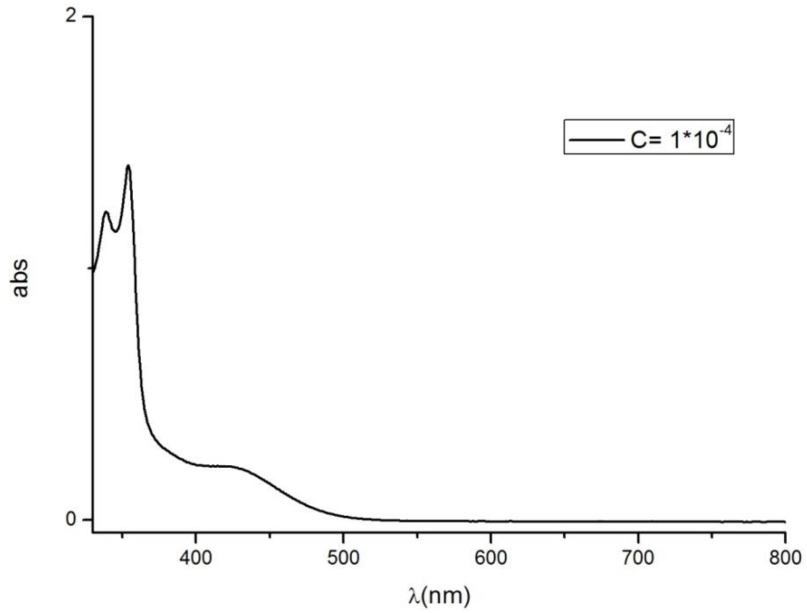


**Figure S50**. UV-vis spectrum of **3** in acetone

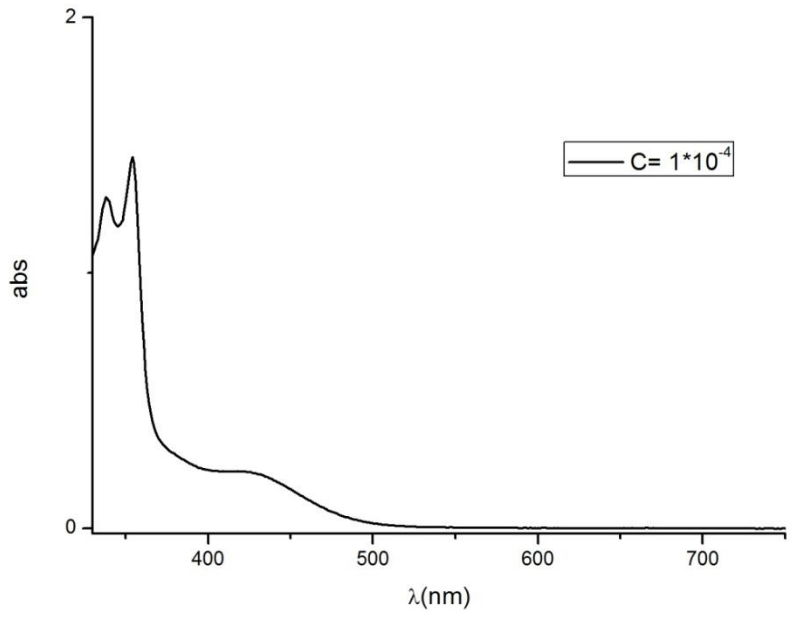


**Figure S51**. UV-vis spectrum of **4** in acetone

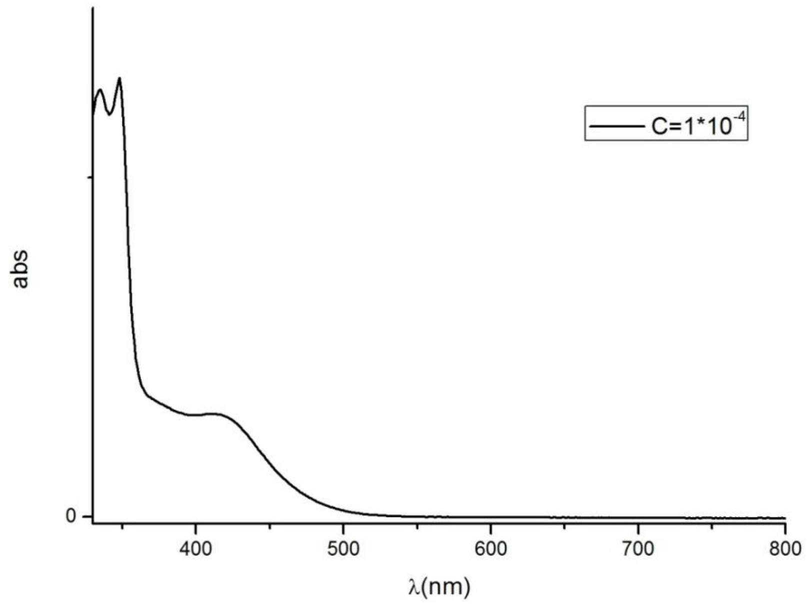


**Figure S52**. UV-vis spectrum of **5** in acetone

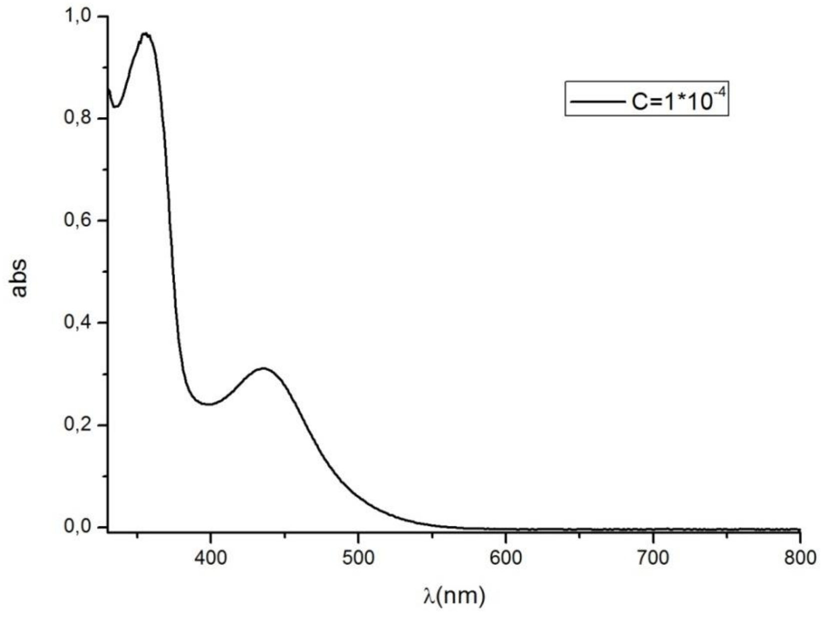


**Figure S53**. UV-vis spectrum of **Ru-pqcame** in acetone

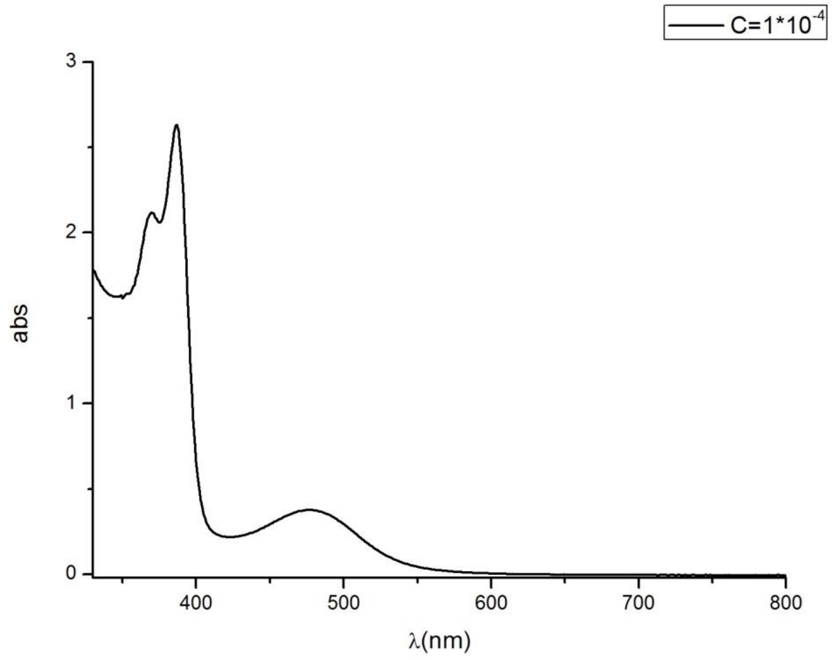


**Figure S54.** UV-vis spectrum of **6** in acetone

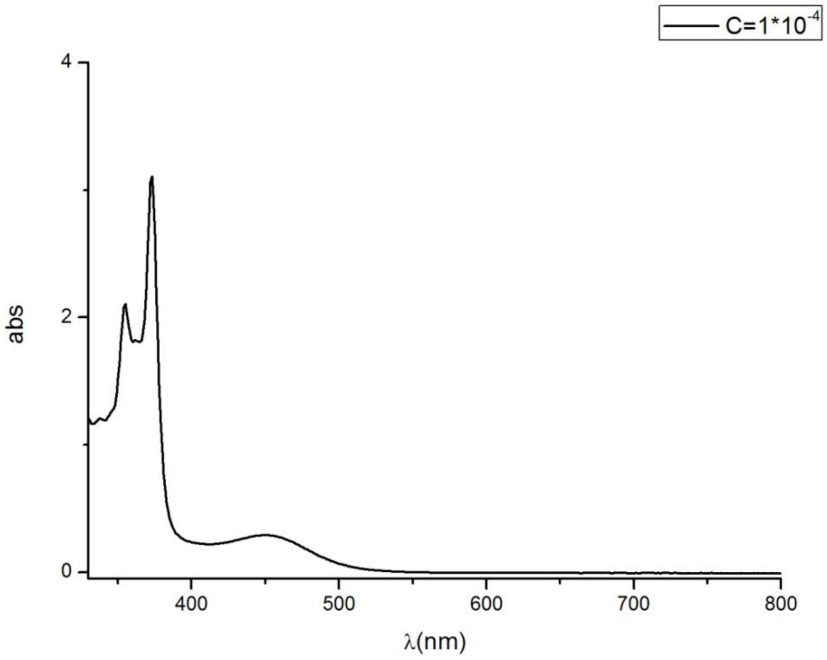


**Figure S55**. UV-vis spectrum of **8** in acetone

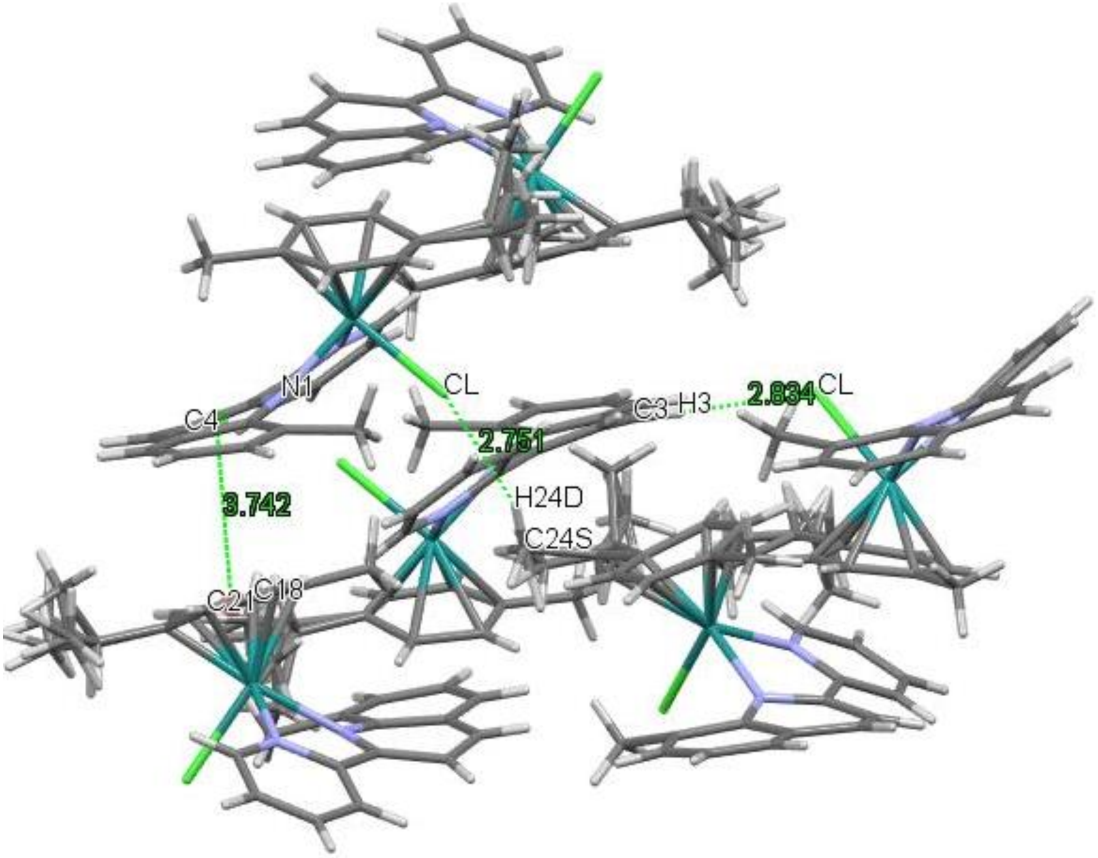


**Figure S56.** Intermolecular interactions in the unit cell of **1**

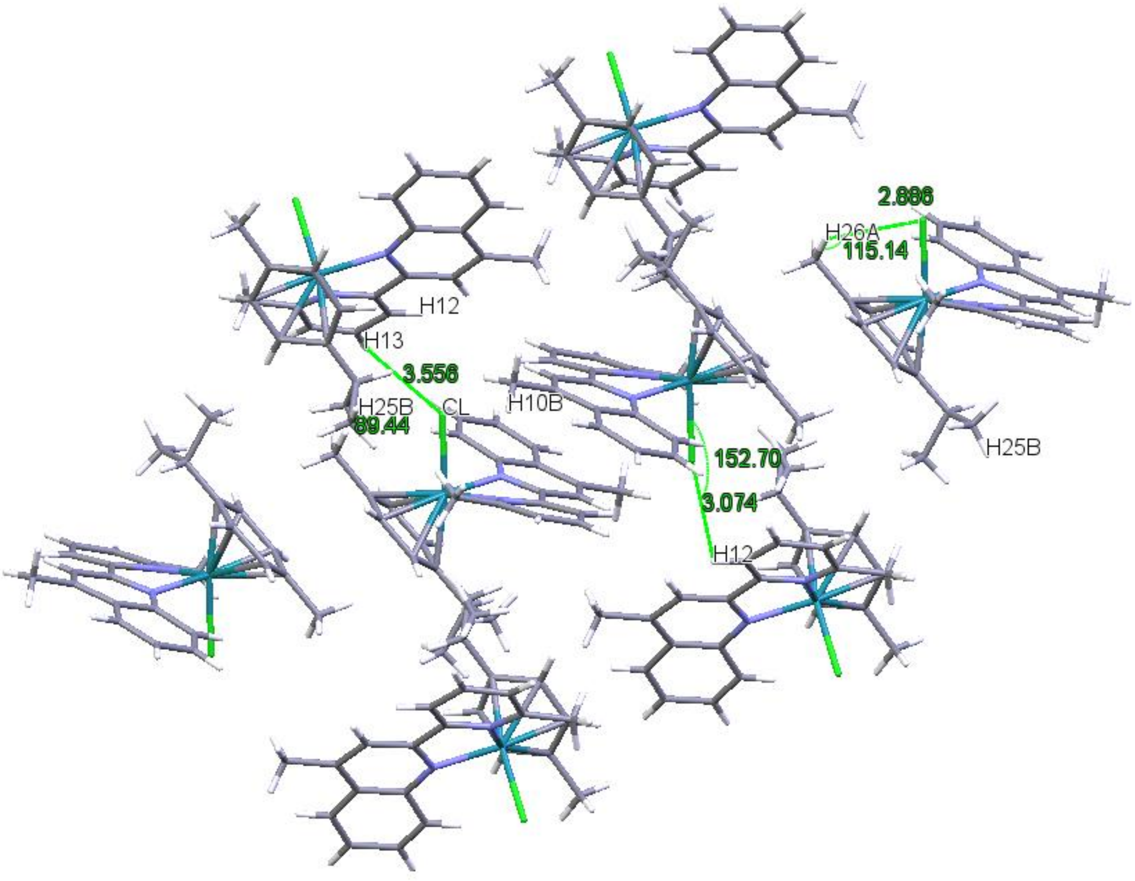


**Figure S57.** Intermolecular interactions in the unit cell of **4**

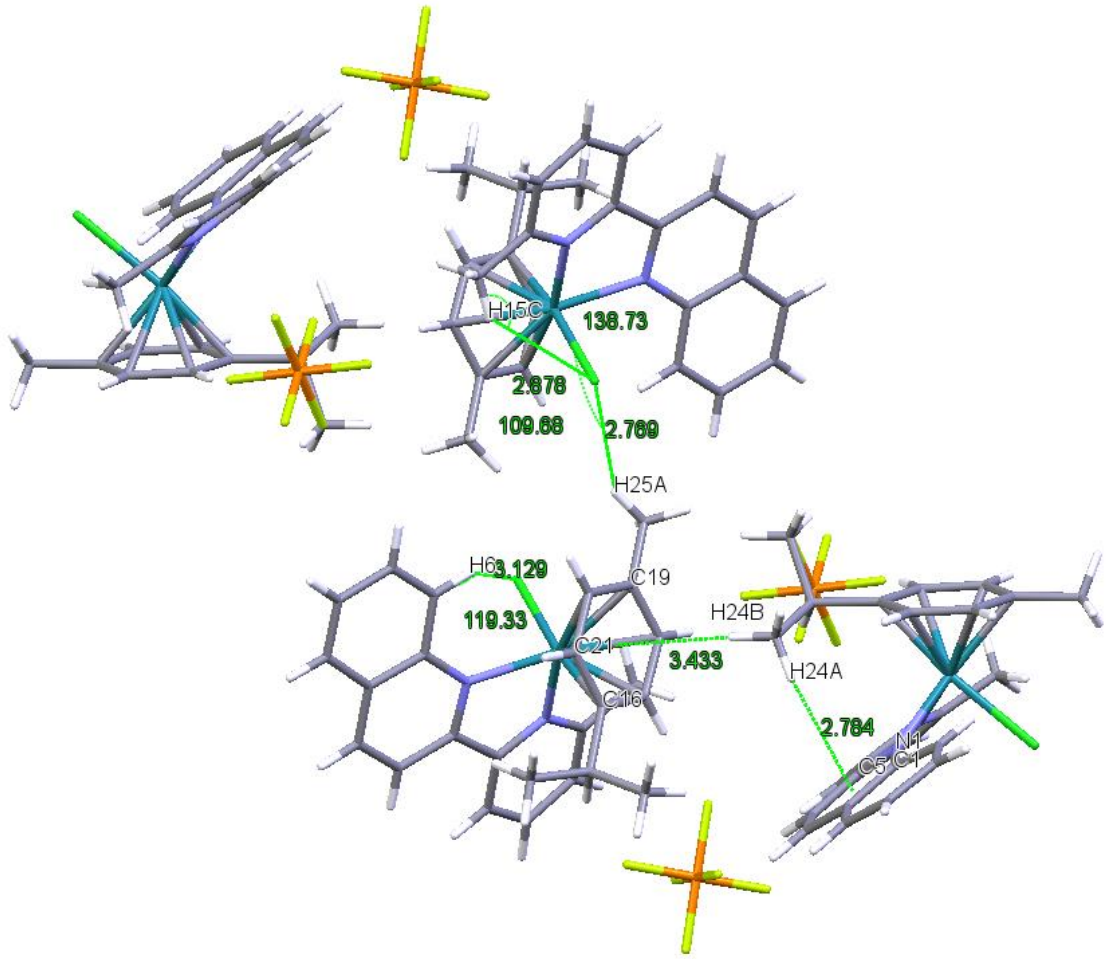


**Figure S58**. Intermolecular interactions in the unit cell of **2**

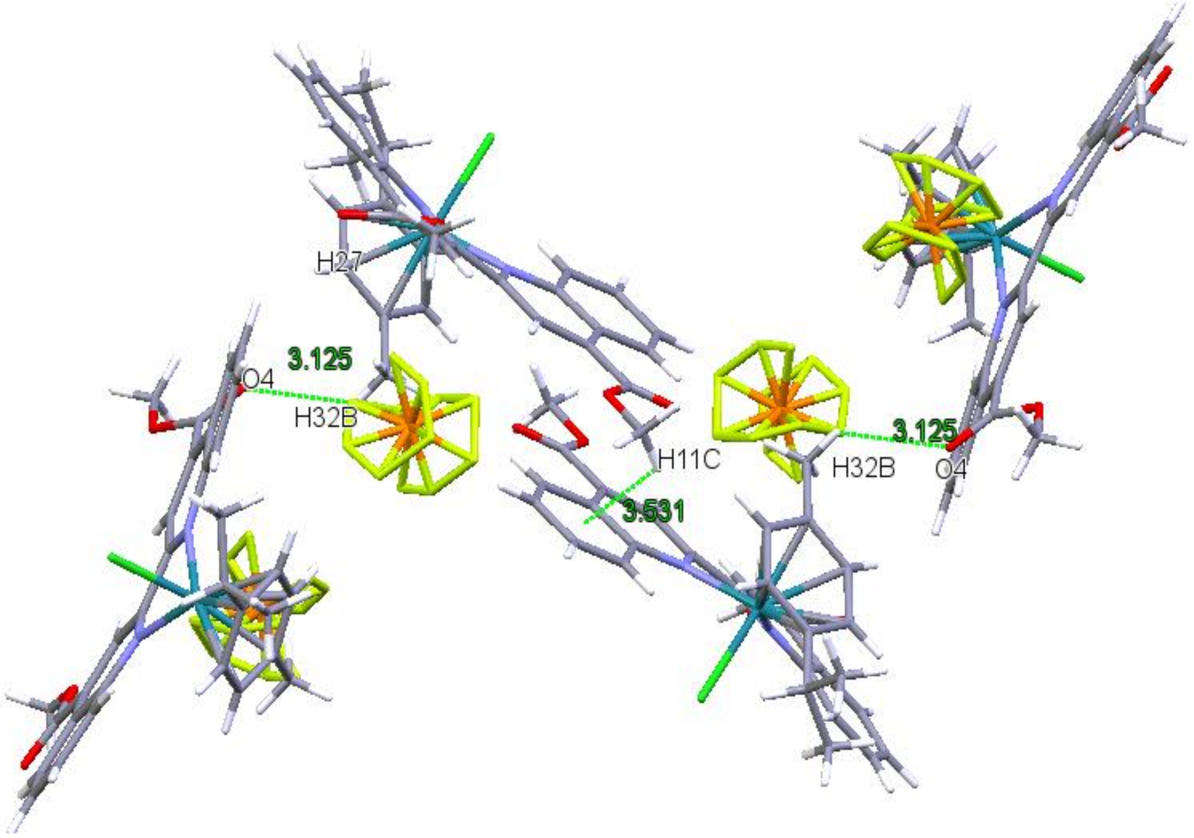


**Figure S59.** Intermolecular interactions in the unit cell of **6**

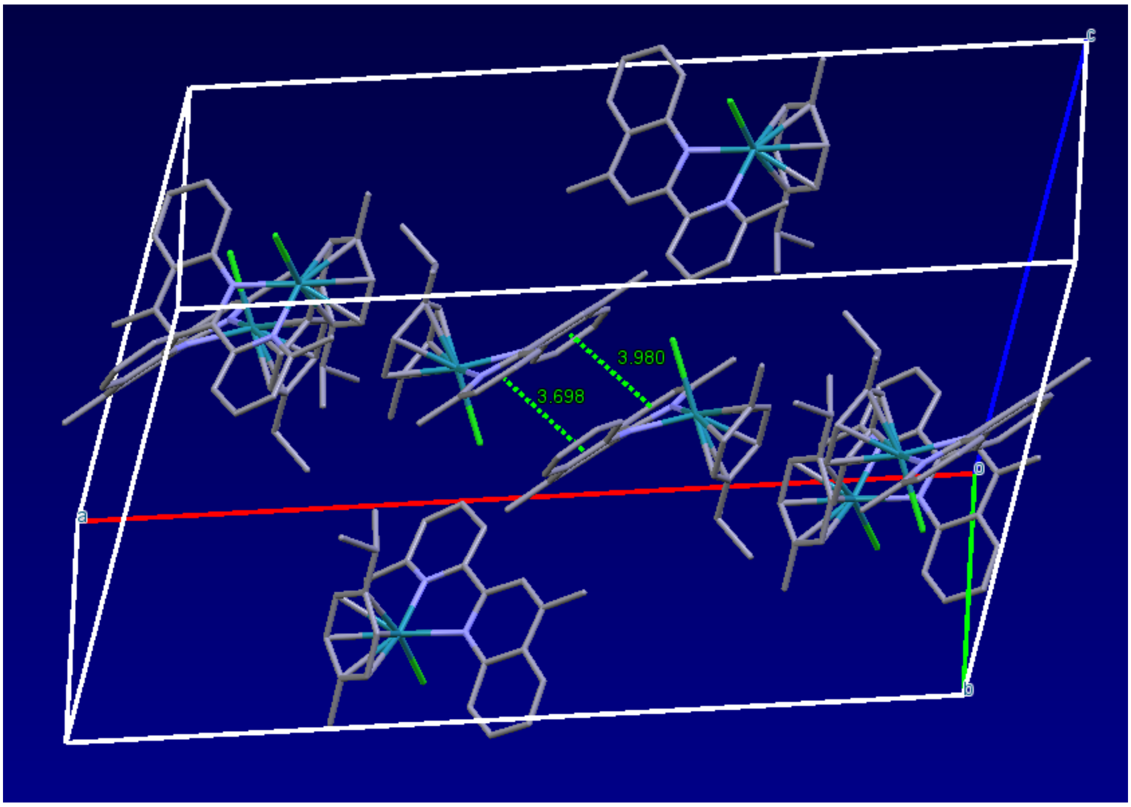


**Figure S60**. Intermolecular interactions in the unit cell of **3**

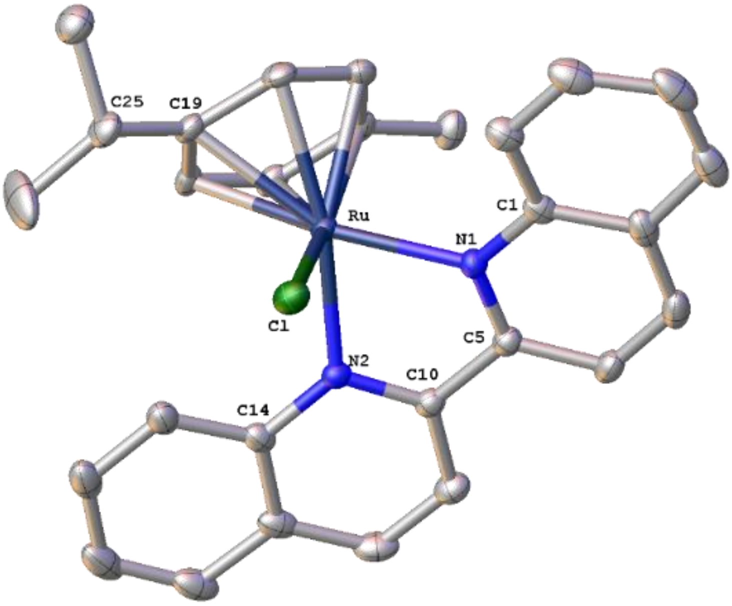


**Figure S61.** Molecular structure of cation of **8**. Hydrogen atoms and the $PF_6^-$ anion are omitted for clarity. The ellipsoids were plotted at the 50% probability.

**Table S1** Improved crystallographic data for **8**

Bond Lengths for **8**

| **Atom** | **Atom** | **Length/Å** | **Atom** | **Atom** | **Length/Å** |
|---|---|---|---|---|---|
| Ru | Cl | 2.3859(6) | C14 | C18 | 1.409(3) |
| Ru | N1 | 2.0931(18) | C15 | C16 | 1.354(4) |
| Ru | N2 | 2.1010(18) | C16 | C17 | 1.408(4) |
| Ru | C19 | 2.250(2) | C17 | C18 | 1.371(3) |
| Ru | C20 | 2.201(2) | C19 | C20 | 1.435(3) |
| Ru | C21 | 2.186(2) | C19 | C24 | 1.412(3) |
| Ru | C22 | 2.203(2) | C19 | C25 | 1.518(3) |
| Ru | C23 | 2.179(2) | C20 | C21 | 1.402(3) |
| Ru | C24 | 2.227(2) | C21 | C22 | 1.431(3) |
| N1 | C1 | 1.388(3) | C22 | C23 | 1.413(3) |
| N1 | C5 | 1.340(3) | C22 | C28 | 1.501(3) |
| N2 | C10 | 1.335(3) | C23 | C24 | 1.424(3) |
| N2 | C14 | 1.379(3) | C25 | C26 | 1.525(6) |

Bond Angles for **8**

| **Atom** | **Atom** | **Atom** | **Angle/°** | **Atom** | **Atom** | **Atom** | **Angle/°** |
|---|---|---|---|---|---|---|---|
| N1 | Ru | Cl | 86.52(5) | N2 | C14 | C18 | 119.8(2) |
| N1 | Ru | N2 | 76.49(7) | C18 | C14 | C13 | 119.8(2) |
| N1 | Ru | C19 | 153.20(8) | C16 | C15 | C13 | 120.3(3) |
| N1 | Ru | C20 | 116.03(8) | C15 | C16 | C17 | 120.8(2) |
| N1 | Ru | C21 | 91.84(8) | C18 | C17 | C16 | 120.5(3) |
| N1 | Ru | C22 | 93.16(8) | C17 | C18 | C14 | 119.8(2) |
| N1 | Ru | C23 | 121.35(8) | C20 | C19 | Ru | 69.34(12) |
| N1 | Ru | C24 | 159.04(8) | C20 | C19 | C25 | 118.8(2) |
| N2 | Ru | Cl | 87.59(5) | C24 | C19 | Ru | 70.75(13) |
| N2 | Ru | C19 | 130.22(8) | C24 | C19 | C20 | 117.7(2) |
| N2 | Ru | C20 | 166.78(8) | C24 | C19 | C25 | 123.5(2) |
| N2 | Ru | C21 | 144.40(8) | C25 | C19 | Ru | 129.35(16) |
| N2 | Ru | C22 | 108.14(8) | C19 | C20 | Ru | 73.05(13) |
| N2 | Ru | C23 | 89.89(8) | C21 | C20 | Ru | 70.81(13) |
| N2 | Ru | C24 | 100.04(8) | C21 | C20 | C19 | 120.7(2) |
| C19 | Ru | Cl | 91.91(6) | C20 | C21 | Ru | 71.92(13) |
| C20 | Ru | Cl | 97.05(6) | C20 | C21 | C22 | 122.0(2) |
| C20 | Ru | C19 | 37.61(8) | C22 | C21 | Ru | 71.58(13) |
| C20 | Ru | C22 | 68.51(9) | C21 | C22 | Ru | 70.35(13) |
| C20 | Ru | C24 | 66.77(9) | C21 | C22 | C28 | 121.4(2) |
| C21 | Ru | Cl | 125.69(7) | C23 | C22 | Ru | 70.27(13) |
| C21 | Ru | C19 | 67.52(8) | C23 | C22 | C21 | 116.8(2) |
| C21 | Ru | C20 | 37.27(9) | C23 | C22 | C28 | 121.6(2) |
| C21 | Ru | C22 | 38.07(9) | C28 | C22 | Ru | 126.85(16) |
| C21 | Ru | C24 | 78.99(9) | C22 | C23 | Ru | 72.10(13) |

| | | | | | | | |
|---|---|---|---|---|---|---|---|
| C22 | Ru | Cl | 163.76(7) | C22 | C23 | C24 | 121.6(2) |
| C22 | Ru | C19 | 81.08(8) | C24 | C23 | Ru | 72.99(13) |
| C22 | Ru | C24 | 68.00(8) | C19 | C24 | Ru | 72.49(13) |
| C23 | Ru | Cl | 150.52(6) | C19 | C24 | C23 | 121.1(2) |
| C23 | Ru | C19 | 67.74(8) | C23 | C24 | Ru | 69.31(12) |
| C23 | Ru | C20 | 79.97(9) | C19 | C25 | C26 | 111.3(3) |
| C23 | Ru | C21 | 67.45(9) | C19 | C25 | C26S | 106.6(3) |
| C23 | Ru | C22 | 37.63(9) | C27 | C25 | C19 | 115.5(2) |
| C23 | Ru | C24 | 37.70(8) | C27 | C25 | C26 | 116.3(4) |
| C24 | Ru | Cl | 114.17(6) | C27 | C25 | C26S | 99.7(5) |

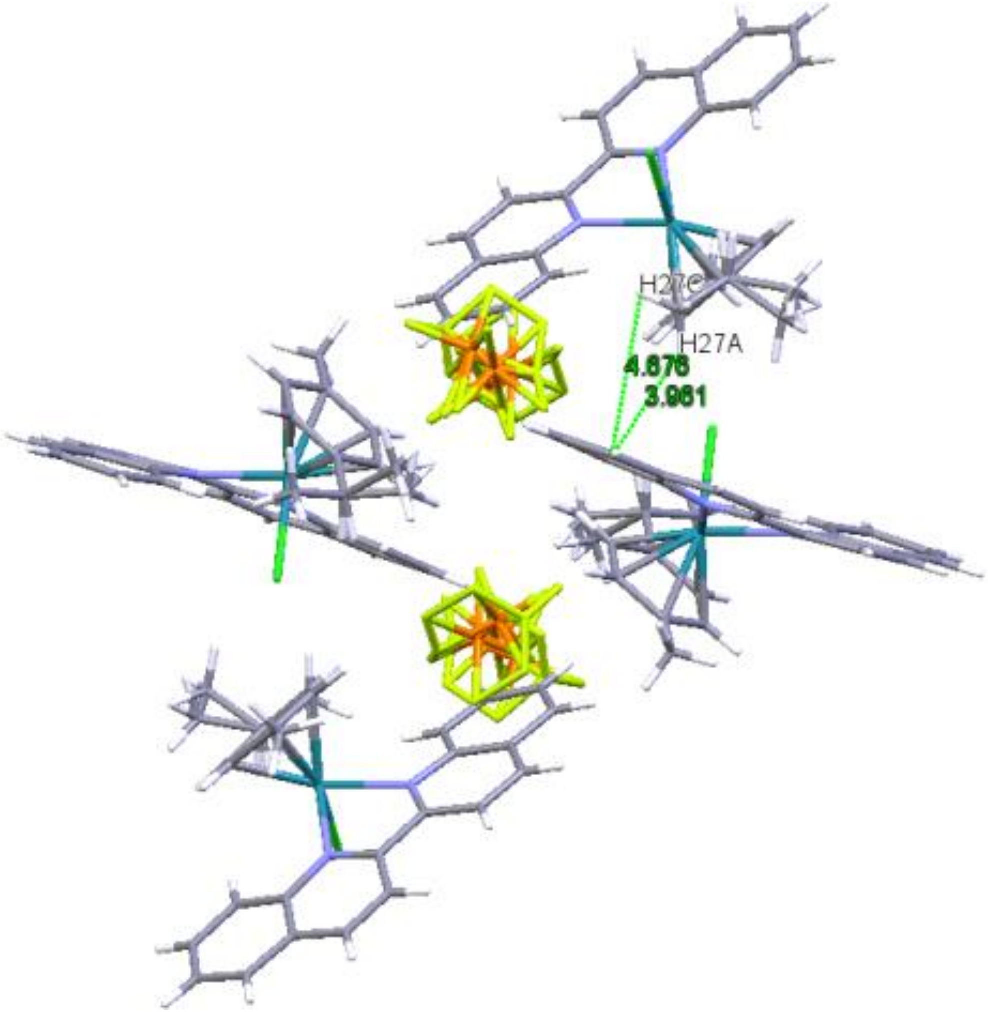


**Figure S62**. Intermolecular interactions in the unit cell of **8**

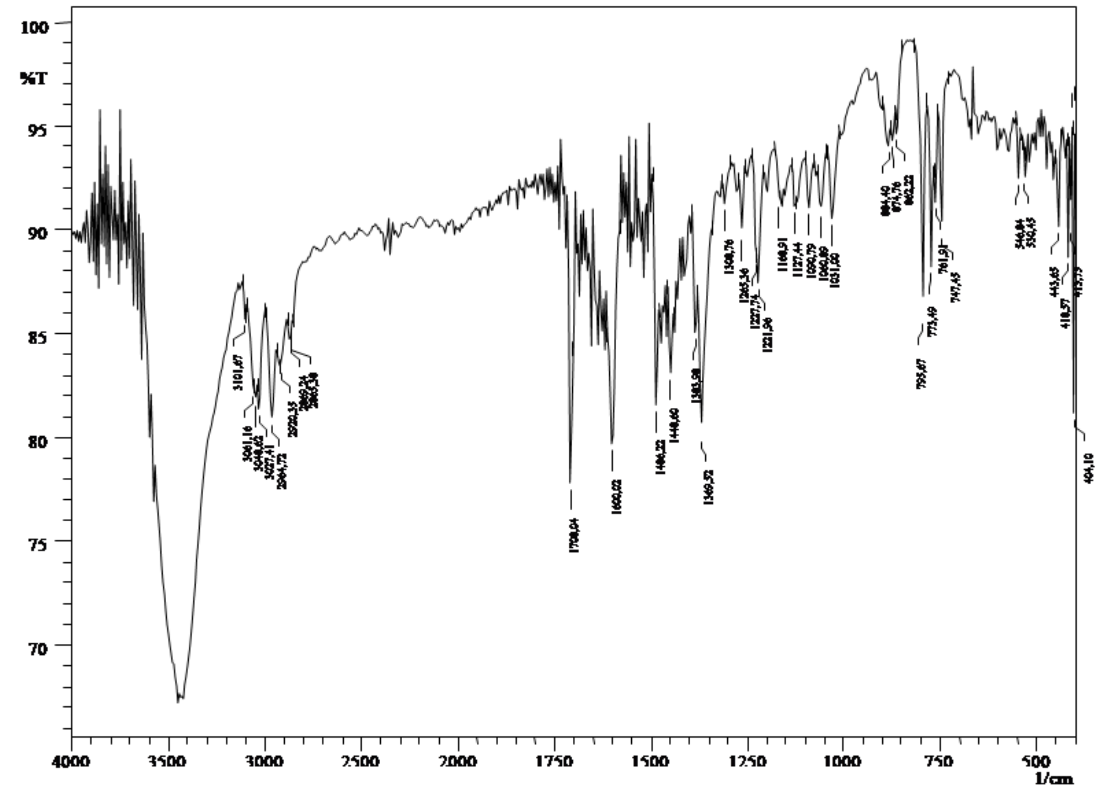


**Figure S63.** FT-IR spectrum of **9**

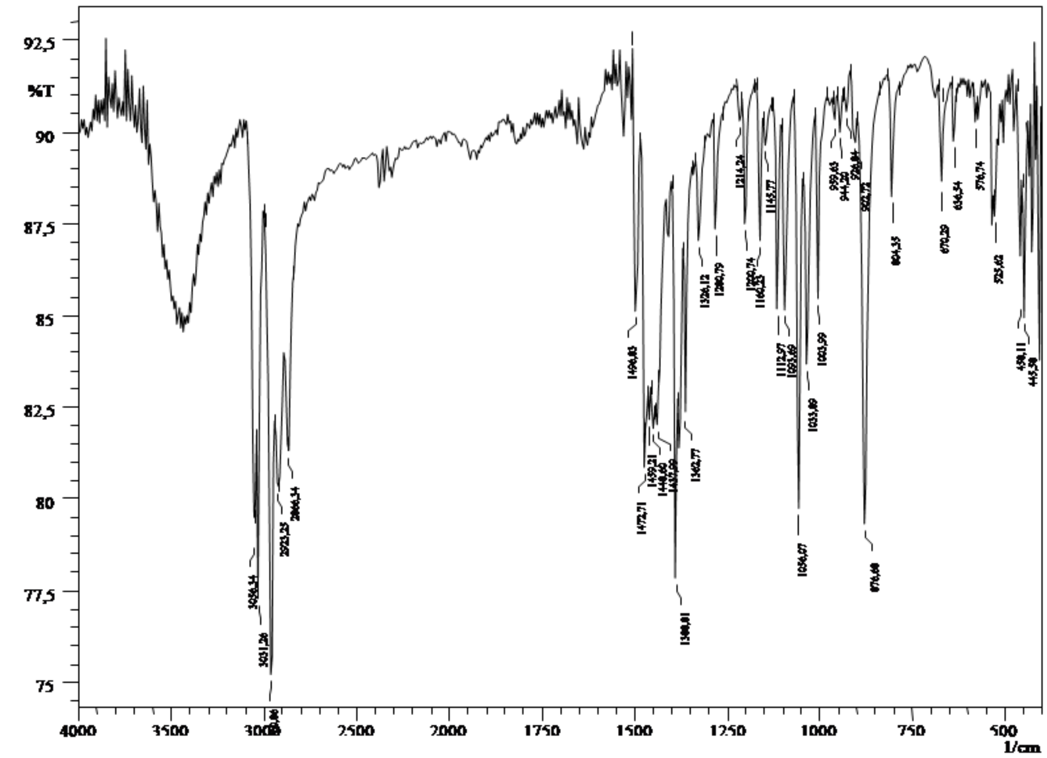


**Figure S64.** FT-IR spectrum of **10**

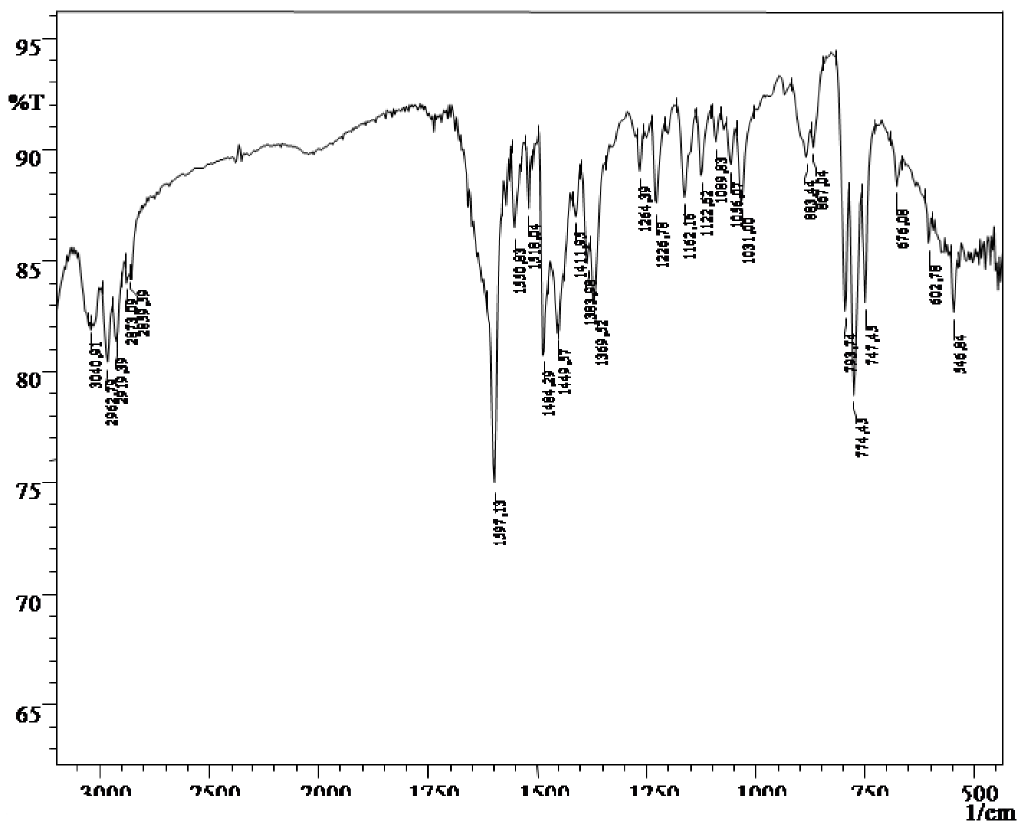


**Figure S65.** FT-IR spectrum of **11**

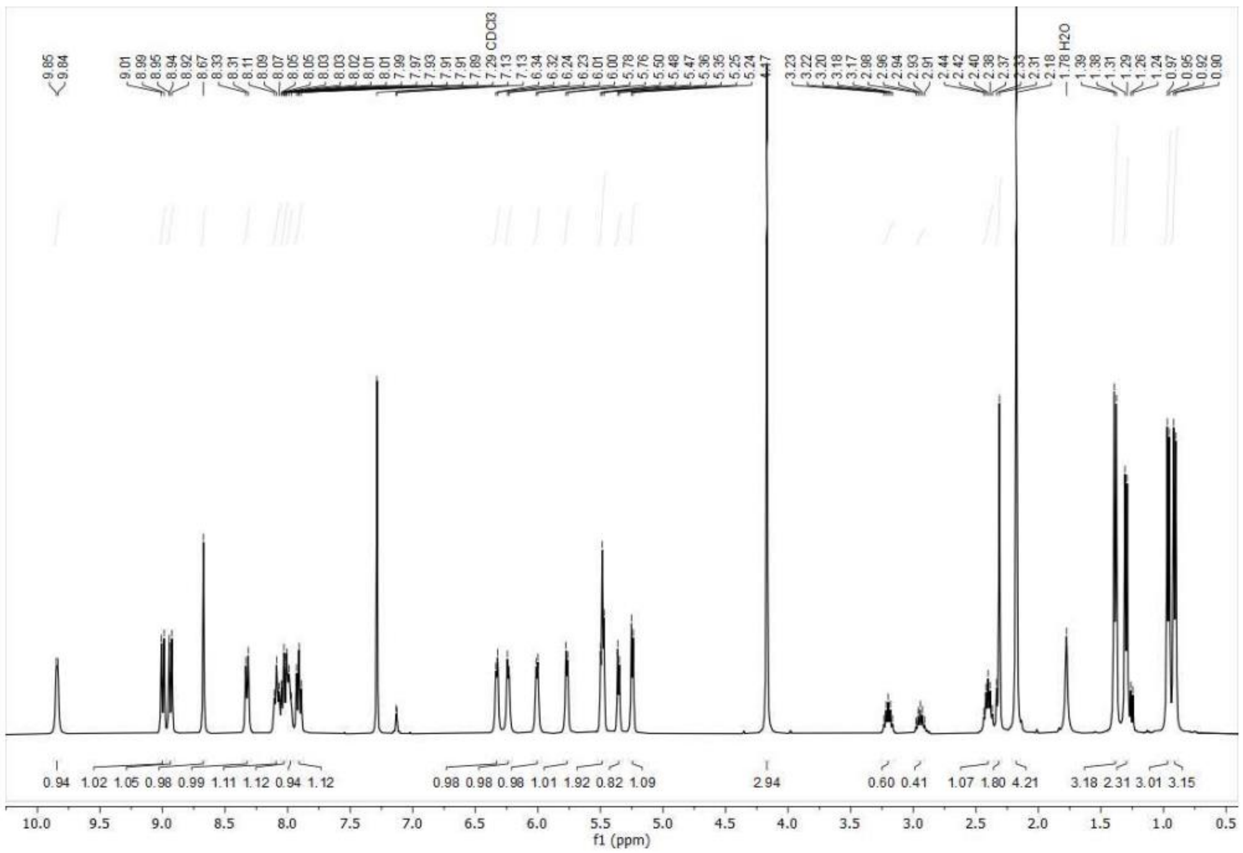

**Figure S66.** $^{1}H$-NMR spectrum of **9** in $CDCl_3$

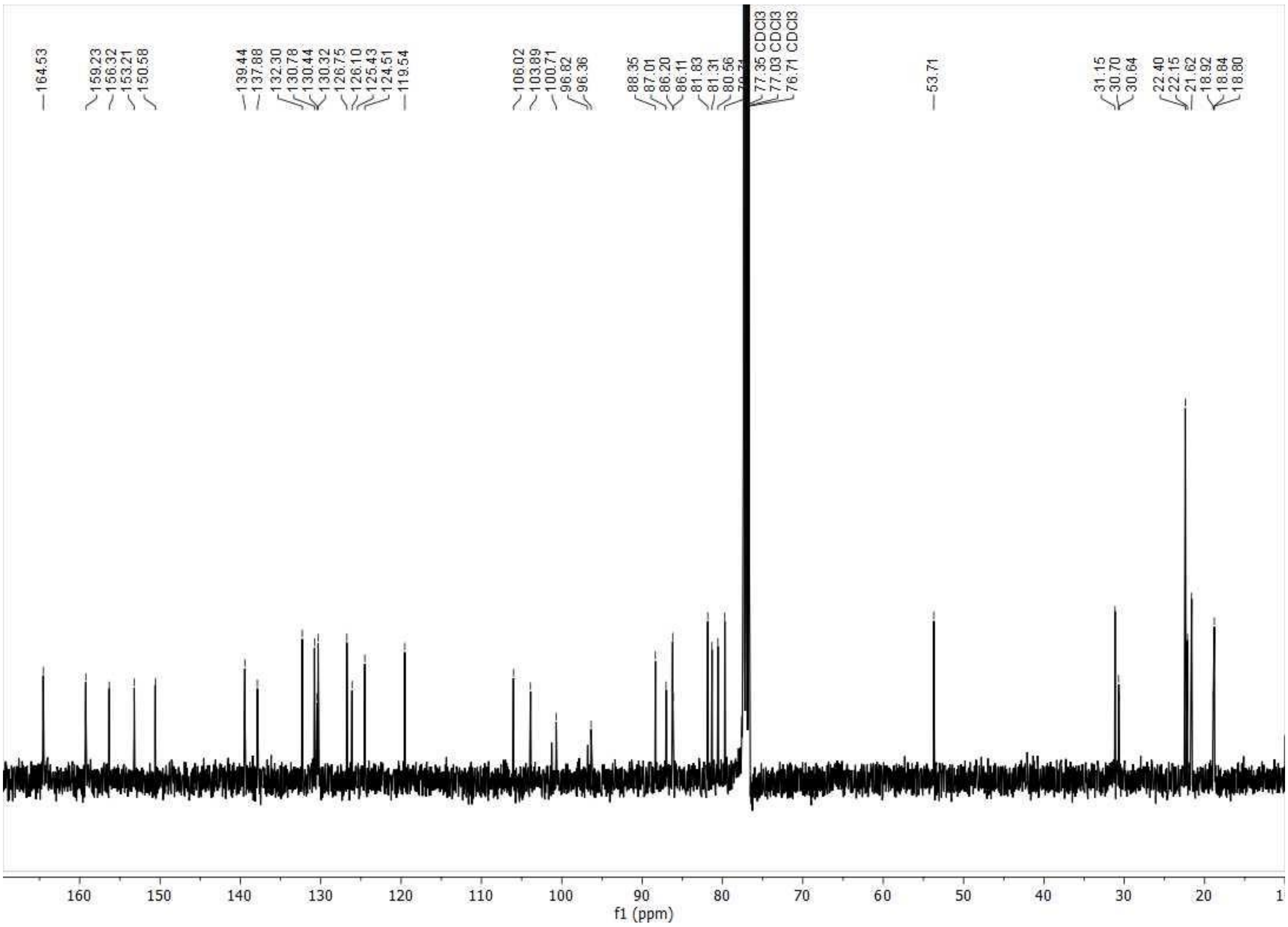

**Figure S67**. $^{13}C\{^{1}H\}$-NMR spectrum of **9** in $CDCl_3$

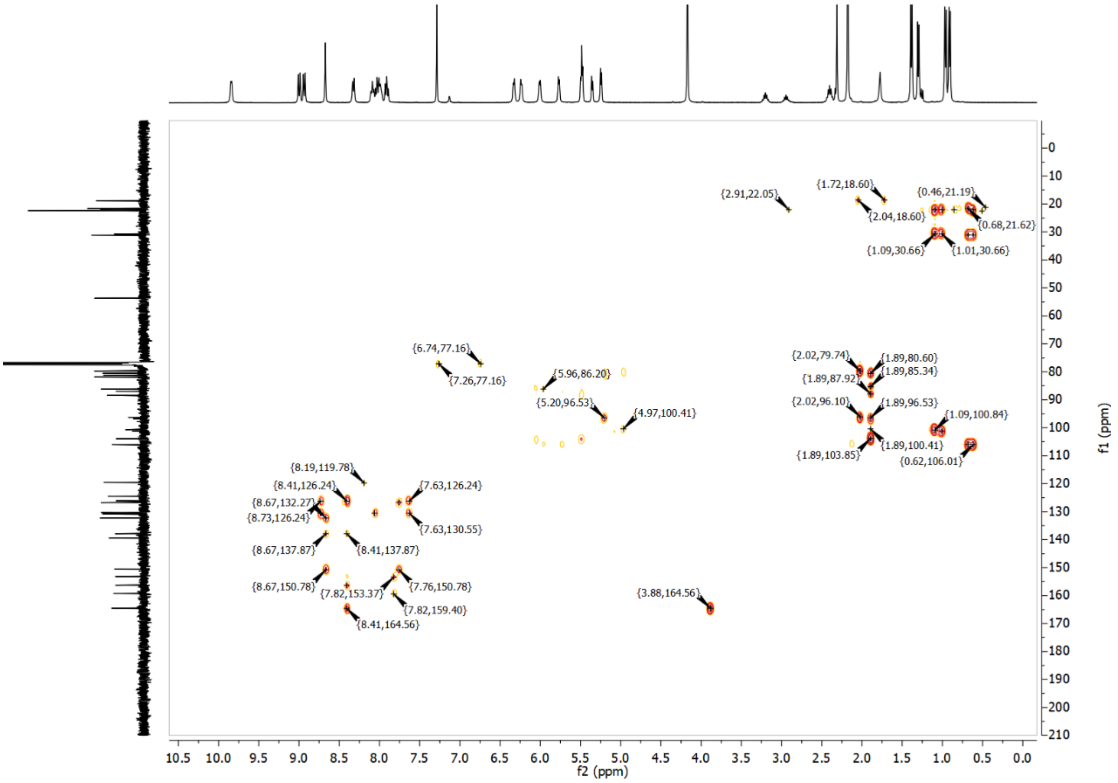


**Figure S68**. $^{1}$H-$^{13}$C-HSQC spectrum of **9** in $CDCl_3$

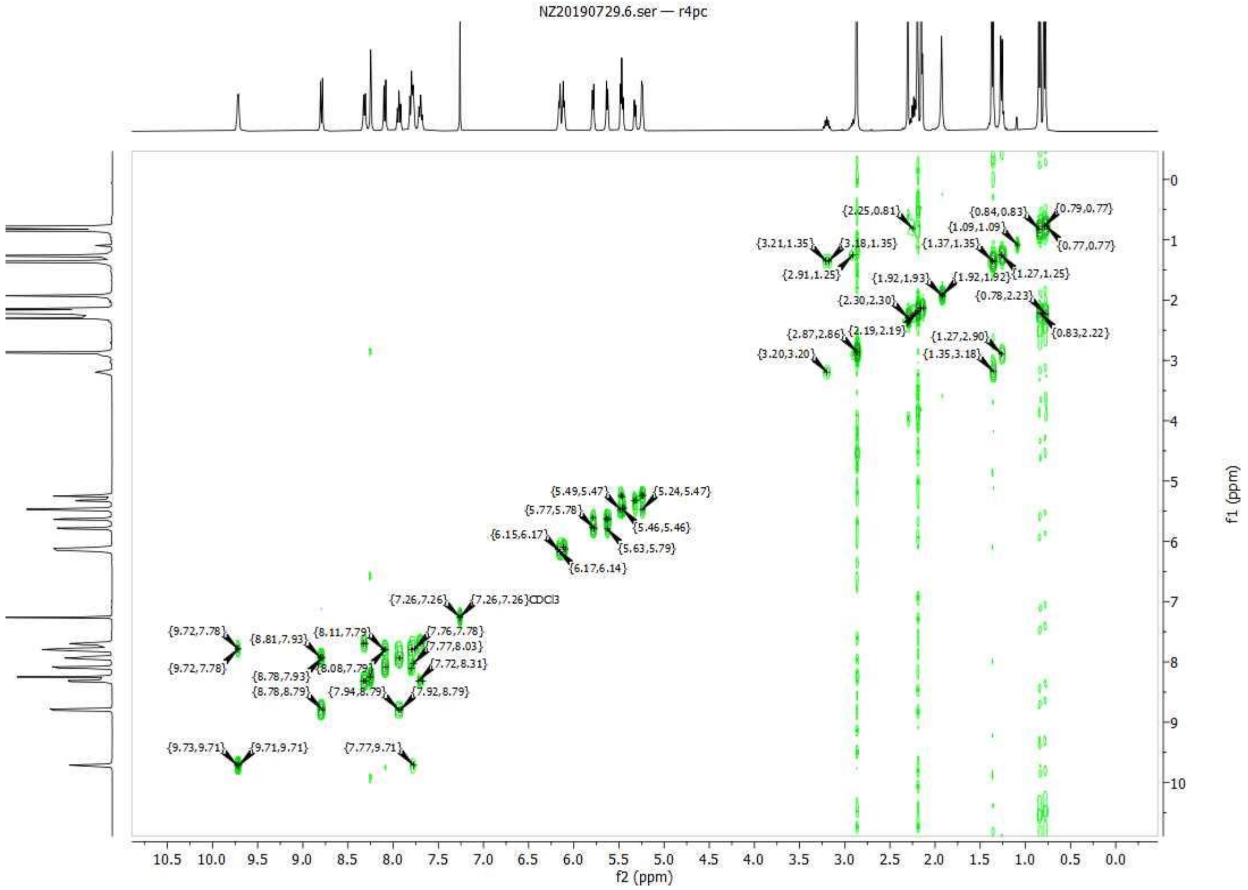

**Figure S69**. $^1$H-$^1$H COSY spectrum of **10** in $CDCl_3$

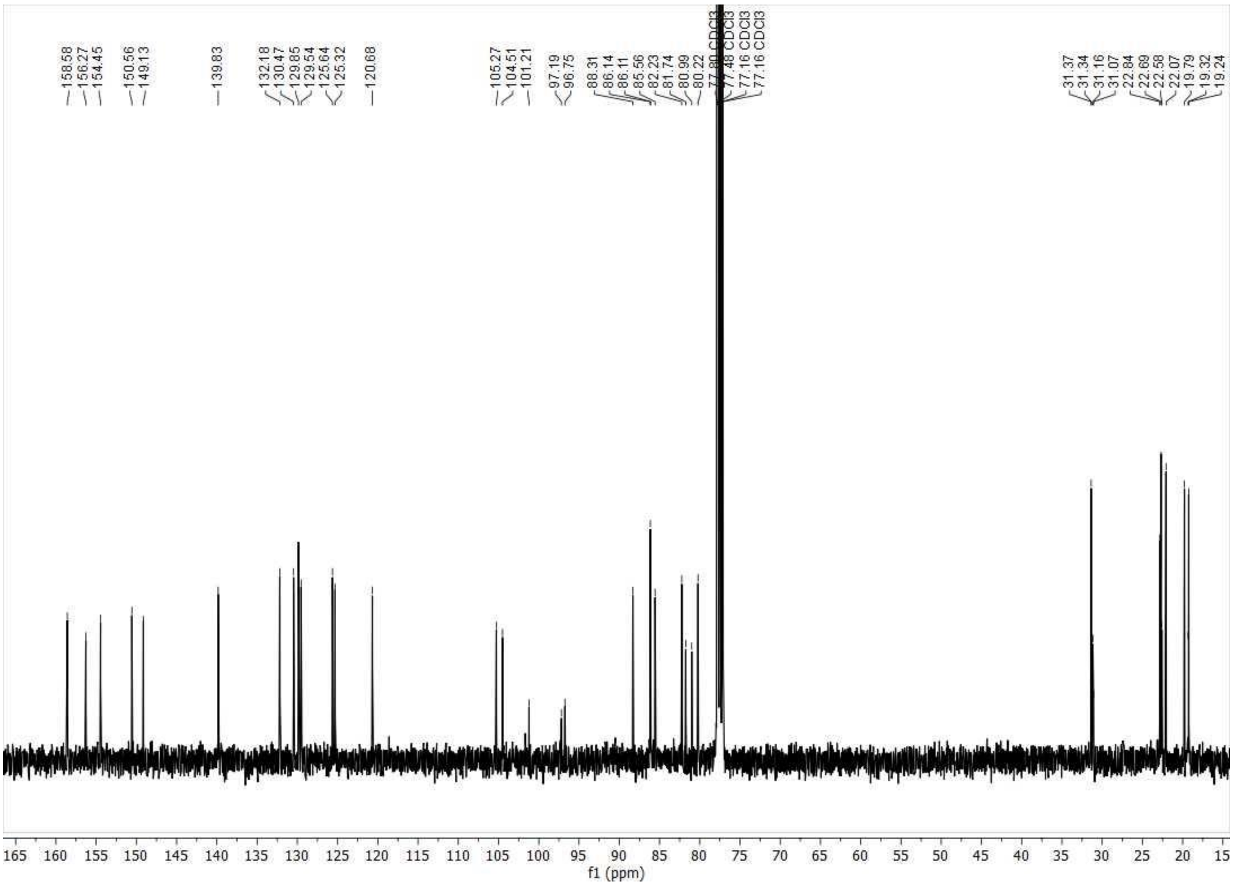


**Figure S70**. $^{13}C\{^{1}H\}$-NMR spectrum of **10** in $CDCl_3$

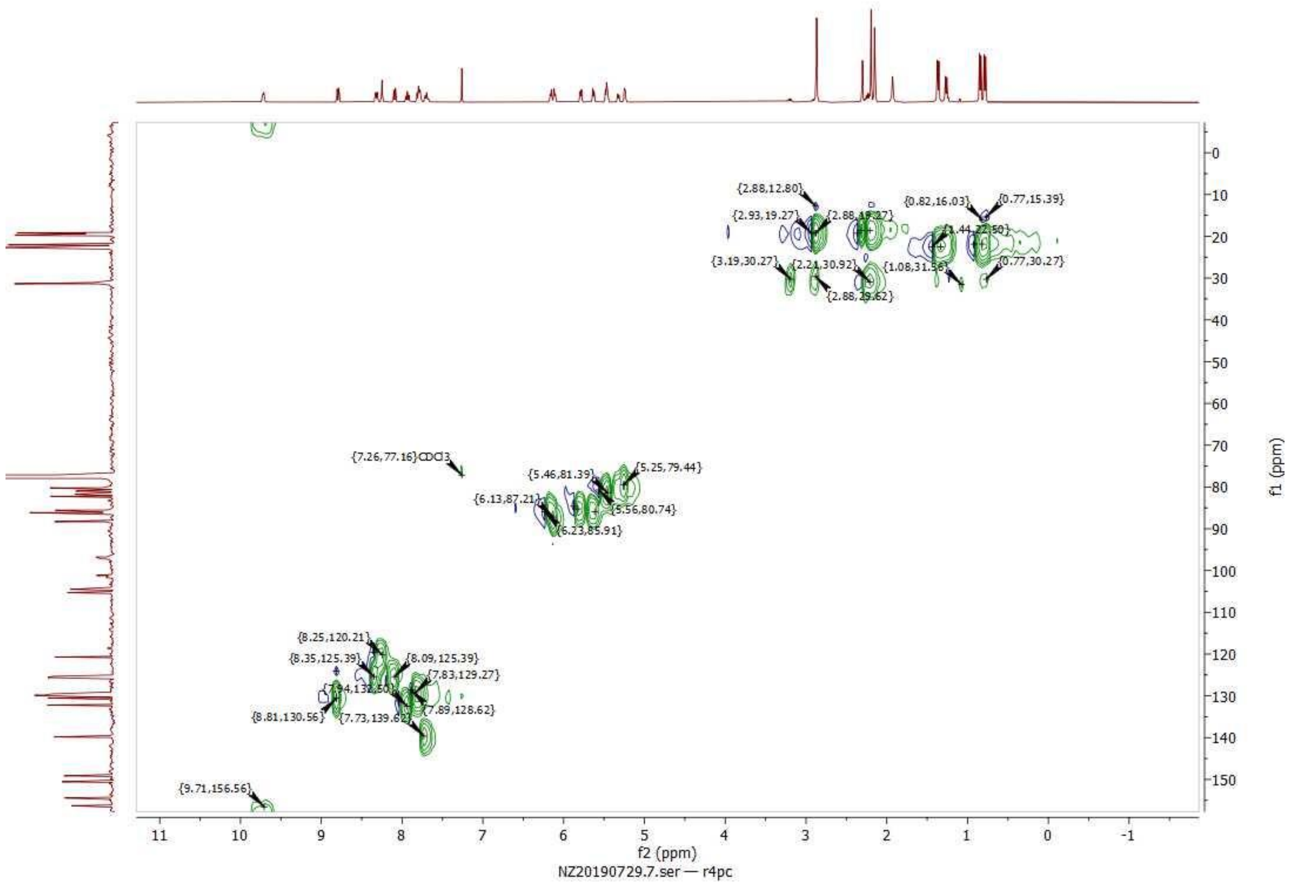


**Figure S71**. $^{1}H$-$^{13}C$-HSQC spectrum of **10** in $CDCl_3$

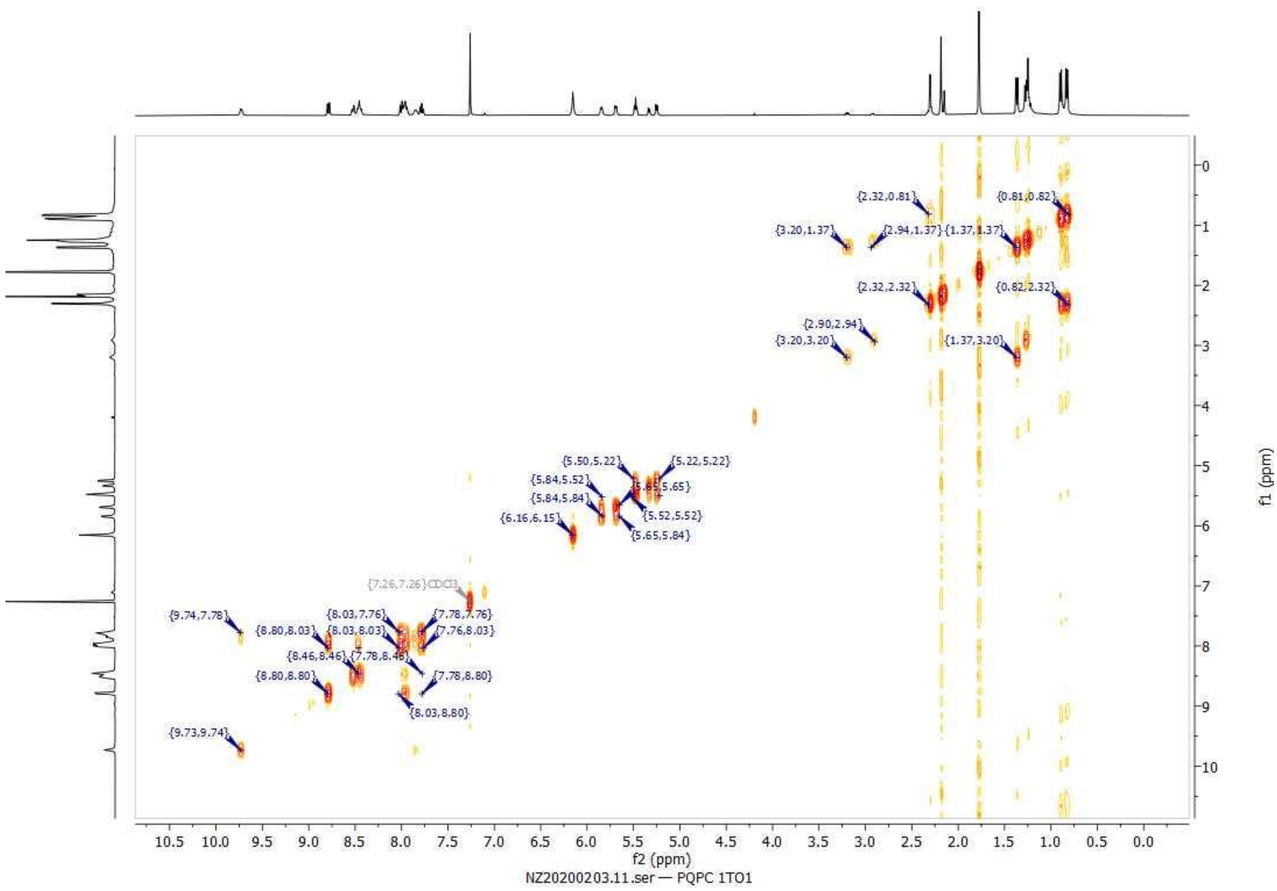


**Figure S72**. $^{1}H$-$^{1}H$ COSY spectrum of **11** in $CDCl_3$

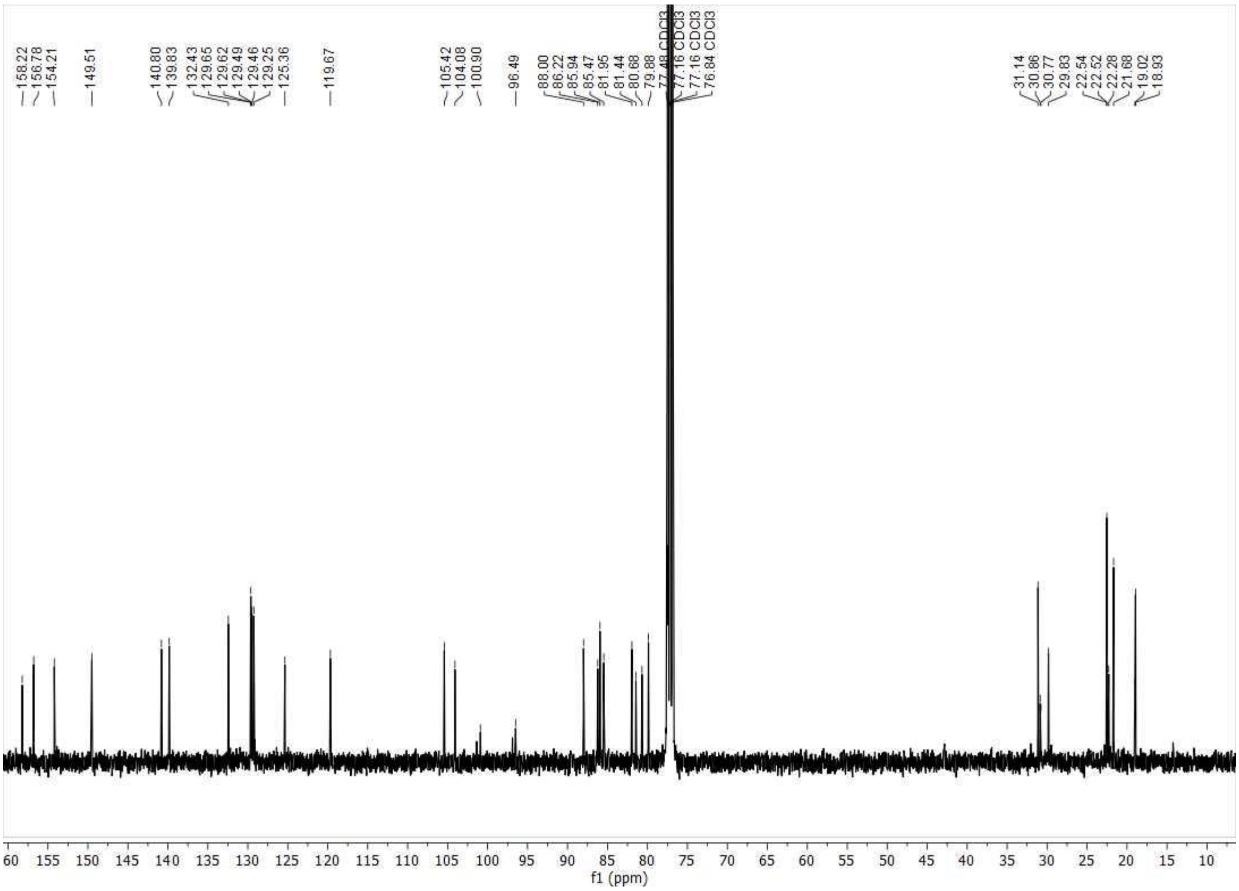


**Figure S73**. $^{13}C\{^1H\}$-NMR spectrum of **11** in $CDCl_3$

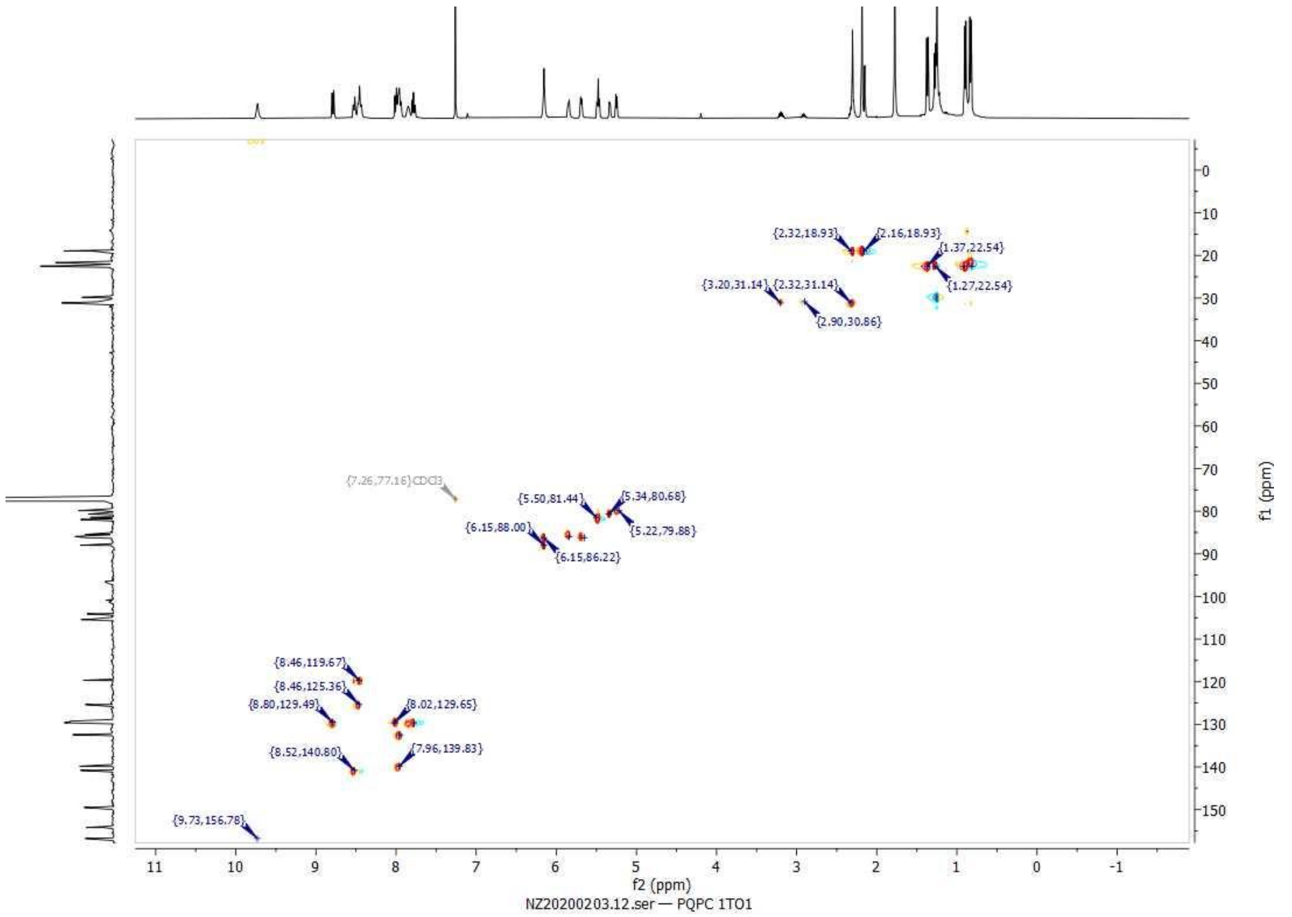


**Figure S74.** $^{1}H$-$^{13}C$-HSQC spectrum of **11** in $CDCl_3$

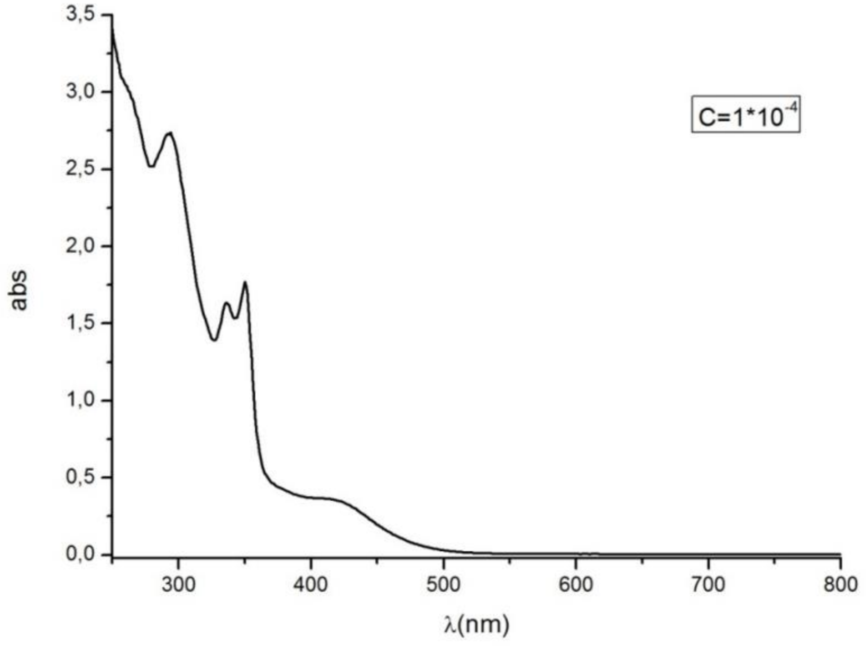


**Figure S75**. UV-vis spectrum of **9** in $CHCl_3$

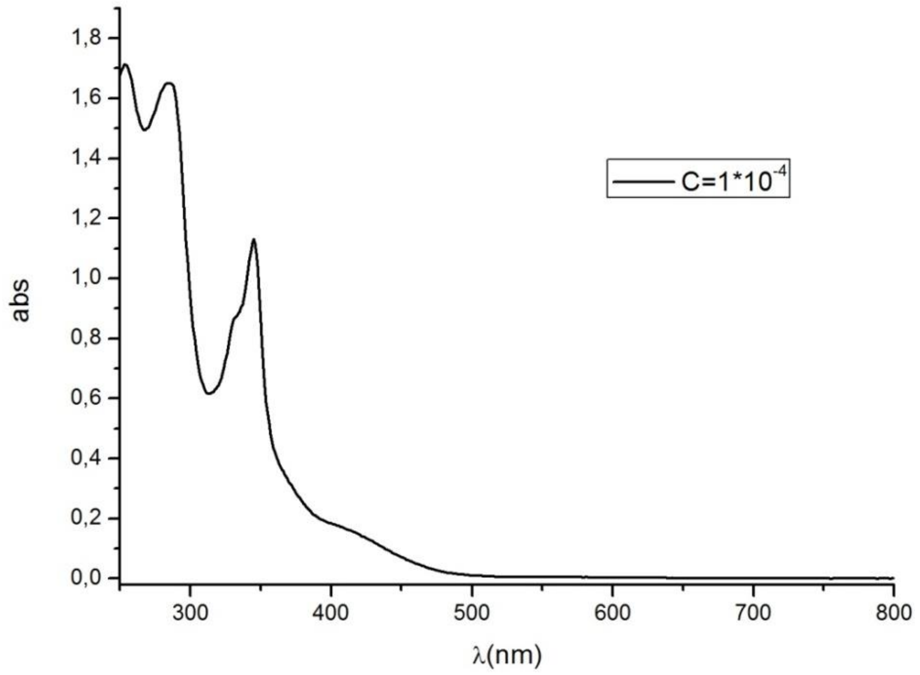


**Figure S76**. UV-vis spectrum of **9** in $H_2O$

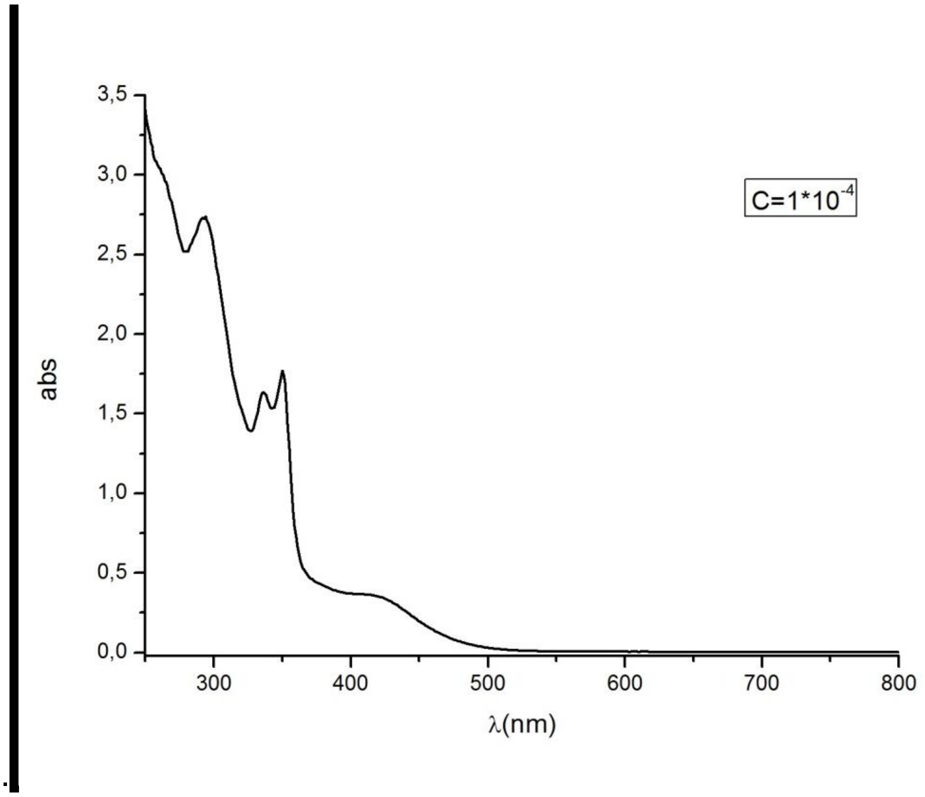


**Figure S77**. UV-vis spectrum of **10** in $CHCl_3$

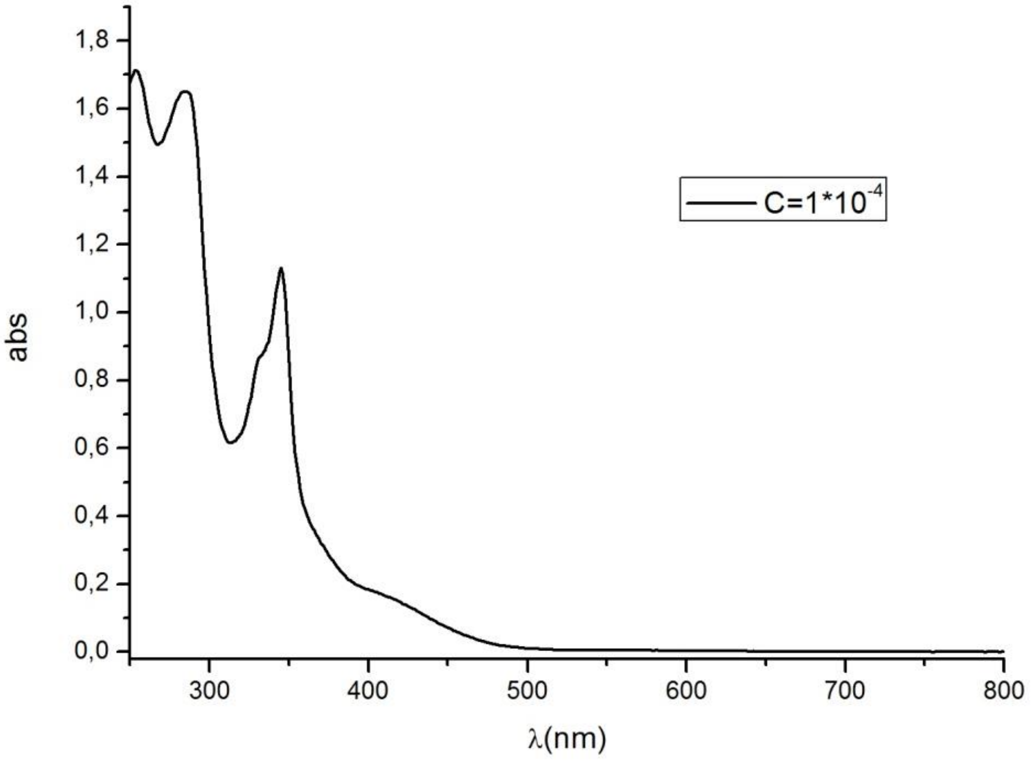


**Figure S78**. UV-vis spectrum of **10** in $H_2O$

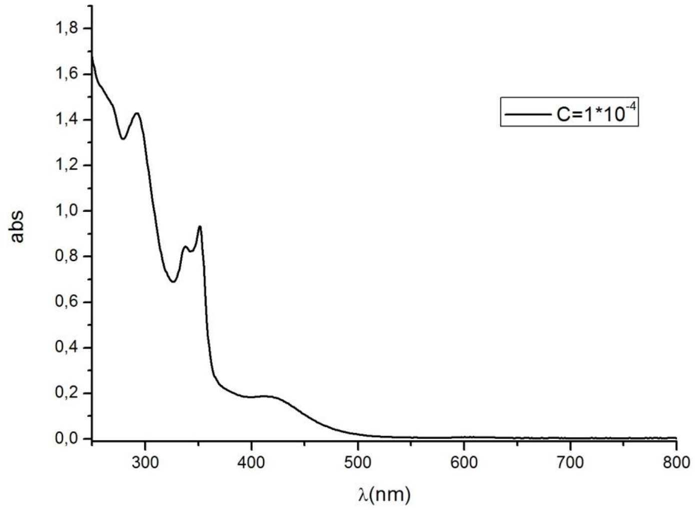


**Figure S79**. UV-vis spectrum of **11** in $CHCl_3$

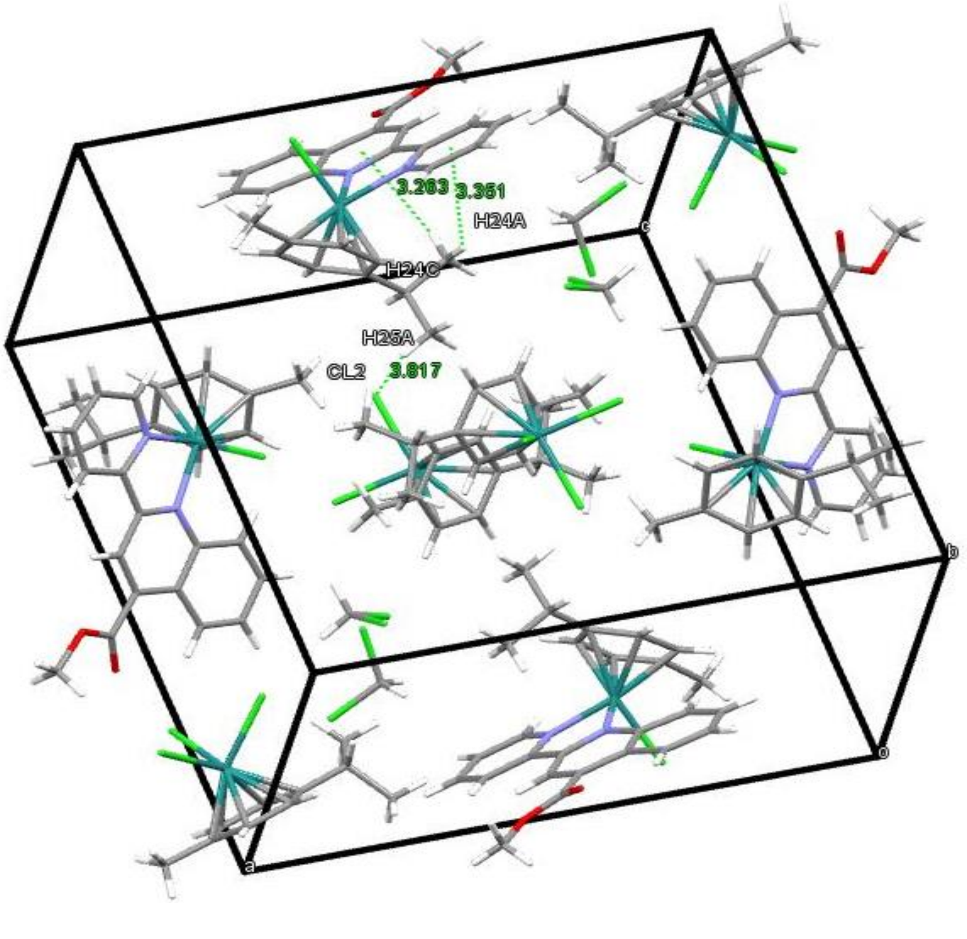


**Figure S80.** Intermolecular interactions in the single crystal of **9**

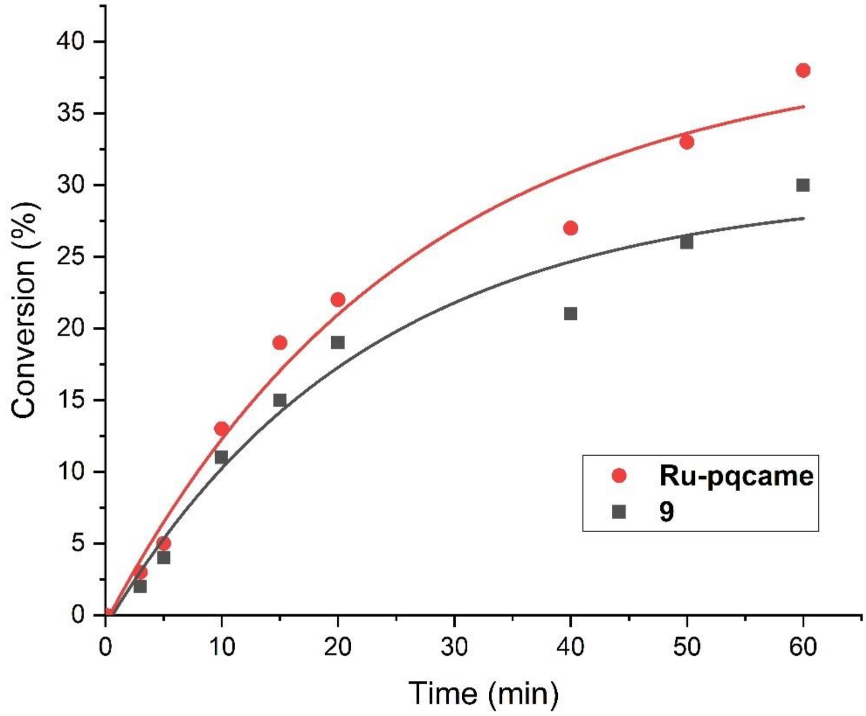


**Figure S81.** Conversion versus reaction time for acetophenone transfer hydrogenation by catalysts **Ru-pqcame** and **9** within 60 min

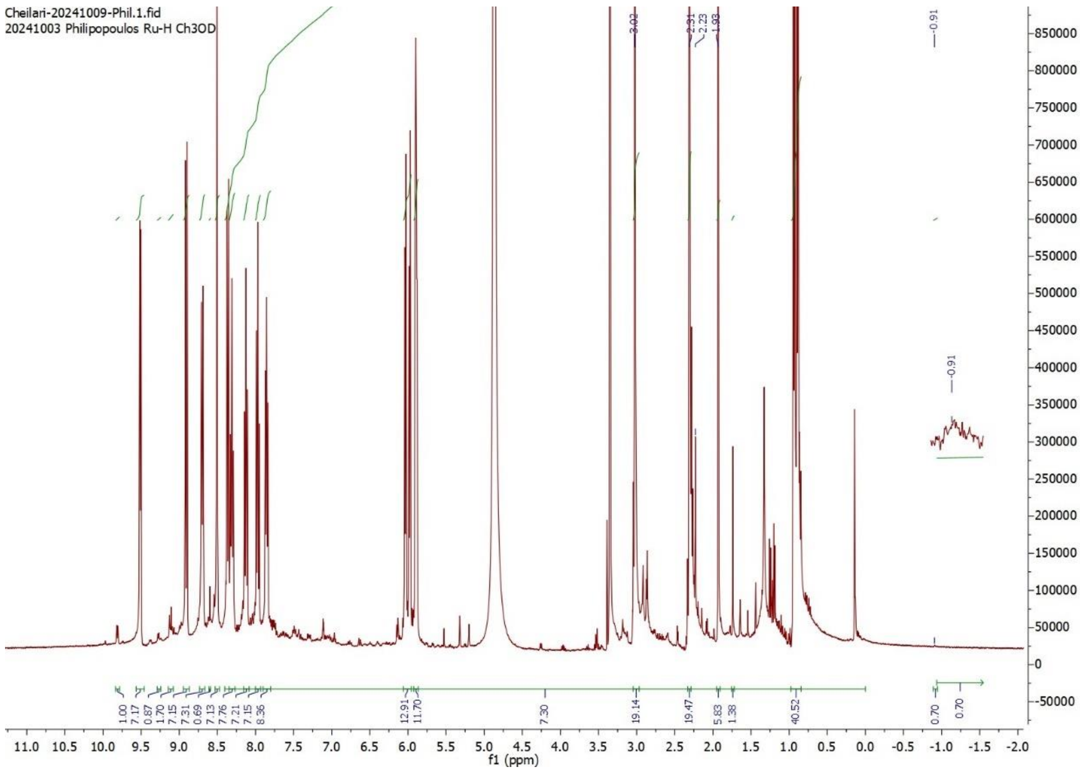


**Figure S82**. $^{1}$H NMR spectrum ($CH_3OD$) of a sample of **4** showing the formation of Ru-H species

**TABLE S2** Crystal and refinement data for **8-Mepq, 4,6′-$Me_2$pqca** and **8,6′- $Me_2$pqca.**

| | **8-Mepq** | **4,6′-$Me_2$pq** | **8,6′-$Me_2$pq** |
|---|---|---|---|
| Empirical formula | $C_{15}H_{12}N_2$ | $C_{16}H_{14}N_2$ | $C_{16}H_{14}N_2$ |
| Molecular weight | 220.27 | 234.29 | 234.29 |
| Crystal color | colourless plate | colourless block | yellow plank |
| Crystal size ($mm^3$) | 0.32 × 0.16 × 0.06 | 0.32 × 0.24 × 0.2 | 0.21 × 0.05 × 0.04 |
| Temperature (K) | 100.01 | 100 | 100 |
| Crystal system | orthorhombic | orthorhombic | orthorhombic |
| Space group | $Pn2_1a$ | $Pbca$ | $P2_12_12_1$ |
| *Unit cell dimensions* | | | |
| *a* (Å) | 22.2546(8) | 9.6069(2) | 4.7706(7) |
| *b* (Å) | 24.9016(9) | 14.6504(3) | 14.830(3) |
| *c* (Å) | 3.92100(10) | 17.8853(4) | 17.148(4) |
| $\alpha$ (°) | 90 | 90 | 90 |
| $\beta$ (°) | 90 | 90 | 90 |
| $\gamma$ (°) | 90 | 90 | 90 |
| $V$ (Å$^3$) | 2172.92(12) | 2517.27(9) | 1213.2(4) |
| $Z$ | 8 | 8 | 4 |
| $\rho_{calc}$ (g cm$^{-3}$) | 1.347 | 2517.27(9) | 1.283 |
| μ ($mm^{-1}$) | 0.627 | 8 | 0.076 |
| $2\theta$ range (°) | 7.1 to 135.498° | 12.076 to 135.488° | 5.986 to 55.998° |
| Index ranges | $-26 \le h \le 26$, $-29 \le k \le 29$, $-4 \le l \le 4$ | $-11 \le h \le 11$, $-17 \le k \le 17$, $-19 \le l \le 21$ | $-6 \le h \le 4$, $-19 \le k \le 19$, $-22 \le l \le 22$ |
| Refllections collected | 64914 | 32014 | 9291 |
| Independent reflections | 3949 | 2261 | 2932 |
| Data/restraints/parameters | 3949/1/309 | 2261/0/165 | 2932/0/165 |
| Goodness-of-fit on $F^2$ | 1.126 | 1.084 | 1.012 |
| Final R indexes [I>2σ (I)] | $R_1$ = 0.0533, $wR_2$ = 0.1379 | $R_1$ = 0.0404, $wR_2$ = 0.1001 | $R_1$ = 0.0621, $wR_2$ = 0.1470 |
| Final R indexes [all data] | $R_1$ = 0.0545, $wR_2$ = 0.1388 | $R_1$ = 0.0424, $wR_2$ = 0.1019 | $R_1$ = 0.1117, $wR_2$ = 0.1794 |
| Largest diff. peak/hole/e Å$^{-3}$ | 0.40/–0.27 | 0.18/–0.25 | 0.27/–0.31 |

**TABLE S3** Crystal and refinement data for **1–4**

| | **1** | **2** | **3** | **4** |
|---|---|---|---|---|
| Crystal Habitus | clear orange block | clear orange block | clear orange plate | clear orange plank |
| Device Type | Bruker X8-KappaApexII | Bruker X8-KappaApexII | Bruker X8-KappaApexII | Bruker X8-KappaApexII |
| Empirical formula | $C_{25}H_{26}ClN_2Ru$ | $C_{25}H_{26}ClF_6N_2PRu$ | $C_{26}H_{28}ClF_6N_2PRu$ | $C_{26}H_{28}ClN_2Ru$ |
| Moiety formula | C25 H26 Cl N2 Ru | C25 H26 Cl N2 Ru, F6 P | C26 H28 Cl N2 Ru, F6 P | C26 H28 Cl N2 Ru |
| Formula weight | 491.00 | 635.97 | 649.99 | 505.02 |
| Temperature/K | 100 | 100 | 100 | 100 |
| Crystal system | orthorhombic | monoclinic | triclinic | monoclinic |
| Space group | Pbca | $P2_1/c$ | P-1 | C2/c |
| a/Å | 17.8445(13) | 9.2097(3) | 14.0446(19) | 27.203(9) |
| b/Å | 13.6553(9) | 23.6119(7) | 14.7713(18) | 13.222(5) |
| c/Å | 19.6773(16) | 12.1242(4) | 15.238(2) | 15.605(6) |
| α/° | 90 | 90 | 111.169(3) | 90 |
| β/° | 90 | 108.8567(9) | 116.028(4) | 106.946(10) |
| γ/° | 90 | 90 | 92.252(4) | 90 |
| Volume/Å$^3$ | 4794.8(6) | 2495.01(14) | 2574.2(6) | 5369(3) |
| Z | 8 | 4 | 4 | 8 |
| $\rho_{calc}$g/cm$^3$ | 1.360 | 1.693 | 1.677 | 1.250 |
| μ/mm$^{-1}$ | 0.778 | 0.863 | 0.838 | 0.696 |
| F(000) | 2008.0 | 1280.0 | 1312.0 | 2072.0 |
| Crystal size/mm$^3$ | 0.24 × 0.19 × 0.08 | 0.15 × 0.12 × 0.08 | 0.09 × 0.05 × 0.03 | 0.12 × 0.07 × 0.04 |
| Absorption correction | empirical | empirical | empirical | empirical |
| Tmin; Tmax | 0.5664; 0.7460 | 0.6552; 0.7462 | 0.5675; 0.7460 | 0.5227; 0.7459 |
| Radiation | MoKα (λ = 0.71073) | MoKα (λ = 0.71073) | MoKα (λ = 0.71073) | MoKα (λ = 0.71073) |
| 2Θ range for data collection/° | 4.288 to 56° | 6.278 to 55.992° | 3.042 to 55.998° | 3.13 to 55.998° |
| Completeness to theta | 0.997 | 0.998 | 0.998 | 0.990 |
| Index ranges | $-23 \le h \le 23$, $-11 \le k \le 18$, $-20 \le l \le 25$ | $-12 \le h \le 12$, $-31 \le k \le 31$, $-15 \le l \le 16$ | $-18 \le h \le 18$, $-19 \le k \le 19$, $-20 \le l \le 20$ | $-35 \le h \le 32$, $-17 \le k \le 17$, $-20 \le l \le 20$ |
| Reflections collected | 40510 | 51000 | 84607 | 18166 |
| Independent reflections | 5771 [$R_{int}$ = 0.1442, $R_{sigma}$ = 0.0874] | 6012 [$R_{int}$ = 0.0250, $R_{sigma}$ = 0.0127] | 12426 [$R_{int}$ = 0.1599, $R_{sigma}$ = 0.1130] | 6392 [$R_{int}$ = 0.1673, $R_{sigma}$ = 0.2225] |
| Data/restraints/ parameters | 5771/288/296 | 6012/0/329 | 12426/0/677 | 6392/108/276 |
| Goodness-of-fit on $F^2$ | 1.177 | 1.056 | 1.055 | 1.033 |
| Final R indexes [I>=2σ (I)] | $R_1$ = 0.1716, $wR_2$ = 0.3795 | $R_1$ = 0.0245, $wR_2$ = 0.0631 | $R_1$ = 0.0785, $wR_2$ = 0.2067 | $R_1$ = 0.1313, $wR_2$ = 0.3085 |
| Final R indexes [all data] | $R_1$ = 0.1862, $wR_2$ = 0.3872 | $R_1$ = 0.0271, $wR_2$ = 0.0650 | $R_1$ = 0.1498, $wR_2$ = 0.2512 | $R_1$ = 0.2182, $wR_2$ = 0.3561 |
| Largest diff. peak/hole / e Å$^{-3}$ | 2.62/-2.37 | 2.25/-0.44 | 1.66/-1.95 | 1.58/-1.61 |

**TABLE S4** Crystal and refinement data for **6**, **8** and **9**

| | **6** | **8** | **9** |
|---|---|---|---|
| Crystal Habitus | clear red plate | clear red plank | clear red plank |
| Device Type | Bruker X8-KappaApexII | Bruker X8-KappaApexII | Bruker X8-KappaApexII |
| Empirical formula | $C_{32}H_{30}N_2O_4F_6PClRu$ | $C_{28}H_{26}N_2F_6PClRu$ | $C_{37}H_{42}Cl_6N_2O_2Ru_2$ |
| Moiety formula | C32 H30 Cl N2 O4 Ru, F6 P | C28 H26 Cl N2 Ru, F6 P | C26 H26 Cl N2 O2 Ru, C10 H14 Cl3 Ru, C H2 Cl2 |
| Formula weight | 788.07 | 672.00 | 961.56 |
| Temperature/K | 100 | 100 | 100 |
| Crystal system | monoclinic | monoclinic | monoclinic |
| Space group | $P2_1/n$ | $P2_1/n$ | $P2_1/n$ |
| a/Å | 12.3138(9) | 12.3889(6) | 18.1466(18) |
| b/Å | 10.7950(9) | 15.0047(8) | 10.6868(11) |
| c/Å | 23.521(2) | 13.8402(7) | 19.736(2) |
| α/° | 90 | 90 | 90 |
| β/° | 98.205(3) | 94.5731(17) | 91.465(3) |
| γ/° | 90 | 90 | 90 |
| Volume/Å$^3$ | 3094.6(4) | 2564.6(2) | 3826.1(7) |
| Z | 4 | 4 | 4 |
| $\rho_{calc}$g/cm$^3$ | 1.691 | 1.740 | 1.669 |
| μ/mm$^{-1}$ | 0.723 | 0.844 | 1.244 |
| F(000) | 1592.0 | 1352.0 | 1936.0 |
| Crystal size/mm$^3$ | 0.09 × 0.09 × 0.02 | 0.19 × 0.11 × 0.1 | 0.12 × 0.04 × 0.02 |
| Absorption correction | empirical | empirical | empirical |
| Tmin; Tmax | 0.6335; 0.7459 | 0.6632; 0.7461 | 0.6136; 0.7462 |
| Radiation | MoKα (λ = 0.71073) | MoKα (λ = 0.71073) | MoKα (λ = 0.71073) |
| 2Θ range for data collection/° | 5.49 to 55.996° | 4.01 to 55.988° | 4.334 to 56° |
| Completeness to theta | 0.998 | 0.999 | 0.998 |
| Index ranges | $-15 \le h \le 16, -14 \le k \le 14, -31 \le l \le 31$ | $-16 \le h \le 16, -19 \le k \le 19, -18 \le l \le 18$ | $-23 \le h \le 23, -14 \le k \le 14, -26 \le l \le 25$ |
| Reflections collected | 40166 | 94212 | 71161 |
| Independent reflections | 7465 [$R_{int}$ = 0.1238, $R_{sigma}$ = 0.1000] | 6192 [$R_{int}$ = 0.0494, $R_{sigma}$ = 0.0184] | 9211 [$R_{int}$ = 0.1116, $R_{sigma}$ = 0.0660] |
| Data/restraints/parameters | 7465/42/484 | 6192/36/421 | 9211/0/449 |
| Goodness-of-fit on F$^2$ | 0.975 | 1.081 | 1.074 |
| Final R indexes [I>=2σ (I)] | $R_1$ = 0.0571, $wR_2$ = 0.1445 | $R_1$ = 0.0299, $wR_2$ = 0.0668 | $R_1$ = 0.0494, $wR_2$ = 0.1211 |
| Final R indexes [all data] | $R_1$ = 0.1071, $wR_2$ = 0.1741 | $R_1$ = 0.0389, $wR_2$ = 0.0732 | $R_1$ = 0.0879, $wR_2$ = 0.1487 |
| Largest diff. peak/hole / e Å$^{-3}$ | 1.13/-1.34 | 0.88/-0.68 | 1.39/-1.90 |
